# Deconstruction of Archaeal Genome Depict Strategic Consensus in Core Pathways Coding Sequence Assembly

**DOI:** 10.1371/journal.pone.0118245

**Published:** 2015-02-12

**Authors:** Ayon Pal, Rachana Banerjee, Uttam K. Mondal, Subhasis Mukhopadhyay, Asim K. Bothra

**Affiliations:** 1 Department of Botany, Raiganj College (University College), Raiganj, Uttar Dinajpur, West Bengal, India; 2 Department of Biophysics, Molecular Biology and Bioinformatics, University of Calcutta, Kolkata, West Bengal, India; 3 Cheminformatics Bioinformatics Laboratory, Department of Chemistry, Raiganj College (University College), Raiganj, Uttar Dinajpur, West Bengal, India; Tel Aviv University, ISRAEL

## Abstract

A comprehensive *in silico* analysis of 71 species representing the different taxonomic classes and physiological genre of the domain Archaea was performed. These organisms differed in their physiological attributes, particularly oxygen tolerance and energy metabolism. We explored the diversity and similarity in the codon usage pattern in the genes and genomes of these organisms, emphasizing on their core cellular pathways. Our thrust was to figure out whether there is any underlying similarity in the design of core pathways within these organisms. Analyses of codon utilization pattern, construction of hierarchical linear models of codon usage, expression pattern and codon pair preference pointed to the fact that, in the archaea there is a trend towards biased use of synonymous codons in the core cellular pathways and the Nc-plots appeared to display the physiological variations present within the different species. Our analyses revealed that aerobic species of archaea possessed a larger degree of freedom in regulating expression levels than could be accounted for by codon usage bias alone. This feature might be a consequence of their enhanced metabolic activities as a result of their adaptation to the relatively O^2^-rich environment. Species of archaea, which are related from the taxonomical viewpoint, were found to have striking similarities in their ORF structuring pattern. In the anaerobic species of archaea, codon bias was found to be a major determinant of gene expression. We have also detected a significant difference in the codon pair usage pattern between the whole genome and the genes related to vital cellular pathways, and it was not only species-specific but pathway specific too. This hints towards the structuring of ORFs with better decoding accuracy during translation. Finally, a codon-pathway interaction in shaping the codon design of pathways was observed where the transcription pathway exhibited a significantly different coding frequency signature.

## Introduction

Genomes are usually envisaged as an assemblage of the information flowing through an organism’s biological pathway and forms networks in a coordinated manner, encoded by codons in DNA [1]. The enormous variety of genomes that we see around is probably produced by a number of different mechanisms that includes variation in gene order, GC content and codon usage bias or CUB [2]. CUB in a genome is observed due to non-uniform usage of synonymous codons for those amino acids which are coded using a degenerate set. As a result, there is a skewed distribution of certain codons within the coding sequences of a large number of organisms [3]. As CUB had its origin in the degenerate nature of the genetic code, it is, so to say, a reflection of equilibrium among natural selection, mutation and genetic drift [4–8]. CUB is widespread across species known to introduce biological consequences on varied cellular processes and may contribute towards genome evolution in a significant manner [3,9]. Comparative analyses have confirmed that different organisms display certain preference towards specific codon pairs in their genomes [10–12]. In genes of bacteria, the over-represented codon pairs are translated slower than under-represented codon pairs [13]. By deconstructing the genome into its constituting pathways and studying the individual pathway coding sequences, we explored the diversity in the codon usage design of core and vital metabolic pathways and information processing system. These analyses might strengthen our understanding about the existence of pathway-specific codon usage pattern, in extremophilic organisms like most archaeal species, where priority is maximized for off-setting the extreme environment. This may have a manifestation in the form of a tolerance to stresses, like extreme salt concentrations, pH, temperature etc. The archaea represent a unique group of microorganisms inhabiting some of the most extreme environments of this planet along with other “normal” niches viewed from an anthropocentric stance [14,15]. These unique microorganisms evolved several billion years ago as one of the three primary lineages or domains of life [16]. The gradual availability of a large number of complete genome sequences of archaea has opened new lines of enquiry involving archaea, especially in the area of comparative genomics and proteomics. There has been some comparative study on the proteome and amino acid usage pattern of the domain Archaea [17–19] and the whole genome codon usage pattern. The major portion of the predicted highly expressed (PHX) genes in 19 archaeal genomes, have been analyzed, on the basis of their codon usage bias [20] and have been compared with eubacteria, yeast and *Drosophila* [21]. The study of the tendencies as regards codon usage towards content bias, translational bias and strand bias have been applied in 16 archaeal species to identify preferred codons in the genome which acts as the codon bias signature [22]. The effect of the G+C content in the coding region of 36 archaeal genomes have been analyzed [23]. Emery and Sharp [24] have studied the impact of translational selection on codon usage bias in a methanogenic archaeon, *Methanococcus maripaludis*. Lynn *et al*., had analyzed synonymous codon usage variation among bacteria and a few species of archaea based on temperature tolerance [25]. The codon usage bias in archaea have also been studied to detect the variation in global (i.e., whole genome) codon usage bias and its association with the lifestyle of an organism [26]. But, a deep analysis is required for the large scale comparative analysis of codon usage pattern, codon context analysis and genome design, specifically when dealing with the major housekeeping biological pathways of the domain Archaea. A significant facet of metabolism is the similarity of the key metabolic pathways amongst diverse species probably due to their early advent in evolutionary history or due to their greater efficiency in preferring the function. Therefore, it is important to carry out a comparative analysis of metabolic pathways among diverse species with a view to extracting information about the functional relation of organisms. Seventy one species of archaea (S1 Table) distributed across the phyla Euryarchaeota, Crenarchaeota, Thaumarchaeota, Korarchaeota, Nanoarchaeota and representing all the major classes of archaea living in extreme environment and exhibiting diverse physiological features, in terms of oxygen tolerance and energy metabolism, were subjected to exhaustive analysis for revealing codon utilization bias, codon pair bias and expression level in this study.

The pathways considered in our analysis include amino acid metabolism (AAM), carbohydrate metabolism (CM), nucleotide metabolism (NM), energy production and conversion pathways (EPC) and the vital genetic information processing system of transcription (Tr), since these are the vital and universal requirements of every living cell. Construction of generalized and hierarchical linear models with respect to codon usage, expression pattern and codon pair preference and studying the interrelationships between the different codon usage indices of vital pathways may unravel the synergy and contribution of core pathways towards overall genomic codon usage and design optimization.

## Results and Discussion

### Genome deconstruction to explore the aspect of codon usage i.e., ‘codonscape’

The major emphasis of the study was to deconstruct the entire genome into its major constituent pathways and study the codon usage design in terms of the individual pathways. A clear pattern may be detected for conserved use of codons in the core house-keeping pathways and decipher the trends revealing the overall codon usage design or what we prefer to call the ‘codonscape’ of the genome. In order to decipher the codon usage design in the domain Archaea, we utilized such important parameters as the effective number of codons or Nc [27,28], guanine and cytosine content at third position of the codon or GC3s [29,30], codon adaptation index or CAI [31,32], synonymous codon usage order or SCUO [33,34] and frequency of optimal codons or Fop [35,36].

### Estimation of codon usage bias

**Fig 1:**
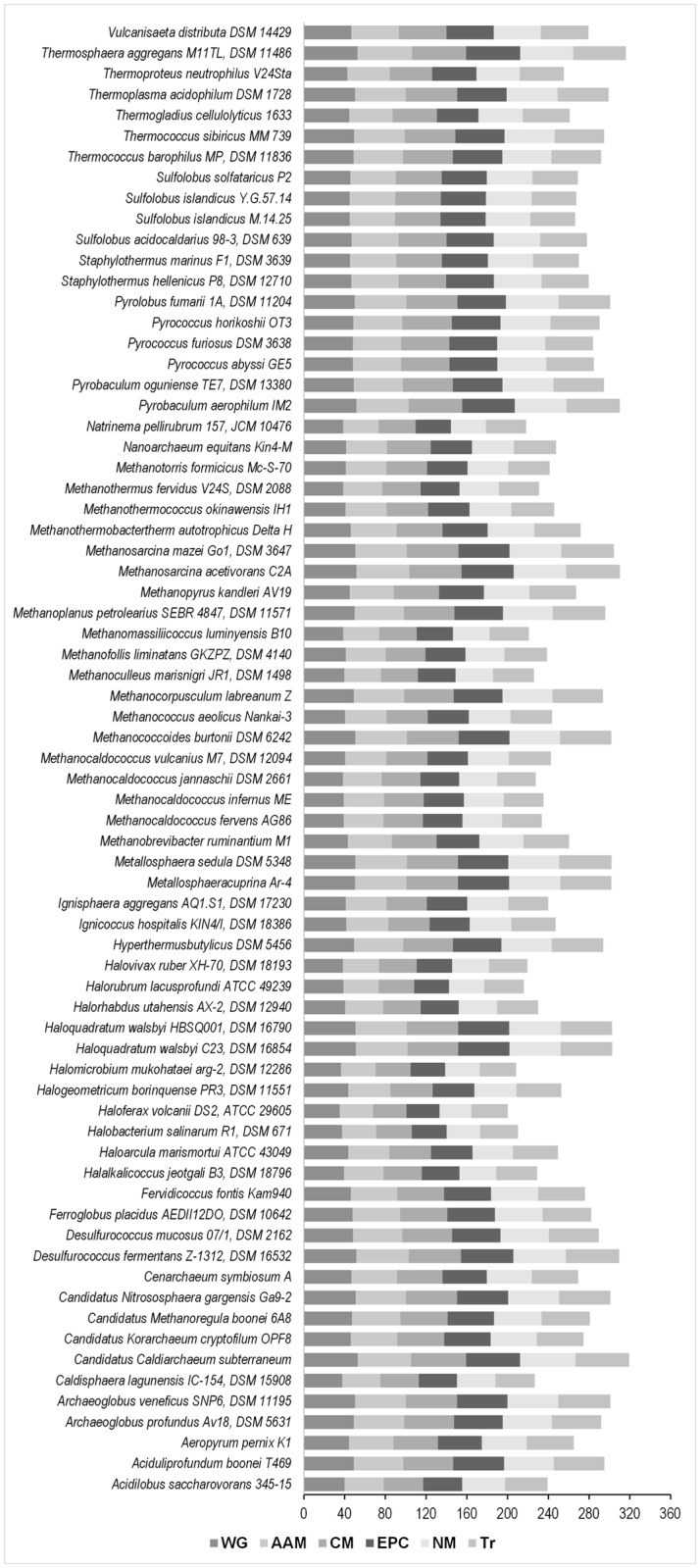
A comparative account of the average effective number of codons (Nc_avg) of each organism across the whole genome and the major biological pathways. The average Nc of the whole genome (Nc_avg[G]_) was found to be close to 40 for nearly the fifty percent of the organisms considered in this study. In species like *Caldisphaera lagunensis* IC-154, DSM 15908, *Halobacterium salinarum* R1, DSM 671, *Haloferax volcanii* DS2, ATCC 29605, *Halomicrobium mukohataei* arg-2, DSM 12286, the Nc_avg[G]_ was found to be below 40.

Initially, the degree of codon bias present in the 71 archaea species was estimated by calculating the effective number of codons or Nc. The average Nc of the whole genome (Nc_avg[G]_) was found to be close to 40 for nearly the fifty percent of the organisms considered in this study (Fig. 1). In species like *Caldisphaera lagunensis* IC-154, DSM 15908, *Halobacterium salinarum* R1, DSM 671, *Haloferax volcanii* DS2, ATCC 29605, *Halomicrobium mukohataei* arg-2, DSM 12286, the Nc_avg[G]_ was found to be below 40. An Nc value of less than forty (Nc<40) is considered as the hallmark of major codon usage bias [37–41], suggesting a significant codon usage bias at the whole genome level. The Nc of the whole genome (Nc_[G]_) was compared with the Nc of the coding sequences of the five biological pathways using Kruskal-Wallis one-way ANOVA on ranks and the Nc values between the whole genome and the different biological pathway coding sequences was substantially different (*H* = 565.80, 5 d. f.) at p < 0.001 level. Post facto analysis using Dunn’s procedure with a Bonferroni correction for multiple comparisons revealed statistically significant differences in Nc values between the whole genome and the coding sequences of other pathways except the transcription pathway system. This suggests that in the archaea, there is a trend towards a biased use of synonymous codons in the coding sequences of core housekeeping metabolic pathways. To further substantiate this finding, a Mann–Whitney *U* test was performed to compare the Nc of the whole genome (Nc_[G]_) with the Nc of the coding sequences of the five major biological pathways and the distribution of Nc values between the whole genome and the different metabolic pathway coding sequences was found to be substantially different at p<0.001 level. This conclusively suggests that in the archaea, there is a trend towards a biased use of synonymous codons in the coding sequences of core metabolic pathways. The Mann–Whitney test too, reported the absence of statistically significant differences in Nc values between the whole genome and that of the coding sequences of the genetic information processing transcription system (Mann–Whitney *U =* 724229784.00, p = 0.426).

### The Nc-GC3 relationship trend as fingerprints of physiological variations

**Fig 2:**
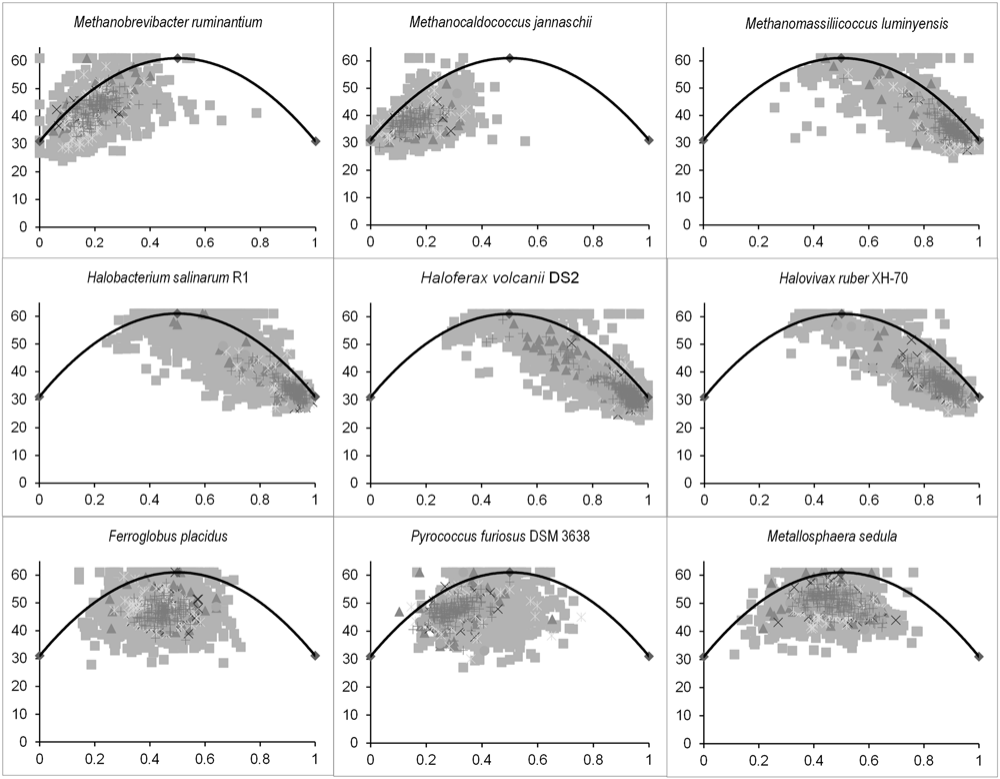
The Nc-plots from different archaea species, acting as fingerprints of physiological variations. The *x*-axis and the *y*-axis represents the GC3 and Nc values respectively. The gene sequences of the different systems are represented by the following symbols—■ = Genome, ▲ = Transcription (Tr), × = Nucleotide metabolism (NM), * = Energy processing and conversion, ● = Carbohydrate metabolism, + = Amino acid metabolism (AAM). The methanogens are characterized by a left centric Nc-plot except for *Methanomassiliicoccus luminyensis*. The contrarian positioning of *Methanomassiliicoccus luminyensis* on the Nc plot with respect to the other methanogens is a reflection of one of its many peculiar attributes in comparison with other methanogens. The halophiles display a consensus right centric aggregation of coding sequences on the Nc plot exemplified by *Halobacterium salinarum*, *Halovivax ruber* and *Haloferax volcanii*. The mid centric feature is characteristic of the thermo and hyperthermophiles like *Pyrococcus spp*., *Ferroglobus placidus*, *Metallosphaera sedula* etc. The continuous curve represents the null hypothesis that that the GC bias at the synonymous site is solely due to mutation but not selection.

Information regarding intra-species and inter-species synonymous codon usage variation can be accounted for by studying the variation in G+C content in the third position of a codon. One usually employs a Nc-plot [27] to explore such intra- and inter-specific synonymous codon usage patterns [30,42]. Nc-plots depicting the relative positioning of the vital pathway genes in the vast ocean of the whole genome for some of the archaea species included in this study are shown in Fig. 2. This figure aptly demonstrates the emergence of three distinct patterns. The methanogens are characterized by a left centric Nc-plot, except for *Methanomassiliicoccus luminyensis* which unlike other methanogens cannot produce methane using hydrogen or methanol as the sole energy source. *M*. *luminyensis* displays horizontal gene transfer events for adaptation and evolution in the human ecosystem. It has the largest genome (2.05 Mb) among the human associated archaea with a genomic GC content of 59.93 mol% [43,44]. The rather deviant Nc-plot pattern of *M*. *luminyensis* is thus a pointer towards its unique physiological and genomic properties. The halophiles display a consensus right centric aggregation of coding sequences on the Nc plot for the organisms *Halobacterium salinarum*, *Halovivax ruber* and *Natrinema pellirubrum*. The mid centric feature in the figure is a characteristic of the thermo and hyperthermophiles like *Pyrococcus spp*., *Ferroglobus placidus*, *Metallosphaera sedula* etc. The Nc-plots, thus, can be taken to be fingerprints for figuring out the observed physiological variations prevailing in varied archaea species.

**Fig 3:**
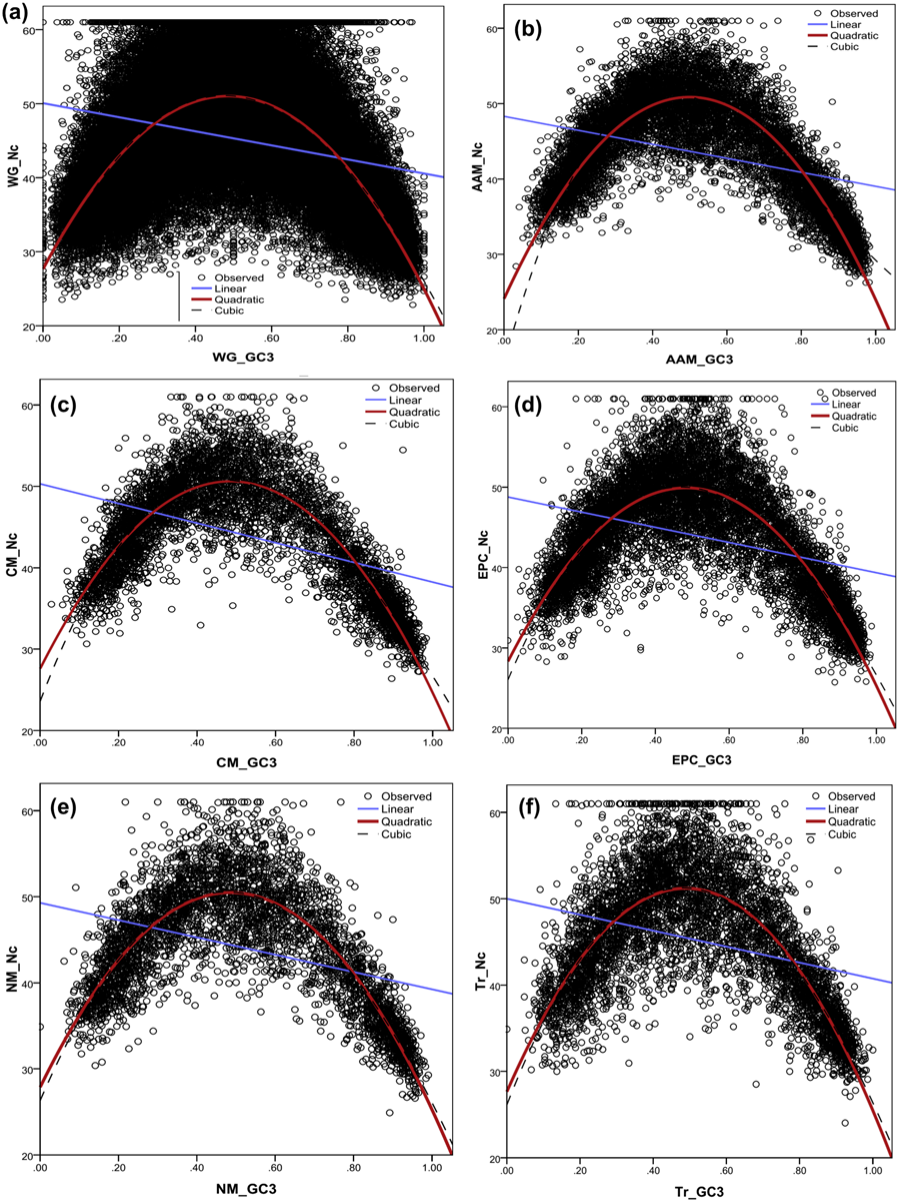
Plots depicting the non-linear relationship between GC3 with Nc for the whole genome and the vital pathways. The independent variable GC3 is observed here to be non-linearly related with the dependent variable Nc in all the six cases (2a to 2f) and a quadratic regression equation accounts best for the variability between the two variables. In the case of whole genome (2a), GC3 explained 56% of the variability in codon bias or Nc, the coefficient of variance or *R* ^2^ = 0.56, at p < 0.001 level (predicted Nc = 27.66 + 96.04 * GC3 + (-98.72) * GC3^2^). The *R* ^2^ value improved significantly for the Nc-GC3 relationship when only the vital pathways were considered (2b to 2f). This is true for the amino acid metabolism (*R* ^2^ = 0.73, p <0.001), carbohydrate metabolism (*R* ^2^ = 0.756, p <0.001), energy processing and conversion (*R* ^2^ = 0.655, p <0.001), and nucleotide transport and metabolism pathways (*R* ^2^ = 0.71, p <0.001). The transcription pathway (*R* ^2^ = 0.60, p <0.001) reflected the trend depicted by the whole genome where about 60% of the Nc score variability is explained by GC3 through the non-linear quadratic model.

Spearman’s correlation between Nc and GC3 of the individual genes coding for the major biological pathway and the whole genome sequences depicted a weak to moderate, but a consistent anti-correlation (Table 1). The independent variable GC3 was found to be non-linearly related with the dependent variable Nc for all the six cases and a quadratic regression equation accounts best for the variability between the two variables. This may imply that selective forces are playing a significant role towards fine tuning the translational efficiency for the vital metabolic pathways. In the case of whole genome (Fig. 3a), 56% of the variability as found in codon bias or Nc, was explained by GC3, in which the coefficient of variance or *R* ^2^ = 0.56, at p<0.001 level. The *R* ^2^ value improved significantly for the Nc-GC3 relationship when only the vital pathways were considered (Fig. 3b to 3f). This led to better models explaining a greater amount of variability in Nc for the variation in G+C content in the third position of a codon. This is true for the amino acid metabolism (*R* ^2^ = 0.73, p <0.001), carbohydrate metabolism (*R* ^2^ = 0.756, p <0.001), energy processing and conversion (*R* ^2^ = 0.655, p <0.001), and nucleotide transport and metabolism pathways (*R* ^2^ = 0.71, p<0.001). The transcription pathway (*R* ^2^ = 0.60, p<0.001) showed the same trend as is depicted by the whole genome, where about 60% of the Nc score variability is explained by GC3 using the non-linear quadratic model.

**Table 1:** Genome wise and pathway wise correlation (Spearman’s rank-order) between Nc and GC3.

Genome/Pathway wise Nc-GC3 correlation	Correlation Coefficient (*ρ*)	Significance (2-tailed)	Number of gene sequences (N)
Whole Genome	-0.29	p < 0.001	161014
Amino acid metabolism	-0.37	p < 0.001	10095
Carbohydrate metabolism	-0.36	p < 0.001	5141
Energy processing and conversion	-0.28	p < 0.001	9538
Nucleotide metabolism	-0.3	p < 0.001	3870
Transcription	-0.27	p < 0.001	5474

### Synonymous codon usage order and frequency of optimal codons as a CUB indicator

Codon usage bias is reported to be affected by GC composition and environment [45] and since archaea dwell in a wide range of environment, most of which may be regarded as ‘stressed’ one, we analyzed the SCUO parameter. The SCUO of the 71 archaea species was compared by grouping them together based on the five different pathways and using Kruskal-Wallis ANOVA on Ranks with a pairwise comparison by Dunn’s method with Bonferroni correction. Using these analyses, we found a statistically significant difference in the SCUO scores between the whole genome and the coding sequences of the pathways of amino acid metabolism, carbohydrate metabolism and energy processing and conversion system (*H* = 654.84, 5 d. f.) at p<0.001 level. A Mann–Whitney *U* test further validated our findings and a statistically significant difference in the SCUO scores between the whole genome and the coding sequences of the pathways of amino acid metabolism (Mann–Whitney *U =* 735631350.00, p<0.001), carbohydrate metabolism (Mann–Whitney *U =* 355377537.00, p<0.001) and energy processing and conversion system (Mann–Whitney *U =* 707605235.50, p<0.001). Like Nc, here too, we did not find any statistically significant difference in SCUO values between the whole genome and the transcription pathway.

**Fig 4:**
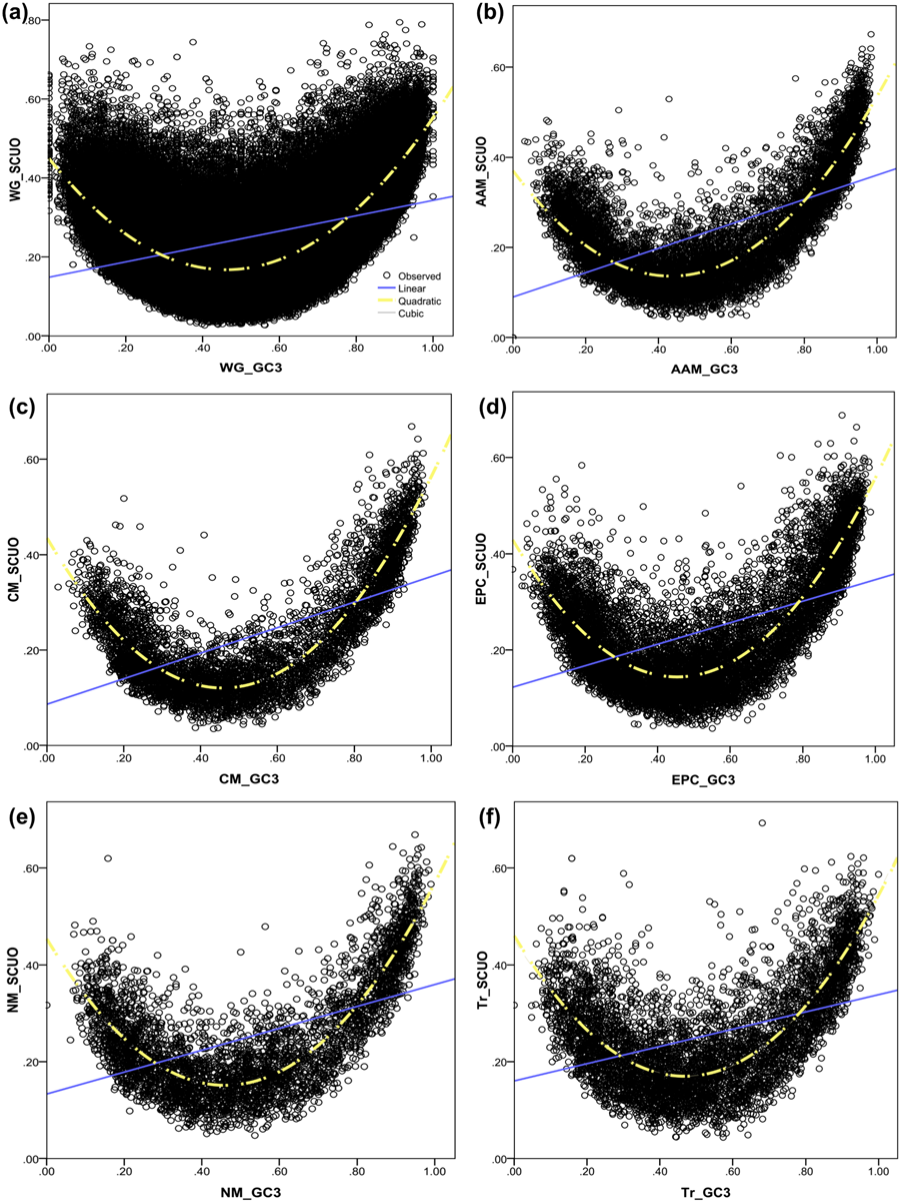
Plots depicting the inter-relationship between SCUO and GC3 for the whole genome along with the vital biological pathways. A non-linear relationship between the two parameters SCUO and GC3 was observed. Quadratic regression models were best found to describe the variability in SCUO by GC3. The best fit models were obtained (3a to 3f) for the vital biological pathways explaining a higher percentage of SCUO variation by GC3 in comparison to the whole genome SCUO-GC3 model. The transcription pathway model in Fig. 3f (*R* ^2^ = 0.59, p < 0.001) was found to faithfully replicate the behavior of the whole genome SCUO-GC3 model (*R* ^2^ = 0.54, p < 0.001) suggesting its influence in determining the overall codon usage design (3f).

Pathway specific inter-relationship between SCUO and GC3 was analyzed and it was observed that a non-linear relationship exists between the two parameters. Quadratic regression models were found to describe the variability in SCUO by GC3 as the best possible model in this case. Similar to Nc-GC3 relationship, the best fit models were obtained (Fig. 4a to 4f) for the vital biological pathways explaining a higher percentage of SCUO variation by GC3 in comparison to the SCUO-GC3 model of the whole genome. The non-linear relationship between SCUO and GC3 was in conformity with the trend detected by Wan *et al*. [34] that depicted a quantitative relationship between codon usage bias and GC3 composition in 86 microbial genomes and also in line with the trend observed in mammals like mouse and humans [46]. The transcription pathway model (*R* ^2^ = 0.59, p < 0.001) was found to quite faithfully replicate the behavior of the whole genome as depicted by SCUO-GC3 model (*R* ^2^ = 0.54, p < 0.001). In this case an influence in determining the overall codon usage design is suggested (Fig. 4f).

A cardinal parameter in understanding the nature of codon usage bias was the frequency of optimal codons (Fop). The frequency of optimal codons in a gene helps one in estimating the strength of the past selection on codon usage [36]. We have estimated the Fop values for all the gene sequences of the 71 organisms and observed a statistically significant difference in the Fop values by both Mann–Whitney Rank Sum test and a Kruskal-Wallis ANOVA on ranks between the whole genome and the different pathways at p < 0.001 level.

**Fig 5:**
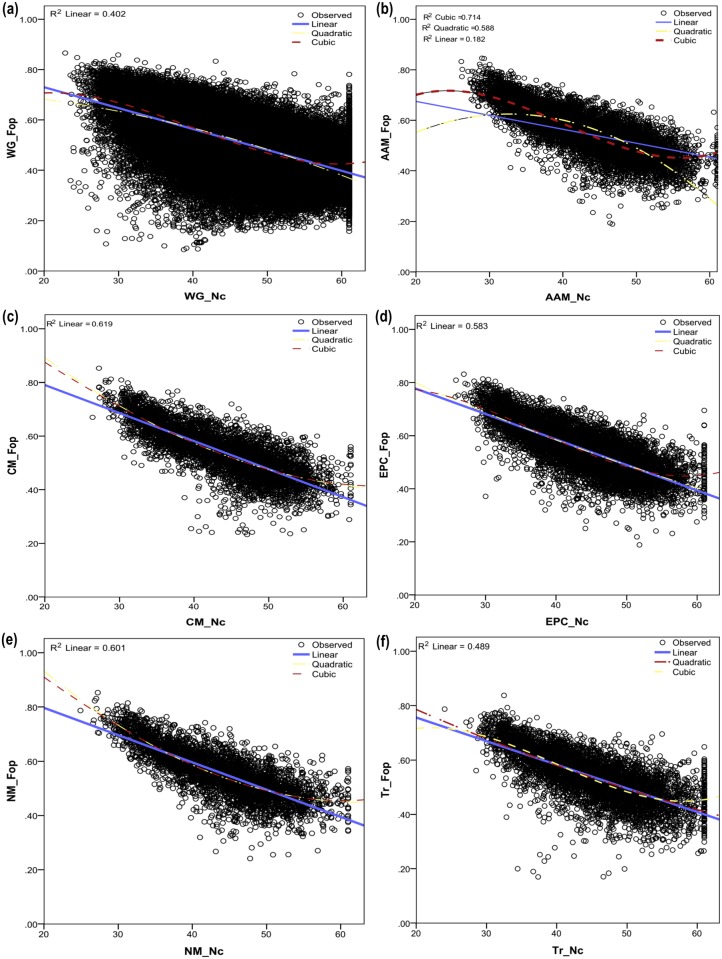
Plots depicting the association between Nc and Fop for the whole genome along with the vital biological pathways. An interesting finding was the non-linear association of the Nc and Fop values of the amino acid metabolism pathway gene sequences (4a). A non-linear cubic regression curve describes best the Fop variability (*R* ^2^ = 0.714, p < 0.001), which is about 71%, using Nc (predicted Fop = 0.006 + 0.068 * Nc + (-0.002) * Nc^2^ + 0.000017 * Nc^3^) compared to a linear model which can account for only 18% of the variability of Fop by Nc (*R* ^2^ = 0.182, p < 0.001). On the other hand in all the other four pathways and the whole genome there is a clear linear relationship between the two variables (4b to 4e). This is a clear deviation in trend compared to what we have observed in the other pathway datasets.

We calculated a correlation between Nc and Fop to check the possible relation of codon usage bias and the potential expression level. Spearman’s rank correlation (*ρ*) demonstrated a significant anti-correlation (p<0.001) between Nc and Fop across the pathways and the genome, where *ρ*_[Genome]_ = -0.69; *ρ*_[AAM]_ = -0.77; *ρ*_[CM]_ = -0.80; *ρ*_[EPC]_ = -0.76; *ρ*_[NM]_ = -0.78 and *ρ*_[Tr]_ = -0.73. Here a striking feature that we detected is the non-linear relationship of the Nc and Fop values of the amino acid metabolism pathway gene sequences (Fig. 5a). A cubic regression curve describes best the Fop variability (*R* ^2^ = 0.714, p < 0.001), accounting for about 71% variability, using Nc compared to a linear model that can account for only 18% of the variability of Fop by Nc (*R* ^2^ = 0.182, p < 0.001). This is an exceptional trend, since in all the other four pathways and the whole genome, a clear linear relationship between Nc and Fop exist (Fig. 5b to 5e).

### Codon adaptation index and expression level

To have a better understanding of the role of codon usage pattern on the potential expression level of a gene and to detect the variation in gene expression level among the different biological pathways, we calculated the codon adaptation index or CAI for all the gene sequences including the whole genome and the individual cellular pathways (S2 Table). A one way analysis of variance (ANOVA) demonstrated statistically significant inter-pathway and whole genome level difference in CAI values at p<0.001 level [F(5, 195195) = 484.78, p<0.001]. Post-facto comparisons using Dunnett’s T3 test indicated that the mean CAI value of the whole genome was significantly different than the individual cellular pathways. So, in comparison with the whole genome, the coding sequences of the vital biological pathways were found to exhibit an overall higher level of expression. In order to determine the effect of codon bias on gene expression level, a correlation between CAI and the parameters Nc, GC3, SCUO and Fop by Spearman’s correlation was performed (Table 2). A universal feature displayed by all these datasets seemed to be a significant positive correlation between CAI and Fop. This positive correlation indicates the presence of optimal codons for better translational accuracy.

**Table 2:** The degree of correlation estimated by Spearman’s rank-order between CAI and the different codon usage bias indicators to determine the effect of codon bias on gene expression level.

	CAI—GC3 (*ρ*)	CAI—SCUO (*ρ*)	CAI—Nc (*ρ*)	CAI—Fop (*ρ*)
	*p*< 0.0001	*p*< 0.0001	*p*< 0.0001	*p*< 0.0001
**Genome**	0.0478	0.175	-0.241	0.48
**AAM**	0.087	0.227	-0.186	0.414
**CM**	Not significant	0.201	-0.21	0.394
**EPC**	0.067	0.227	-0.244	0.417
**NM**	0.114	0.27	-0.26	0.418
**Tr**	-0.0948	0.213	-0.235	0.426

### Multiple regressions in determining the effect of codon bias on the gene expression level

In order to explain the significant overlap between the gene expression level and codon utilization bias, a hierarchical linear model was attempted. The relation between CAI and the other parameters allows one to determine and to predict the proportion of variation in expression levels using the standard codon usage parameters used in this study. We have undertaken sequential step wise multiple linear regressions incorporating the independent variables Nc, GC3, SCUO and Fop to determine the CAI. For each of the housekeeping functions, we had two groups, based on their (i) physiological affinity for oxygen requirement or tolerance and (ii) taxonomic affinities. The first group was further sub-divided into anaerobes and aerobes and the second group was sub-divided based on such taxonomic criteria as Archaeoglobi, Halobacteria, Methane producers, Thermococci and Thermoprotei. Thus we were left with seven different models for analyzing and comparing the affinities among the individuals belonging to these seven distinct groups to ascertain a specific codon usage design for controlling expression levels. Stepwise hierarchical linear regression was performed on all the seven models and a pathway wise analysis of the linear regression models reveals the following features:

1. **Amino acid metabolism system**—Fop could significantly predict CAI, F (1, 10094) = 5669.095, p<0.0001 and Fop accounted for 36% of the explained variability in CAI. Sequential multiple regressions were run to determine if the addition of Nc and SCUO improved the prediction of CAI over and above the parameter Fop. The full model of Fop and Nc to predict CAI was statistically significant with adjusted *R* ^2^ = 0.517, F (2, 10093) = 5399.42, p<0.0001. This full model thus explained a greater amount of the variability (51%) in expression level than the initial model.
2. **Carbohydrate metabolism system**—in this system, Fop accounted for 20% of the explained variability in CAI, which is statistically significant at p<0.0001 level, F (1, 5139) = 1349.60. The addition of Nc alone improved the prediction of CAI over and above Fop with adjusted *R* ^2^ = 0.24, F (2, 5138) = 811.39, p<0.0001.
3. **Energy processing and conversion system**—A linear regression using Fop as independent variable predicted CAI, F (1, 9536) = 2712.816 at p<0.0001 level and accounted for 22% of the explained variability in CAI. Collinear nature of Fop, Nc and SCUO was detected in this pathway system and hence both were dropped as predictors of CAI.
4. **Nucleotide metabolism and transport system—**about 23% of the explained variability in CAI by Fop could be accounted for in nucleotide metabolism pathway. The addition of Nc marginally improved the prediction of CAI over and above Fop with adjusted *R* ^2^ = 0.243, F (2, 3867) = 622.14, p<0.0001.
5. **Transcription system**—in this system, Fop accounted for 22.8% of the explained variability in CAI, which is statistically significant at p<0.0001 level, F (1, 5472) = 1618.62. The addition of Nc marginally improved the prediction of CAI over and above Fop. The full model of Fop and Nc to predict CAI was statistically significant with adjusted *R* ^2^ = 0.237, F (2, 5471) = 852.03, p<0.0001. The addition of Nc, a prominent indicator of CUB was unable to account for the variability in expression levels or CAI in the transcription pathway system in comparison with other pathway systems as considered by us. It appears that the expression of the genes coding for the basal transcription factors, and DNA directed RNA polymerase subunits of the transcription system is highly species specific and does not depend entirely on codon usage bias. The archaeal genome has been regarded as a mosaic of eubacterial and eukaryotic components with HGT as an underlying factor [47], where housekeeping functions are more prone to transfer compared to information processing systems [48]. For each pathway system, partial regression plots with CAI as the dependent variable and histograms showing frequency of regression standardized residual is illustrated in supplementary figures (S1 Fig. to S5 Fig.). The general linear models in all the aforementioned pathways were significantly improved with the categorization of organisms based on their physiological affinity, i.e., O_2_-tolerance and taxonomic similarities.

### Clustering based on oxygen affinity

For anaerobic archaea species, Fop and Nc explained a greater amount of variability in CAI levels which is evident from their increased adjusted *R* ^2^ values (in the range of 0.60 to 0.76 at p<0.001 level) for the amino acid metabolism and nucleotide metabolism pathways respectively. This feature might be a pointer to the fact that the anaerobes may have little choice left for their ORF structuring using codons which is a primary consequence of purifying selection they have been subjected to. It is seen that the physiological affinity of oxygen intolerance within organisms like species of *Archaeoglobus*, *Desulfurococcus*, *Ferroglobus*, *Pyrococcus*, the methanogens and many others, is a predominant factor in determining the uniformity of their metabolic processes, and this uniformity is reflected in the ORF structuring as they are constrained in structuring ORF’s with codons. This feature also indicates their conserved nature of codon usage, which is essential for efficient expression. Alternatively, it implies that the codon bias is a major determinant of gene expression regulation in anaerobic archaea. In comparison with the anaerobes, in the aerobes Fop and Nc account for less variability in CAI. The comparatively low value of adjusted *R* ^*2*^ in the aerobes (p<0.001) indicates that these archaea species have a wider choice in regulating the expression levels than could have been accounted for by codon usage bias alone. They may enjoy, so to say, a larger degree of metabolic sovereignty resulting from adaptation to the present day O_2_-rich environment which empowers the organism to generate greater amounts of energy but makes them susceptible to mutations by reactive oxygen species [49,50] and restrict them from growing in oxygen deprived habitats.

### Clustering based on taxonomic affinities

The clustering of archaea species based on their systematic affinities have a much better effect in explaining the variability in CAI levels by Fop and Nc and this suggests that taxonomically similar organisms tend to have similarities in ORF structuring pattern. The different taxonomic classes of archaea demonstrate the following traits:

a) **Archaeoglobi–**This class is represented by *Archaeoglobus profundus* Av18, *A*. *veneficus* SNP6 and *Ferroglobus placidus* AEDII12DO and about 62% (EPC) to 87.6% (CM) variability in CAI levels is explained by Fop and Nc, which is statistically significant at p<0.001 level.
b) **Halobacteriales—**This class is represented by the halophiles and here too, Fop and Nc are the major predictors of CAI excepting the transcription pathway model which is best described by Fop alone with adjusted co-efficient of determination ranging from 0.60 in transcription pathway, to 0.74 in nucleotide metabolism pathway.
c) **Methanogens—**This cluster is an amalgamation of the methane producing classes of archaea including *Methanobacteriales*, *Methanococcales*, *Methanomicrobiales*, *Methanopyrales* and *Methanosarcinales*. In these species, Fop and Nc together describes best the variation in CAI levels of carbohydrate metabolism and nucleotide metabolism pathways with adjusted *R* ^*2*^ being 0.74 (p<0.001) for both the systems.
d) **Thermococci and Thermoprotei—**In the case of *Thermococci* and *Thermoprotei*, Fop and Nc explains more than 80% of the variability in CAI levels. This is an indication that within the thermophilic and hyperthermophilic species, codon usage is a major regulator of expression levels in major pathways compared to other classes of archaea. It is only to be expected that organisms sharing similar physiological niche and taxonomic affinities would tend to display similar codon usage patterns shaped by evolution.

### Pathway-wise distribution of codon pair combinations and context bias

**Fig 6:**
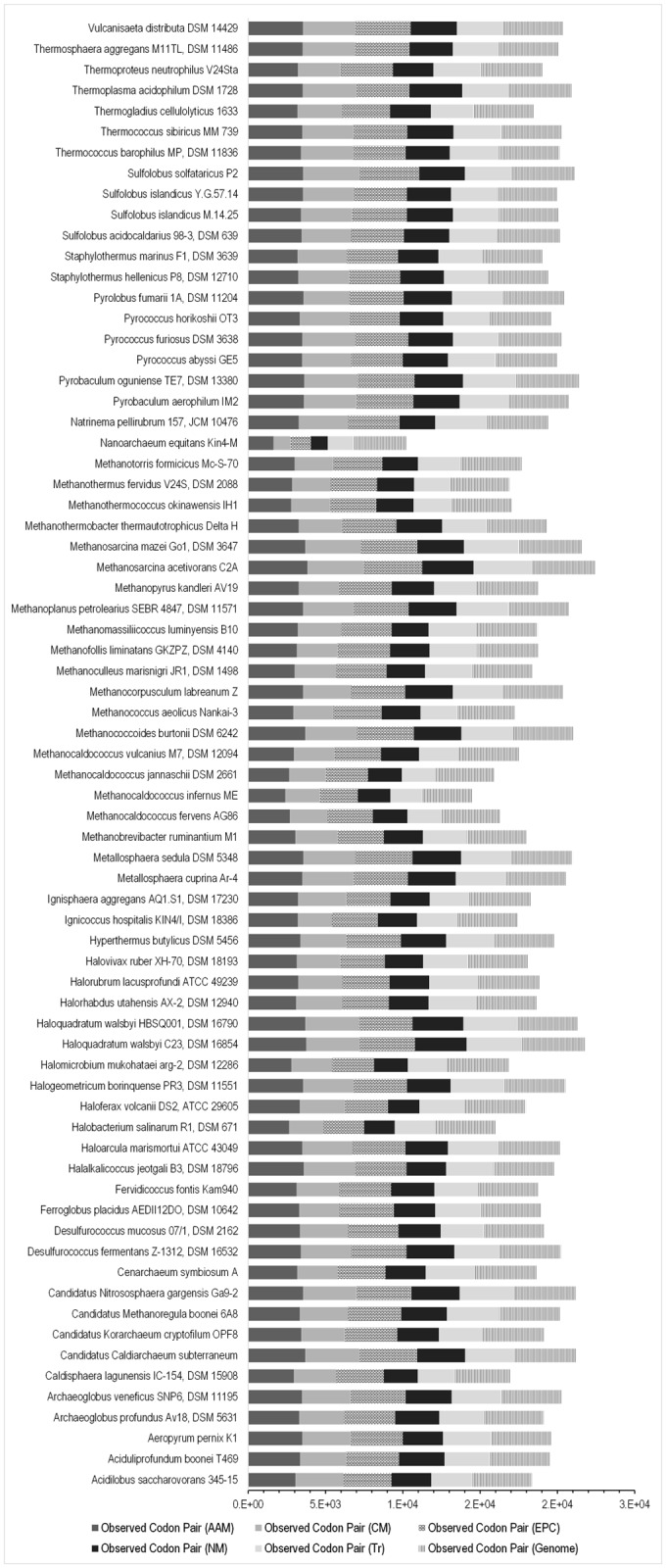
A histogram depicting the CPR for 71 archaea species included in this study. The y-axis lists the seventy one species in the order given in supplementary table (S1 Table). CP_R_ denotes the ratio of codon pair types present in the pathway to that of the whole genome and CP_R_ = 1 implying all possible genomic codon pairs is employed at the pathway level too. The CP_R_ data illustrated here indicates that there is a bias against random use of codon pairs and in this study not a single species was found to utilize all possible genomic codon pair types. In pathways like nucleotide metabolism and transcription, the CP_R_ is depressed to about 50% as seen in the halophiles *Halobacterium salinarum* R1 and *Haloferax volcanii* DS2.

To obtain an idea of the overall pattern of codon context usage at the species level and to see as to how codon-pair usage varies between among species and within species among different pathway systems, a comprehensive comparative codon pair and context analysis was carried out. We compared the number of types of codon pairs present in the whole genome with that of the codon pairs present in each pathway. We have introduced a ratio called the codon pair ratio or CP_R,_ which is tabulated in supplementary table (S3 Table). This gives a preliminary comparative idea about the magnitude of codon pair utilization bias present in the coding sequences of a specific pathway set and to what extent it resembles the whole genome usage pattern. The CP_R_ data, illustrated in Fig. 6 indicates that there is a bias against random use of codon pairs and in this study, not a single species was found to utilize all possible genomic codon pair types. In pathways like nucleotide metabolism and transcription, the CP_R_ is depressed to about 50% as seen in the halophiles *Halobacterium salinarum* R1 and *Haloferax volcanii* DS2. In *Nanoarchaeum equitans* Kin4, the CP_R_ is low and it ranges between 31% for carbohydrate metabolism and nucleotide metabolism pathways to 47% for amino acid metabolism and transcription pathways. This is lower than any organism analyzed in this study and points to the peculiar nature of *Nanoarchaeum equitans*, which lacks a host of genes necessary for carrying out vital metabolic reactions. Wilcoxon signed rank test was conducted individually for each pathway to explore significant difference in the amount of codon pair types used in a biological pathway to the amount of codon pair types employed in the whole genome. The results show a statistically significant difference in the codon pair type utilization between the whole genome and all the pathways that was considered in the present work at p<0.001 level. All the 71 species demonstrate a clear and general pattern of conserved representation of specific codon pairs in terms of pathway specific ORF structuring. A comparative account of the ten most preferred whole genome and pathway specific codon frequency for each species is given in the supplementary table (S4 Table). The frequency of each codon pair is normalized on a scale of 0 to 1, where 1 represents the maximum distribution frequency and 0 stands for the absence of that particular codon pair.

### Global codon context pattern

A genome wise comparison of codon context pattern in the 71 archaea species shows the near universal preference for CUC-UCC, CUU-GCA, AAU-CCA and CAU-CCA codon pairs. Specific codon pairs like GAA-AAA, CUC-UAC, UUC-UUC, GAG-CUU, GAA-GAG and GAA-GAA are preferred in a most of the archaea species but are suppressed in a majority of the halophilic archaea except for *Halalkalicoccus jeotgali* and *Nanoarchaeum equitans*.The codon context pairs of GCC-GUC, CAA-UCA, CAA-CUG, GCG-GAA and GUG-CUC are universally suppressed in all archaea species except in the halophiles, where these are preferred.

### Context bias in amino acid metabolism pathway

The codon pair context of AAU-CCA, CUU-GCA, CAU-CCA, GGG-CUU, AAC-CCG, AAU-CCC, CUC-UCC, CUC-UCG, AAU-CCU, GCG-AUG are somewhat universally preferred. Examples of globally avoided contexts are CGC-CAC and AGU-CGA. CGA-UCG is generally avoided in majority of the organisms except in *Candidatus Korarchaeum cryptofilum* and *Candidatus Methanoregula boonei*. Individual context maps of amino acid metabolism pathway of 71 species reveal an interesting feature where it is observed that there is an avoidance of using the codons coding for arginine i.e., CGC, CGG, and CGU in pairs. This ‘arginine pattern’ is manifested mostly by the methanogenic archaea *Methanococcus aeolicus*, *Methanocaldococcus vulcanius*, *M*. *jannaschii*, *M*. *infernus*, *M*. *fervens*, *Methanobrevibacter ruminantium*, *Methanotorris formicicus*, and also by the thermophilic *Pyrococcus abyssi*, *P*. *furiosus*, *P*. *horikoshii*, *Sulfolobus acidocaldarius*, both species of *S*. *islandicus* and *Archaeoglobus profundus*. In *Caldisphaera lagunensis*, *Fervidicoccus fontis*, *Methanocaldococcus infernus*, *M*. *jannaschii* the above mentioned codons for arginine at 5’ position or P site of ribosome are consistently avoided in tandem with other codons. In *Halobacterium salinarum*, *Halomicrobium mukohataei*, *Halorabdus utahensis*, *Halorubrum lacusprofundi*, *Haloviva xruber* and other halophiles, a selective bias towards codon pair usage have been observed. The universal ‘bad contexts’ in terms of amino acid metabolism include AAC-GAN where N is any nucleotide and the preferred ‘good contexts’ are AAC-CCN where N could be C, G or U.

### Carbohydrate metabolism pathway and context bias

Certain contexts with codons for arginine at P site of ribosome like CGA-NNN, CGG-NNN, CGU-NNN, where NNN stands for any codon other than the stop codon, is entirely avoided by all the 71 species in their carbohydrate metabolism pathway. The codon combinations which are not statistically significant are entirely shed off in carbohydrate metabolism pathway in majority of the species. A genome wide comparison of carbohydrate metabolism pathway specific context shows that in the halophiles like *Halalkalicoccus jeotgali*, *Haloarcula marismortui*, *Halobacterium salinarum*, *Halogeometricum borinquense* codon pair contexts starting with AGA-NNN are generally avoided. The halophiles show a preferrence for some contexts in their carbohydrate metabolism pathway starting with CGA-NNN, CGC-NNN, CGG-NNN, and CGU-NNN. These contexts are absent in majority of the species except some of the methanogens like *Methanomassiliicoccus luminyensis*, *Methanococcus aeolicus* and *Pyrococcus spp*. *Nanoarchaeum equitans* displays signatures of adaptation to both extreme temperature as well as symbiosis or parasitism [51–54]. It is highly specific about codon context, using only the preferred codons in the pathways. *Halobacterium salinarum* and *Ignicoccus hospitalis* also employs selected codon pairs for structuring pathways.

### Context bias in energy processing and conversion pathways

Genome wide comparison of codon context in energy processing and conversion pathway system reveals codon pairs starting with UGN-CCN **(**where N may be either of A, G, U or C**)**, that comprise the top five preferred contexts in all the species. The least preferred contexts are codon pairs beginning with AAC and are in combination with either GCG, GCU, GGA, GGC, GGG, GGU, GAC, GAA or GAG codons. CGA-NNN pairs are characteristic of halophiles, few of the methanogens and *Pyrococcus spp*. The use of codons for arginine like CGA, CGC, CGG, and CGU at 5^’^position with consecutive codons for arginine or other residues at 3^’^site appears to be forbidden in majority of the species. *Hyperthermus butylicus*, *Methanococcoides burtonii*, *Methanocorpusculum labreanum*, *Methanopyrus kandleri* and other methanogens are an exception to the general. In *Methanobrevibacter ruminantium*, and *Methanocaldococcus infernus* specific proline (CCG) and serine (UCG) codons are avoided by neighbouring 5’ codons.

### Context bias in nucleotide metabolism and transport pathways

This system depicts a codon pair context pattern where all the species are inclined towards the usage of codon pair contexts with selective constraints. The ‘arginine pattern’ observed in energy processing and conversion pathway system is clearly dominant within this pathway system and codon pairs (NNU-NNA) ending with U at the P site codon and A at the A site codon is prevalent. The halophiles, in contrast to the other pathway systems do not show affinity towards any specific codon pairs.

### Context bias in transcription machinery coding sequences

In *Methanobrevibacter ruminantium*, *Methanocaldococcus fervens*, *M*. *infernus*, *M*. *jannaschii*, *M*. *vulcanius*, *Methanococcus aeolicus* and *Methanothermus fervidus* codon pairs beginning with the specific G-ending ones of serine (UCG), threonine (ACG), proline (CCG and CCC) and alanine (GCG) at the 5’ position are entirely avoided. The ‘arginine pattern’ is visible and is consistently in line with the other major pathway systems. In terms of non-random codon pair usage the halophiles, *Halogeometricum borinquense* and both the species of *Haloquadratum walsbyi* portray the deployment of maximum codon pairs. The most preferred codon pair context in terms of transcription pathway coding sequences is UGU-CCA/U.

### Avoidance of specific tandem equal codons

Out of the 61 possible tandem equal sense codon pairs, some are universally avoided in the five vital pathways of different archaea species. This indicates that consecutive incorporation of some amino acids within the primary polypeptide chain by the ribosome is selectively avoided at the coding level. The codon pairs in context which are avoided in general among the pathways are all examples of cytosine initiated codons and include CGA-CGA, CGC-CGC, CGG-CGG. The nucleotide metabolism and transport pathways in addition to these codon pairs also avoid CAA-CAA (Glutamine), CAU-CAU (Histidine), CCA-CCA (Proline), CCC-CCC (Proline), CCU-CCU (Proline) and CUA-CUA (Leucine). The halophiles were found to specifically avoid the AGA-AGA (Arginine) pair.

### Codon-pathway interrelation in shaping codon design of pathways

We have also tried to detect whether the interaction between the codons and different pathway in an organism helps to shape up the organism’s overall codon usage design. A factorial two way analysis of variance was performed for this purpose. We detected a significant interaction between codon type and pathway on codon usage pattern of the individual pathways, *F*(252, 22400) = 1.745, *p*<0.001. A substantial difference was observed in the codon utilization ratio among the five different pathways. This is detected from the *F* values at *p*<0.001 significance level where, amino acid metabolism *F*(63, 22400) = 48.835, carbohydrate metabolism *F*(63, 22400) = 45.042, energy production and conversion *F*(63, 22400) = 4 8.284, nucleotide metabolism *F*(63, 22400) = 54.920 and transcription *F*(63, 22400) = 60.284.

The transcription pathway was found to display a significantly different codon frequency signature compared to the other pathways. The codons responsible for this variation in the transcription pathway codon frequency were AAG (Lys), AGA (Arg), AGG (Arg) and GAA (Glu) and GAG (Glu), *SE* = 0.002 at p<0.05 level.

The frequency of the lysine encoding codon AAG was also found to be important. AAG displayed a frequency which is dissimilar for the different pathways, *viz*. amino acid metabolism, carbohydrate metabolism, energy production and conversion system, transcription (*p*<0.001) and nucleotide metabolism (*p* = 0.02). AAG thus appears to have a signature frequency which is characteristic of the different pathway systems.

## Conclusion

This analysis was an attempt to explore the diversity and integrity in the codon usage design of members of the domain, in terms of core metabolic pathways and genetic information processing system. We observed that in the different members of archaea, there is a trend towards biased use of synonymous codons in the core housekeeping pathways with the Nc-plots acting as fingerprints of physiological variations prevailing in varied archaea species. HLM analyses showed that—(i) taxonomically related organisms tend to have similarities in ORF structuring pattern and (ii) aerobic archaea species resorted to a wider choice in regulating expression levels than could have been accounted for by codon usage bias alone. The latter is a consequence of their larger degree of metabolic sovereignty resulting from adaptation to the relatively ‘modern’ O_2_-rich present day environment, which endows organism to generate greater amounts of energy but restrict them from growing in oxygen deprived habitats and makes them susceptible to mutations by reactive oxygen species. Codon bias was found to be a major determinant of gene expression regulation in anaerobic archaea. The codon pair study indicated a trend towards non-random use of codon pairs within vital pathways among all species and a significant difference in the codon pair type utilization between the whole genome and all the coding sequences of the pathways was observed. Pathway specific codon context bias was found to exist among the archaea species and a codon- pathway interaction in shaping codon design of pathways was observed with the transcription pathway displaying a significantly different codon frequency signature.

## Materials and Methods

The complete genome sequences of 71 archaea species was downloaded from Integrated Microbial Genomes database [55] and GenBank [56]. The sequences coding for the enzymes and proteins of the different cellular pathways were segregated and organized employing the reference maps and species-specific pathway maps present in KEGG PATHWAY, a system information and wiring diagram database. The KEGG PATHWAY database is a collection of pathway maps integrating many entities which include nucleic acid sequences, proteins, glycans, chemical reactions, as well as disease genes and drug targets, which are stored as individual entries in the other databases of KEGG [57–59]. This yielded in addition to the whole genome dataset, five additional datasets per organism which include the amino acid metabolism system dataset, carbohydrate metabolism, energy production and conversion, nucleotide metabolism and transport and transcription pathway system dataset. We have computed the different codon usage parameters for 161,085 gene sequences representing the whole genome of the 71 archaea species involved in our study. We have segregated the gene sequences participating in the five pathway systems and they represent 10,025 gene sequences for the amino acid metabolism pathway, 5141 gene sequences for the carbohydrate metabolism pathway, 9533 gene sequences for the energy production and conversion pathways, 3870 gene sequences for nucleotide metabolism pathway and 5472 gene sequences for the transcription pathway (S2 Table).

### Analysis of codon usage bias

The effective number of codons (ENC or Nc) and GC3 were calculated using CodonW [60]. Nc is a simple measure of codon bias ranging from 20 to 61 with lower values depicting higher codon bias. GC3 represents the guanine and cytosine (G+C) content at the third position of a codon [27] and have been found to play a vital role in cell function [61,62]. It is one of the major driving force of codon usage bias [30,34]. The other codon usage parameters like SCUO and Fop was calculated utilizing the software Interactive Codon Usage Analysis 2 or ‘INCA 2’ [63]. SCUO estimates the orderliness of synonymous codon usage and codon bias based on Shannon informatics theory and the entropy theory [34]. Its value ranges from 0 to 1 with higher values indicating higher degree of information or less entropy and hence greater bias in codon choice. Fop is a codon usage measure which is the ratio of the number of optimal codons to the total number of codons and is a reference based measure where highly expressed genes are used as reference set [35]. These genes are those which encode for translation elongation factors and ribosomal proteins [64] and for each individual organism in this study, a reference family set was designed. The codon adaptation index or CAI [31] was calculated using an improved implementation by Xia [65]. The Codon Adaptation Index ranges from 0 to 1.0, with higher CAI values signifying higher degree of expressivity. The index assesses the extent to which selection has been effective in moulding the pattern of codon usage and in that respect it is useful for predicting the level of expression of a gene, [31,32,65]. CAI has been regarded as a method for predicting gene expression levels by comparative studies [66] and has also been used to assess the functional conservation of gene expression across diverse microbial species [67].

Statistical analysis of the inter-relationships among the different parameters was performed using both parametric and non-parametric correlation studies after a thorough scrutiny of the data distribution pattern. Kruskal-Wallis ANOVA on ranks along with post hoc analysis using Dunn’s procedure with a Bonferroni correction for multiple comparisons and Mann-Whitney *U* Test [68,69] was employed for detecting the distribution of Nc, SCUO and Fop between the whole genome and the individual cellular pathways. A one way analysis of variance (ANOVA) was carried out to compare the means between the whole genome and the cellular pathway datasets for detecting substantial difference, if any, in the expression level between the whole genome and the different pathways. Dunnett’s modified Tukey-Kramer multiple comparison test adjusted for unequal variances and unequal sample sizes (Dunnett’s T3) was used for post hoc analysis [70]. This is a conservative test whose family-wise error rate (FWE) does not exceed alpha and will help to detect inter-pathway variation in expression levels. To compare the coding strategies among organisms across vital pathways, hierarchical stepwise multiple linear regression was performed incorporating the independent variables Nc, GC3, SCUO and Fop to determine CAI. Here we group together all the similar pathway gene sequences into a single data set. Variance Inflation Factor or VIF was used as the parameter to ascertain the co-linearity of the variables concerned and once the co-linear variables are ascertained, they were subsequently dropped from the regression models.

### Codon pair and context analysis

The different types of codon pairs present in each particular pathway system for each individual organism was worked out along with the codon pair ratio or CP_R_. CP_R_ denotes the ratio of codon pair types present in the whole genome to that present in the pathways and CP_R_ = 1 implies that all the possible genomic codon pairs is employed at the pathway level too. This gives a preliminary comparative idea about the magnitude of codon pair utilization bias present in the coding sequences of a specific pathway set and to what extent it resembles the whole genome usage pattern. An important feature of primary gene structure is codon context which modulates mRNA decoding accuracy. The context analysis was carried out using the software tool Anaconda 2 [71,72] which constructs frequency table of codon-pair context and statistical analysis of these tables through ‘chi-square test of independence’ helps one to identify preferred or biased and rejected or suppressed pairs of codons in a coding sequence.

Among all the 71 species of archaea included in this study, codon pair types and their respective counts were calculated for the whole genome as well as for the five individual pathways. For 64 codons, there are 64^2^ = 4096 possible codon pair combinations, out of which 3904 pairs are possible for each coding sequence (3721 sense codon:sense codon pairs and 183 sense codon:stop codon pairs).

**Fig 7:**
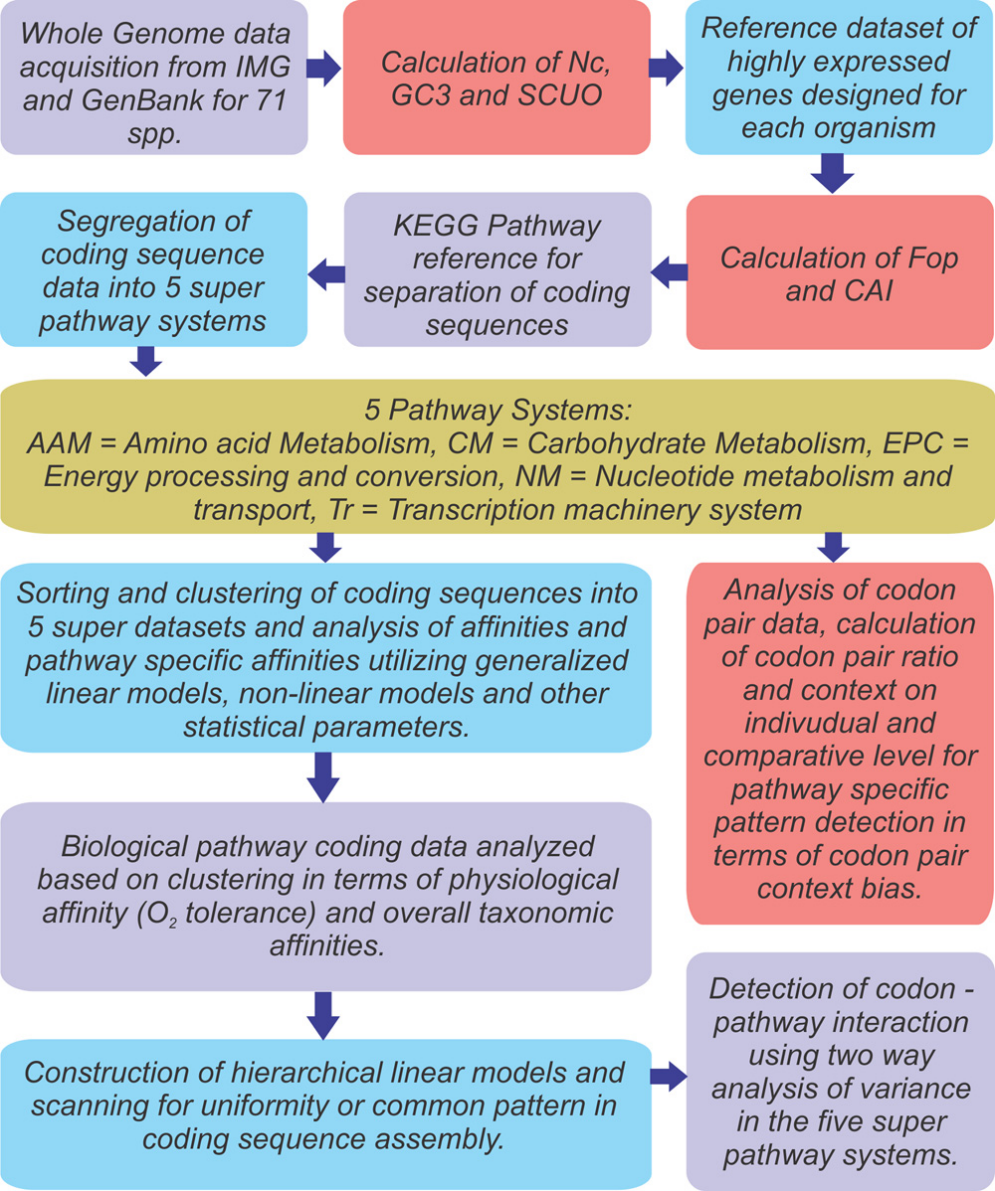
A flow chart representing the methodology and strategy applied in this study.

A factorial two way analysis of variance across 71 organisms and five pathways was carried out to detect the interaction between the 64 codons and the different pathways which ultimately reveals a unique or signature frequency of particular codon(s) to any pathway. A flow-chart highlighting the outline of the project design is given in Fig. 7.

## Supporting Information

S1 Fig Histograms depicting frequency of regression standardized residual and partial regression plots with the dependent variable CAI for the amino acid metabolism pathway (AAM). The association between CAI and the other parameters helps to predict the proportion of variance in expression levels using the standard codon usage parameters. Sequential step wise multiple linear regressions was performed to determine CAI by categorizing all the similar pathway gene sequences into a single data set and partial regression plots are constructed using the independent variables (c) Fop, (d) Nc, (e) SCUO and (f) GC3. Histograms depicting (a) frequency of regression standardized residual and (b) normal P-P plot of regression standardized residual with the dependent variable CAI is shown here for the amino acid metabolism pathway system (AAM).
(TIF)

S2 Fig Histograms depicting frequency of regression standardized residual and partial regression plots with the dependent variable CAI for the carbohydrate metabolism pathway (CM). The association between CAI and the other parameters helps to predict the proportion of variance in expression levels using the standard codon usage parameters. Sequential step wise multiple linear regressions was performed to determine CAI by categorizing all the similar pathway gene sequences into a single data set and partial regression plots are constructed using the independent variables (c) Fop, (d) Nc, (e) SCUO and (f) GC3. Histograms depicting (a) frequency of regression standardized residual and (b) normal P-P plot of regression standardized residual with the dependent variable CAI is shown here for the carbohydrate metabolism pathway system (CM).
(TIF)

S3 Fig Histograms depicting frequency of regression standardized residual and partial regression plots with the dependent variable CAI for the energy processing and conversion pathway (EPC). The association between CAI and the other parameters helps to predict the proportion of variance in expression levels using the standard codon usage parameters. Sequential step wise multiple linear regressions was performed to determine CAI by categorizing all the similar pathway gene sequences into a single data set and partial regression plots are constructed using the independent variables (c) Fop, (d) Nc, (e) SCUO and (f) GC3. Histograms depicting (a) frequency of regression standardized residual and (b) normal P-P plot of regression standardized residual with the dependent variable CAI is shown here for the energy processing and conversion pathway system (EPC).
(TIF)

S4 Fig Histograms depicting frequency of regression standardized residual and partial regression plots with the dependent variable CAI for the nucleotide metabolism and transport system (NM). The association between CAI and the other parameters helps to predict the proportion of variance in expression levels using the standard codon usage parameters. Sequential step wise multiple linear regressions was performed to determine CAI by categorizing all the similar pathway gene sequences into a single data set and partial regression plots are constructed using the independent variables (c) Fop, (d) Nc, (e) SCUO and (f) GC3. Histograms depicting (a) frequency of regression standardized residual and (b) normal P-P plot of regression standardized residual with the dependent variable CAI is shown here for the nucleotide metabolism and transport system (NM).
(TIF)

S5 Fig Histograms depicting frequency of regression standardized residual and partial regression plots with the dependent variable CAI for the transcription pathway system (Tr). The association between CAI and the other parameters helps to predict the proportion of variance in expression levels using the standard codon usage parameters. Sequential step wise multiple linear regressions was performed to determine CAI by categorizing all the similar pathway gene sequences into a single data set and partial regression plots are constructed using the independent variables (c) Fop, (d) Nc, (e) SCUO and (f) GC3. Histograms depicting (a) frequency of regression standardized residual and (b) normal P-P plot of regression standardized residual with the dependent variable CAI is shown here for the transcription pathway system (Tr).
(TIF)

S1 Table Brief details of the seventy one species of archaea scattered across the major phyla of domain archaea subjected to intra-domain analysis. (DOC)

S2 Table The codon adaptation index or CAI value range along with the average CAI value for the whole genome and the pathways of amino acid metabolism (AAM), carbohydrate metabolism (CM), energy processing and conversion pathways (EPC), nucleotide metabolism and transport system (NM) and transcription system (Tr) in the seventy one archaea species included in this study. (DOC)

S3 Table A comparative account of the codon pair ratio or CP_R_ in the pathways of amino acid metabolism (AAM), carbohydrate metabolism (CM), energy processing and conversion pathways (EPC), nucleotide metabolism and transport system (NM) and transcription system (Tr) in the seventy one archaea species included in this study. (DOC)

S4 Table A comparative account of the ten most frequently used codon pairs with their normalized frequency across the whole genome and the pathways of amino acid metabolism (AAM), carbohydrate metabolism (CM), energy processing and conversion pathways (EPC), nucleotide metabolism and transport system (NM) and transcription system (Tr) in the seventy one archaea species included in this study. (DOC)
