# Supplementary figures and images for: Deconstruction of Archaeal Genome Depict Strategic Consensus in Core Pathways Coding Sequence Assembly
Source: PLoS One. 2015 Feb 12;10(2):e0118245. doi: 10.1371/journal.pone.0118245 (PMC4326414; doi:10.1371/journal.pone.0118245)

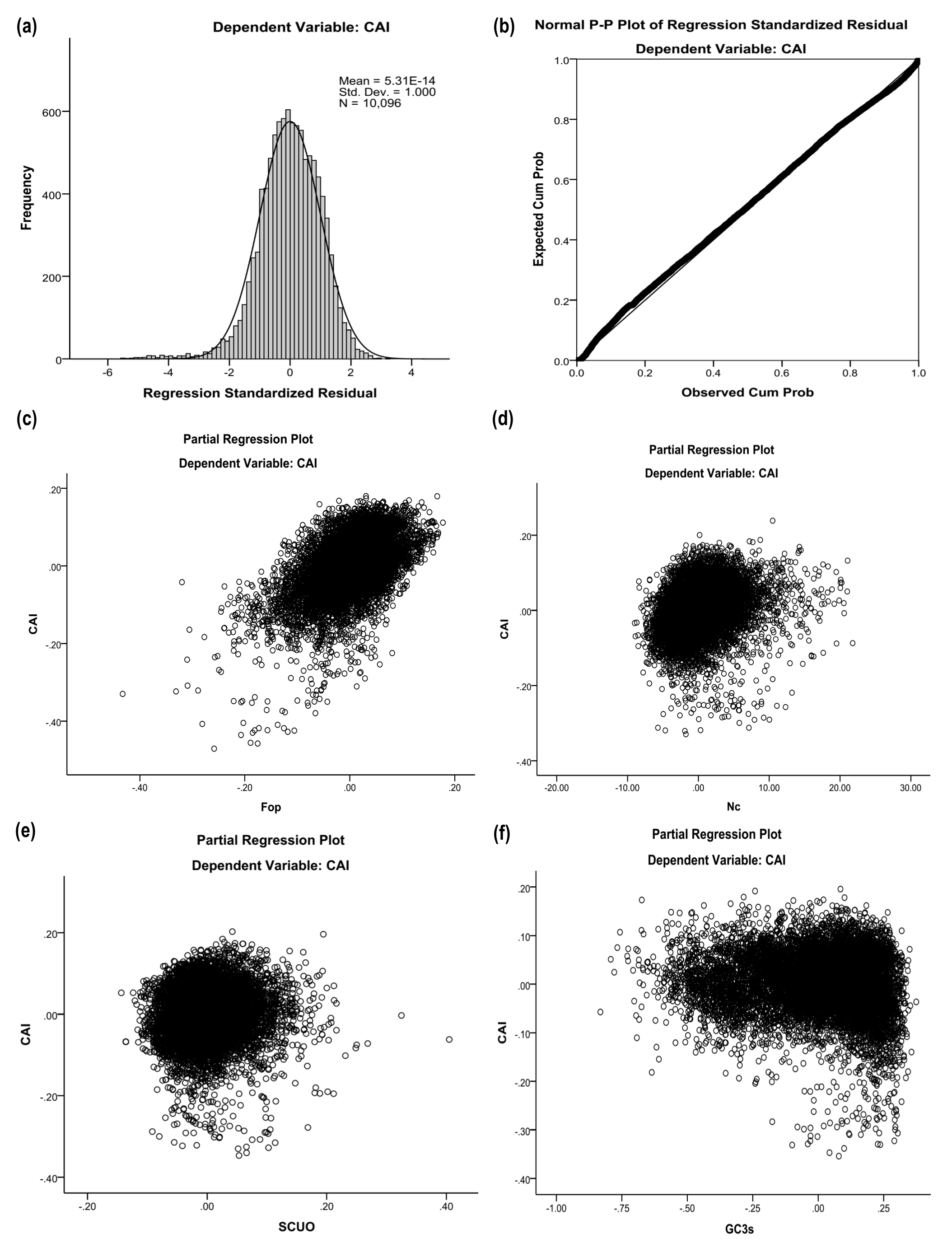

Supplement: S1 Fig — The association between CAI and the other parameters helps to predict the proportion of variance in expression levels using the standard codon usage parameters. Sequential step wise multiple linear regressions was performed to determine CAI by categorizing all the similar pathway gene sequences into a single data set and partial regression plots are constructed using the independent variables (c) Fop, (d) Nc, (e) SCUO and (f) GC3. Histograms depicting (a) frequency of regression standardized residual and (b) normal P-P plot of regression standardized residual with the dependent variable CAI is shown here for the amino acid metabolism pathway system (AAM). (TIF) [file pone.0118245.s001.tif]

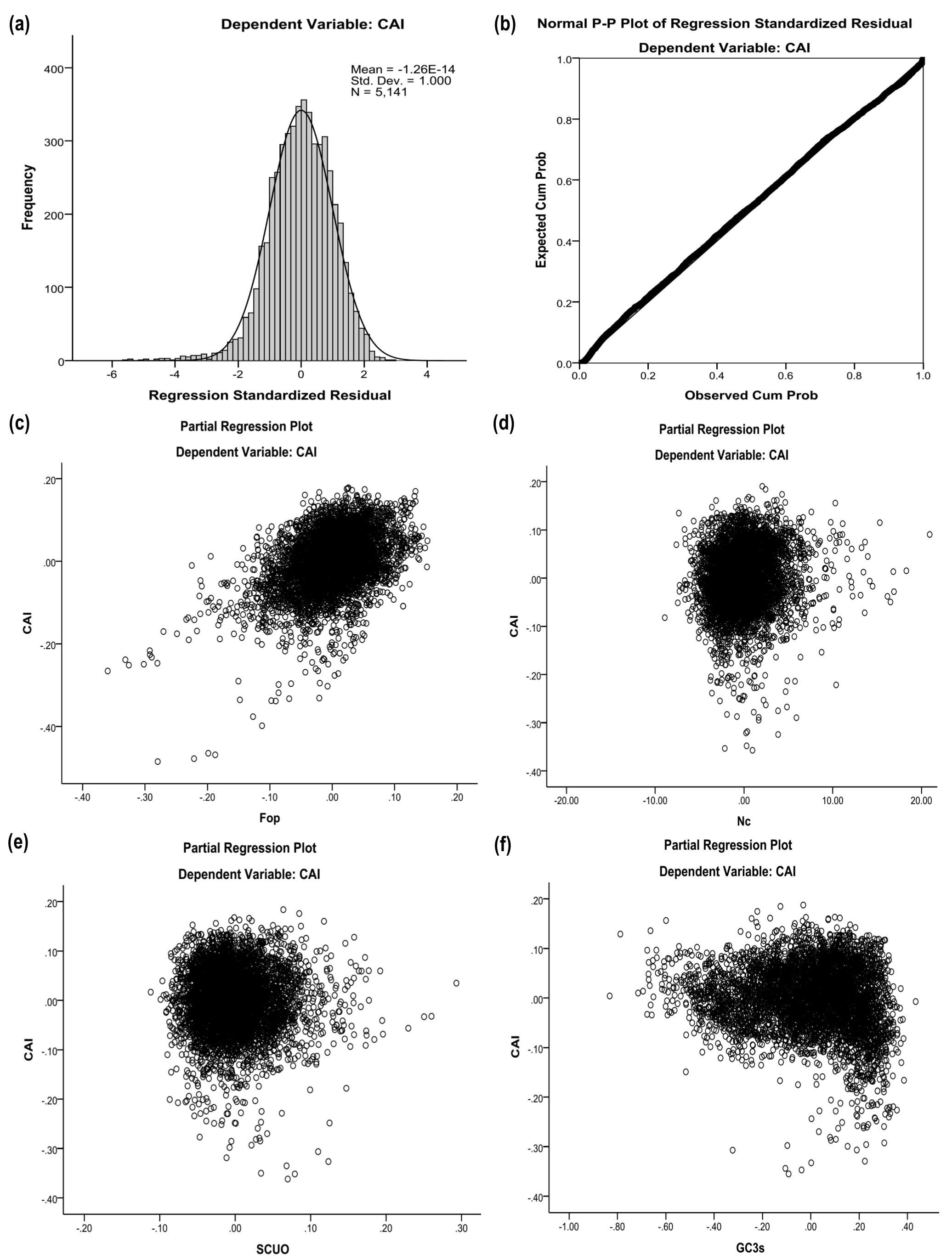

Supplement: S2 Fig — The association between CAI and the other parameters helps to predict the proportion of variance in expression levels using the standard codon usage parameters. Sequential step wise multiple linear regressions was performed to determine CAI by categorizing all the similar pathway gene sequences into a single data set and partial regression plots are constructed using the independent variables (c) Fop, (d) Nc, (e) SCUO and (f) GC3. Histograms depicting (a) frequency of regression standardized residual and (b) normal P-P plot of regression standardized residual with the dependent variable CAI is shown here for the carbohydrate metabolism pathway system (CM). (TIF) [file pone.0118245.s002.tif]

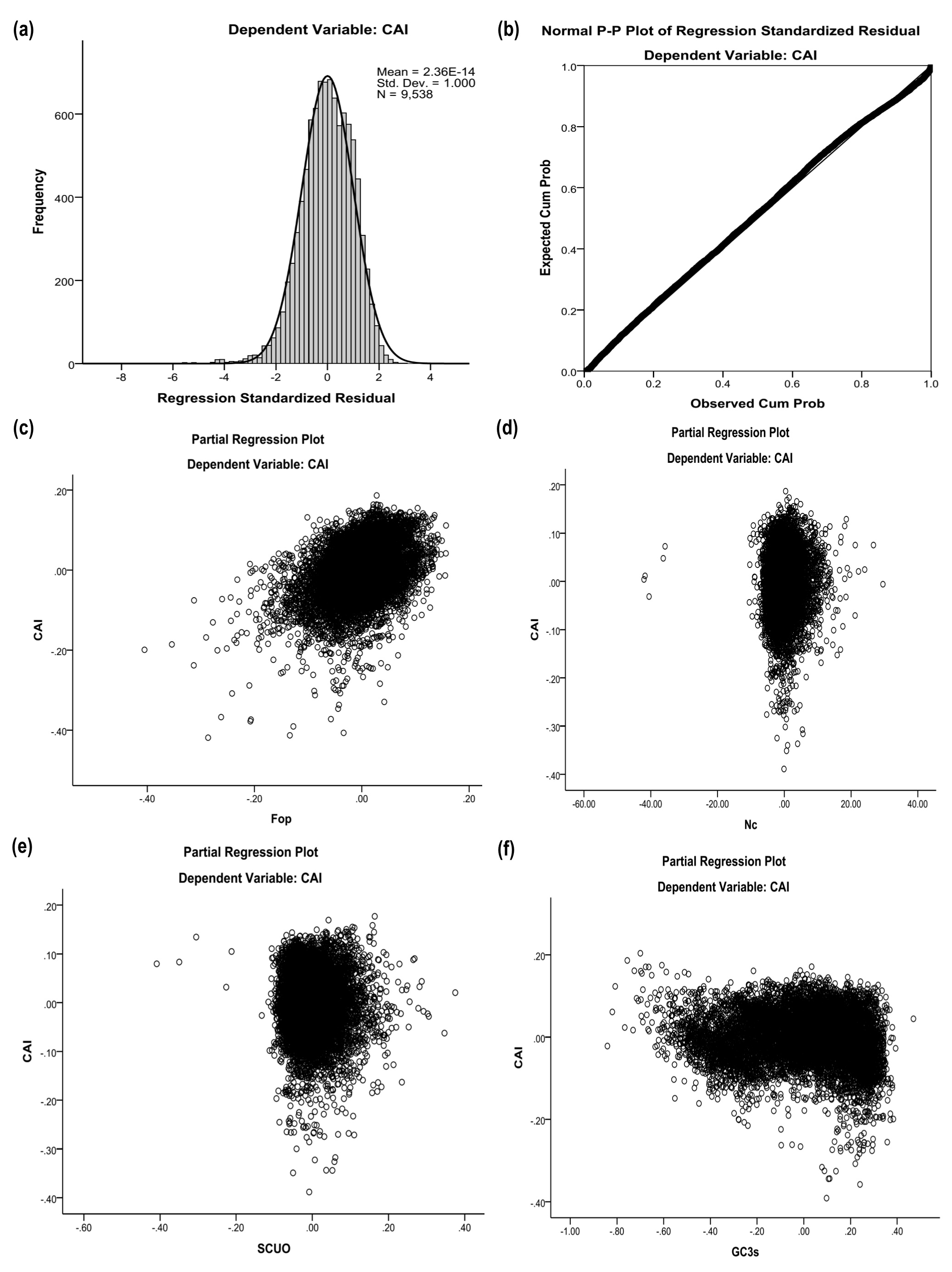

Supplement: S3 Fig — The association between CAI and the other parameters helps to predict the proportion of variance in expression levels using the standard codon usage parameters. Sequential step wise multiple linear regressions was performed to determine CAI by categorizing all the similar pathway gene sequences into a single data set and partial regression plots are constructed using the independent variables (c) Fop, (d) Nc, (e) SCUO and (f) GC3. Histograms depicting (a) frequency of regression standardized residual and (b) normal P-P plot of regression standardized residual with the dependent variable CAI is shown here for the energy processing and conversion pathway system (EPC). (TIF) [file pone.0118245.s003.tif]

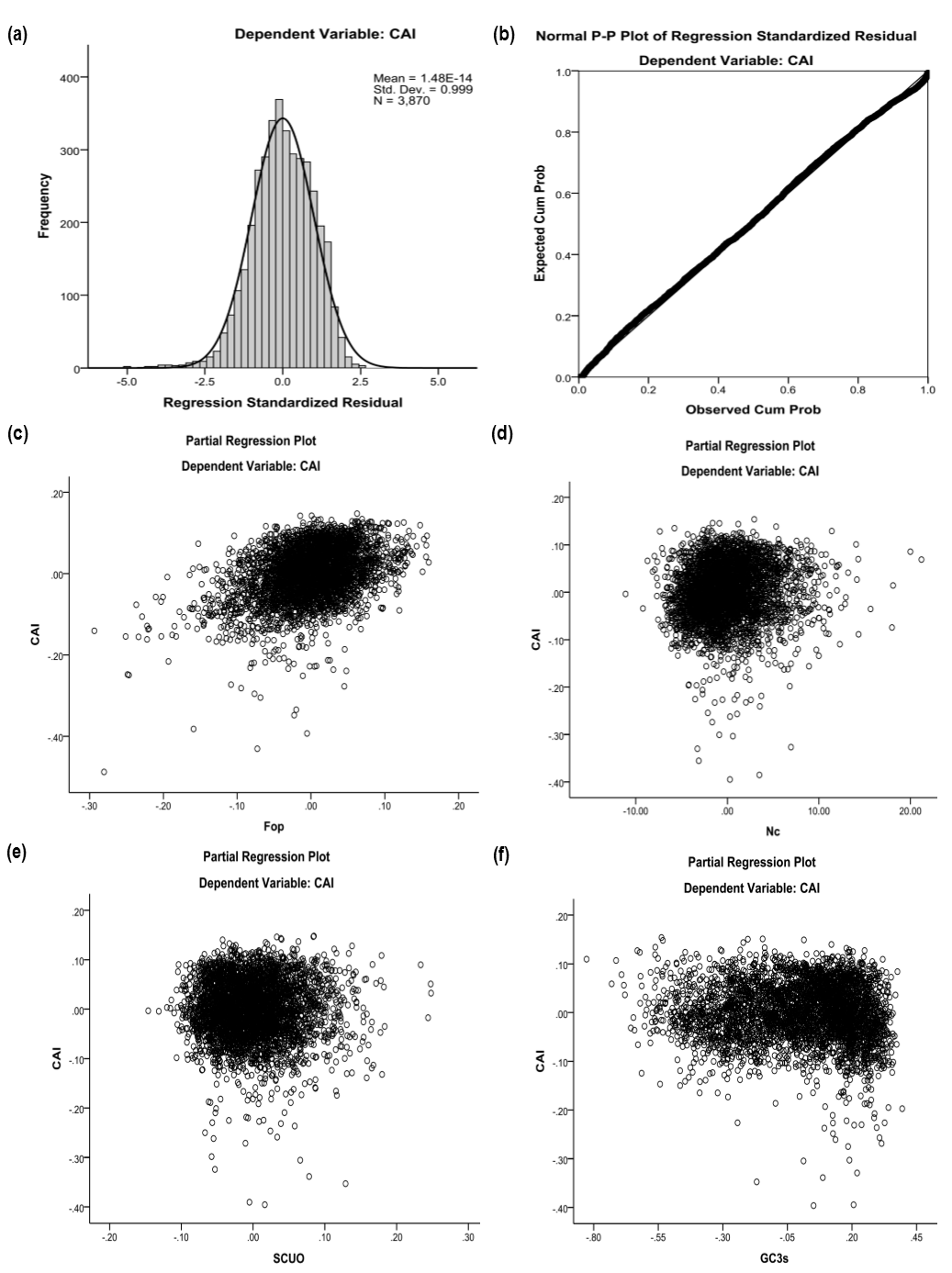

Supplement: S4 Fig — The association between CAI and the other parameters helps to predict the proportion of variance in expression levels using the standard codon usage parameters. Sequential step wise multiple linear regressions was performed to determine CAI by categorizing all the similar pathway gene sequences into a single data set and partial regression plots are constructed using the independent variables (c) Fop, (d) Nc, (e) SCUO and (f) GC3. Histograms depicting (a) frequency of regression standardized residual and (b) normal P-P plot of regression standardized residual with the dependent variable CAI is shown here for the nucleotide metabolism and transport system (NM). (TIF) [file pone.0118245.s004.tif]

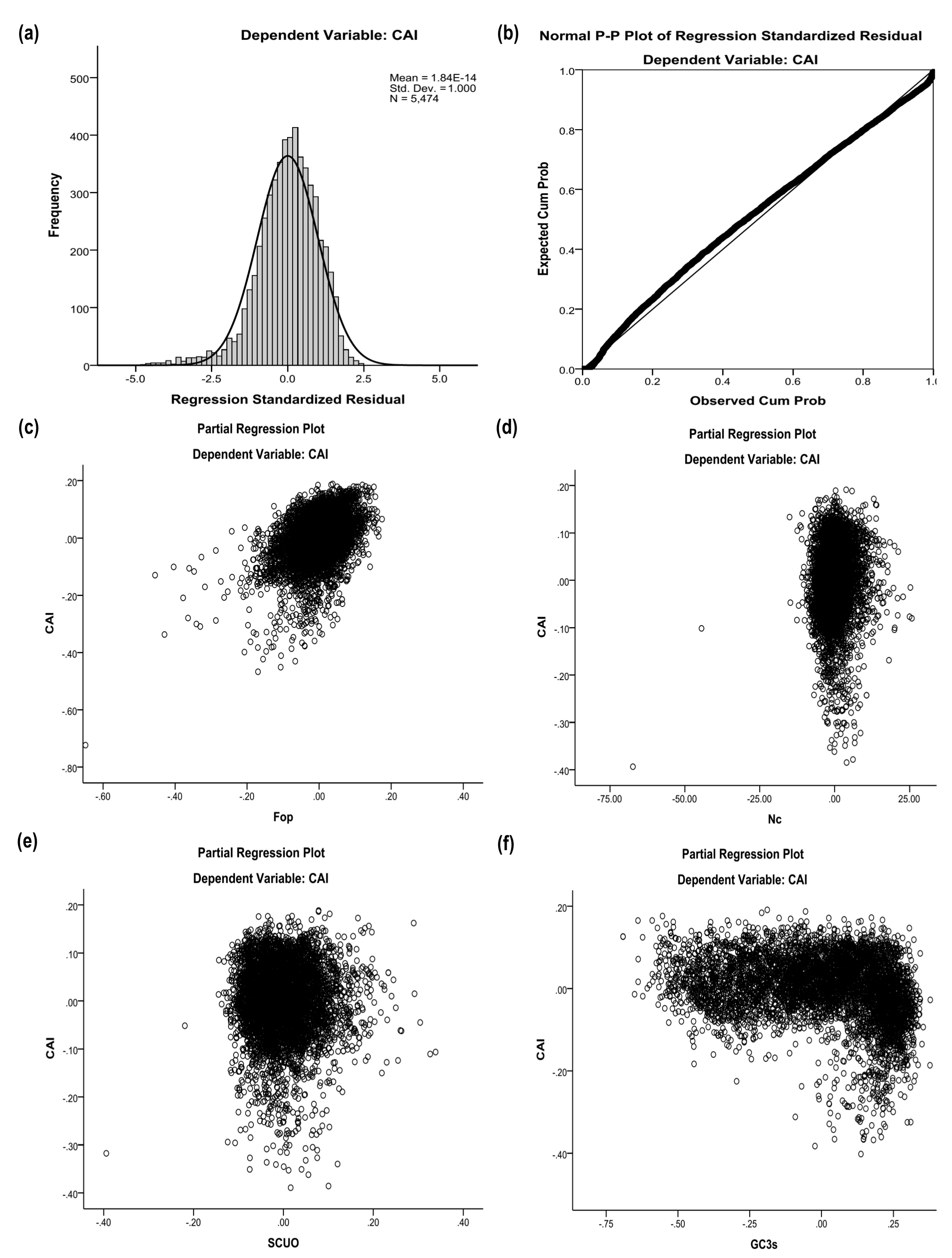

Supplement: S5 Fig — The association between CAI and the other parameters helps to predict the proportion of variance in expression levels using the standard codon usage parameters. Sequential step wise multiple linear regressions was performed to determine CAI by categorizing all the similar pathway gene sequences into a single data set and partial regression plots are constructed using the independent variables (c) Fop, (d) Nc, (e) SCUO and (f) GC3. Histograms depicting (a) frequency of regression standardized residual and (b) normal P-P plot of regression standardized residual with the dependent variable CAI is shown here for the transcription pathway system (Tr). (TIF) [file pone.0118245.s005.tif]
