# Supplementary material for: Deconstruction of Archaeal Genome Depict Strategic Consensus in Core Pathways Coding Sequence Assembly
Source: PLoS One. 2015 Feb 12;10(2):e0118245. doi: 10.1371/journal.pone.0118245 (PMC4326414; doi:10.1371/journal.pone.0118245)
Supplement: S1 Table — (DOC) [file pone.0118245.s006.doc]

**Table S1.** Brief details of the seventy one species of archaea scattered across the major phyla of domain archaea subjected to intra-domain analysis

| **ID** | **Genome Name** | **NCBI RefSeq Id** | **Phylum** | **Oxygen Requirement** | **Temperature Range** | **Genome Size** |
| --- | --- | --- | --- | --- | --- | --- |
| 1 | *Acidilobussaccharovorans 345-15* | NC_014374.1 | Crenarchaeota | Anaerobe | Hyperthermophile | 1496453 |
| 2 | *Aciduliprofundumboonei T469* | NC_013926.1 | Euryarchaeota | Facultative | Thermophile | 1486778 |
| 3 | *Aeropyrumpernix K1* | NC_000854.2 | Crenarchaeota | Aerobe | Hyperthermophile | 1669695 |
| 4 | *Archaeoglobusprofundus Av18, DSM 5631* | NC_013741.1 | Euryarchaeota | Anaerobe | Hyperthermophile | 1563423 |
| 5 | *Archaeoglobusveneficus SNP6, DSM 11195* | NC_015320.1 | Euryarchaeota | Anaerobe | Hyperthermophile | 1901943 |
| 6 | *Caldisphaeralagunensis IC-154, DSM 15908* | NC_019791.1 | Crenarchaeota | Anaerobe | Hyperthermophile | 1546846 |
| 7 | *CandidatusCaldiarchaeumsubterraneum* | BA000048.1 | Thaumarchaeota |  | Thermophile | 1680938 |
| 8 | *CandidatusKorarchaeumcryptofilum OPF8* | NC_010482.1 | Korarchaeota |  | Mesophile | 1590757 |
| 9 | *CandidatusMethanoregulaboonei 6A8* | NC_009712.1 | Euryarchaeota | Anaerobe | Mesophile | 2542943 |
| 10 | *CandidatusNitrososphaeragargensis Ga9-2* | NC_018719.1 | Thaumarchaeota | Aerobe | Thermophile | 2833736 |
| 11 | *Cenarchaeumsymbiosum A* | NC_014820.1 | Thaumarchaeota | Aerobe | Psychrophile | 2045086 |
| 12 | *Desulfurococcusfermentans Z-1312, DSM 16532* | NC_018001.1 | Crenarchaeota | Anaerobe | Hyperthermophile | 1384116 |
| 13 | *Desulfurococcusmucosus 07/1, DSM 2162* | NC_014961.1 | Crenarchaeota | Anaerobe | Hyperthermophile | 1314639 |
| 14 | *Ferroglobusplacidus AEDII12DO, DSM 10642* | NC_013849.1 | Euryarchaeota | Anaerobe | Hyperthermophile | 2196266 |
| 15 | *Fervidicoccusfontis Kam940* | NC_017461.1 | Crenarchaeota | Obligate anaerobe | Thermophile | 1319206 |
| 16 | *Halalkalicoccusjeotgali B3, DSM 18796* | NC_014297.1 | Euryarchaeota |  | Mesophile | 3698650 |
| 17 | *Haloarculamarismortui ATCC 43049* | NC_006396.1, NC_006397.1 | Euryarchaeota | Aerobe | Mesophile | 4274642 |
| 18 | *Halobacteriumsalinarum R1, DSM 671* | NC_010364.1 | Euryarchaeota | Obligate aerobe | Thermophile | 2668776 |
| 19 | *Haloferaxvolcanii DS2, ATCC 29605* | NC_013967.1 | Euryarchaeota | Aerobe | Mesophile | 4012900 |
| 20 | *Halogeometricumborinquense PR3, DSM 11551* | NC_014729.1 | Euryarchaeota | Aerobe | Mesophile | 3944467 |
| 21 | *Halomicrobiummukohataei arg-2, DSM 12286* | NC_013202.1 | Euryarchaeota | Facultative | Mesophile | 3332349 |
| 22 | *Haloquadratumwalsbyi C23, DSM 16854* | NC_017459.1 | Euryarchaeota | Aerobe | Mesophile | 3260476 |
| 23 | *Haloquadratumwalsbyi HBSQ001, DSM 16790* | NC_008212.1 | Euryarchaeota |  | Mesophile | 3179361 |
| 24 | *Halorhabdusutahensis AX-2, DSM 12940* | NC_013158.1 | Euryarchaeota | Aerobe | Mesophile | 3116795 |
| 25 | *Halorubrumlacusprofundi ATCC 49239* | NC_012029.1, NC_012028.1 | Euryarchaeota | Aerobe | Psychrophile | 3692576 |
| 26 | *Halovivaxruber XH-70, DSM 18193* | NC_019964.1 | Euryarchaeota | Aerobe | Mesophile | 3223876 |
| 27 | *Hyperthermusbutylicus DSM 5456* | NC_008818.1 | Crenarchaeota | Anaerobe | Hyperthermophile | 1667163 |
| 28 | *Ignicoccushospitalis KIN4/I, DSM 18386* | NC_009776.1 | Crenarchaeota | Anaerobe | Hyperthermophile | 1297538 |
| 29 | *Ignisphaeraaggregans AQ1.S1, DSM 17230* | NC_014471.1 | Crenarchaeota | Obligate anaerobe | Hyperthermophile | 1875953 |
| 30 | *Metallosphaeracuprina Ar-4* | NC_015435.1 | Crenarchaeota | Facultative | Thermophile | 1840348 |
| 31 | *Metallosphaerasedula DSM 5348* | NC_009440.1 | Crenarchaeota | Aerobe | Thermophile | 2191517 |
| 32 | *Methanobrevibacterruminantium M1* | NC_013790.1 | Euryarchaeota | Anaerobe | Mesophile | 2937203 |
| 33 | *Methanocaldococcusfervens AG86* | NC_013156.1 | Euryarchaeota | Anaerobe | Thermophile | 1507251 |
| 34 | *Methanocaldococcusinfernus ME* | NC_014122.1 | Euryarchaeota | Anaerobe | Thermophile | 1328194 |
| 35 | *Methanocaldococcusjannaschii DSM 2661* | NC_000909.1 | Euryarchaeota | Obligate anaerobe | Hyperthermophile | 1739927 |
| 36 | *Methanocaldococcusvulcanius M7, DSM 12094* | NC_013407.1 | Euryarchaeota | Anaerobe | Thermophile | 1761737 |
| 37 | *Methanococcoidesburtonii DSM 6242* | NC_007955.1 | Euryarchaeota | Anaerobe | Psychrophile | 2575032 |
| 38 | *Methanococcusaeolicus Nankai-3* | NC_009635.1 | Euryarchaeota | Anaerobe | Mesophile | 1569500 |
| 39 | *Methanocorpusculumlabreanum Z* | NC_008942.1 | Euryarchaeota | Anaerobe | Mesophile | 1804962 |
| 40 | *Methanoculleusmarisnigri JR1, DSM 1498* | NC_009051.1 | Euryarchaeota | Anaerobe | Mesophile | 2478101 |
| 41 | *Methanofollisliminatans GKZPZ, DSM 4140* | NZ_CM001555.1 | Euryarchaeota | Anaerobe | Mesophile | 2475100 |
| 42 | *Methanomassiliicoccusluminyensis B10* |  | Euryarchaeota |  |  | 2620233 |
| 43 | *Methanoplanuspetrolearius SEBR 4847, DSM 11571* | NC_014507.1 | Euryarchaeota | Obligate anaerobe | Mesophile | 2843290 |
| 44 | *Methanopyruskandleri AV19* | NC_003551.1 | Euryarchaeota | Anaerobe | Hyperthermophile | 1694969 |
| 45 | *Methanosarcinaacetivorans C2A* | NC_003552.1 | Euryarchaeota | Anaerobe | Mesophile | 5751492 |
| 46 | *Methanosarcinamazei Go1, DSM 3647* | NC_003901.1 | Euryarchaeota | Anaerobe | Mesophile | 4096345 |
| 47 | *Methanothermobacterthermautotrophicus Delta H* | NC_000916.1 | Euryarchaeota | Obligate anaerobe | Thermophile | 1751377 |
| 48 | *Methanothermococcusokinawensis IH1* | NC_015636.1 | Euryarchaeota | Anaerobe | Thermophile | 1677455 |
| 49 | *Methanothermusfervidus V24S, DSM 2088* | NC_014658.1 | Euryarchaeota | Obligate anaerobe | Hyperthermophile | 1243342 |
| 50 | *Methanotorrisformicicus Mc-S-70* | NZ_AGJL0.1 | Euryarchaeota | Anaerobe | Thermophile | 1945078 |
| 51 | *Nanoarchaeumequitans Kin4-M* | NC_005213.1 | Nanoarchaeota | Anaerobe | Hyperthermophile | 490885 |
| 52 | *Natrinemapellirubrum 157, JCM 10476* | NC_019962.1 | Euryarchaeota | Obligate aerobe | Mesophile | 4354100 |
| 53 | *Pyrobaculumaerophilum IM2* | NC_003364.1 | Crenarchaeota | Facultative | Hyperthermophile | 2222430 |
| 54 | *Pyrobaculumoguniense TE7, DSM 13380* | NC_016885.1 | Crenarchaeota | Facultative | Hyperthermophile | 2452920 |
| 55 | *Pyrococcusabyssi GE5* | NC_000868.1 | Euryarchaeota | Obligate anaerobe | Hyperthermophile | 1768562 |
| 56 | *Pyrococcusfuriosus DSM 3638* | NC_003413.1 | Euryarchaeota | Obligate anaerobe | Hyperthermophile | 1908256 |
| 57 | *Pyrococcushorikoshii OT3* | NC_000961.1 | Euryarchaeota | Obligate anaerobe | Hyperthermophile | 1738505 |
| 58 | *Pyrolobusfumarii 1A, DSM 11204* | NC_015931.1 | Crenarchaeota | Aerobe | Hyperthermophile | 1843267 |
| 59 | *Staphylothermushellenicus P8, DSM 12710* | NC_014205.1 | Crenarchaeota | Anaerobe | Hyperthermophile | 1580347 |
| 60 | *Staphylothermusmarinus F1, DSM 3639* | NC_009033.1 | Crenarchaeota | Anaerobe | Hyperthermophile | 1570485 |
| 61 | *Sulfolobusacidocaldarius 98-3, DSM 639* | NC_007181.1 | Crenarchaeota | Obligate aerobe | Thermophile | 2225959 |
| 62 | *Sulfolobusislandicus M.14.25* | NC_012588.1 | Crenarchaeota | Aerobe | Hyperthermophile | 2608832 |
| 63 | *Sulfolobusislandicus Y.G.57.14* | NC_012622.1 | Crenarchaeota | Facultative | Hyperthermophile | 2702058 |
| 64 | *Sulfolobussolfataricus P2* | NC_002754.1 | Crenarchaeota | Obligate aerobe | Hyperthermophile | 2992245 |
| 65 | *Thermococcusbarophilus MP, DSM 11836* | NC_014804.1 | Euryarchaeota | Anaerobe | Hyperthermophile | 2064237 |
| 66 | *Thermococcussibiricus MM 739* | NC_012883.1 | Euryarchaeota | Anaerobe | Hyperthermophile | 1845800 |
| 67 | *Thermogladiuscellulolyticus 1633* | NC_017954.1 | Crenarchaeota | Obligate anaerobe | Hyperthermophile | 1356318 |
| 68 | *Thermoplasmaacidophilum DSM 1728* | NC_002578.1 | Euryarchaeota | Facultative | Thermophile | 1564906 |
| 69 | *Thermoproteusneutrophilus V24Sta* | NC_010525.1 | Crenarchaeota | Anaerobe | Hyperthermophile | 1769823 |
| 70 | *Thermosphaeraaggregans M11TL, DSM 11486* | NC_014160.1 | Crenarchaeota | Anaerobe | Hyperthermophile | 1316595 |
| 71 | *Vulcanisaetadistributa DSM 14429* | NC_014537.1 | Crenarchaeota | Anaerobe | Hyperthermophile | 2374137 |
