# Supplementary material for: Deconstruction of Archaeal Genome Depict Strategic Consensus in Core Pathways Coding Sequence Assembly
Source: PLoS One. 2015 Feb 12;10(2):e0118245. doi: 10.1371/journal.pone.0118245 (PMC4326414; doi:10.1371/journal.pone.0118245)
Supplement: S3 Table — (DOC) [file pone.0118245.s008.doc]

**Table S3:** A comparative account of the codon pair ratio or CP_R_ in the pathways of amino acid metabolism (AAM), carbohydrate metabolism (CM), energy processing and conversion pathways (EPC), nucleotide metabolism and transport system (NM) and transcription system (Tr) in the seventy one archaea species included in this study.

| **Organism** | **CP_R_-AAM** | **CP_R_-CM** | **CP_R_-EPC** | **CP_R_-NM** | **CP_R_-Tr** |
| --- | --- | --- | --- | --- | --- |
| Acidilobussaccharovorans 345-15 | 0.7933 | 0.8071 | 0.8034 | 0.6668 | 0.6813 |
| Aciduliprofundumboonei T469 | 0.8694 | 0.7633 | 0.8911 | 0.7600 | 0.7519 |
| Aeropyrumpernix K1 | 0.8990 | 0.8059 | 0.8666 | 0.6668 | 0.7943 |
| Archaeoglobusprofundus Av18, DSM 5631 | 0.8514 | 0.7482 | 0.8522 | 0.7371 | 0.7335 |
| Archaeoglobusveneficus SNP6, DSM 11195 | 0.8942 | 0.8085 | 0.9194 | 0.7645 | 0.8196 |
| Caldisphaeralagunensis IC-154, DSM 15908 | 0.8102 | 0.7586 | 0.8399 | 0.6037 | 0.6493 |
| CandidatusCaldiarchaeumsubterraneum | 0.9387 | 0.8908 | 0.9509 | 0.7895 | 0.8185 |
| CandidatusKorarchaeumcryptofilum OPF8 | 0.8676 | 0.7130 | 0.8651 | 0.6815 | 0.7163 |
| CandidatusMethanoregulaboonei 6A8 | 0.8476 | 0.7895 | 0.8734 | 0.7494 | 0.8603 |
| CandidatusNitrososphaeragargensis Ga9-2 | 0.9109 | 0.8791 | 0.9132 | 0.8002 | 0.9185 |
| Cenarchaeumsymbiosum A | 0.8143 | 0.6706 | 0.7889 | 0.6668 | 0.8276 |
| Desulfurococcusfermentans Z-1312, DSM 16532 | 0.8764 | 0.8391 | 0.9142 | 0.7967 | 0.7677 |
| Desulfurococcusmucosus 07/1, DSM 2162 | 0.8640 | 0.8039 | 0.8361 | 0.7042 | 0.7142 |
| Ferroglobusplacidus AEDII12DO, DSM 10642 | 0.8501 | 0.6747 | 0.9051 | 0.6907 | 0.7588 |
| Fervidicoccusfontis Kam940 | 0.8182 | 0.7240 | 0.8691 | 0.7287 | 0.7431 |
| Halalkalicoccusjeotgali B3, DSM 18796 | 0.9209 | 0.8454 | 0.8521 | 0.6566 | 0.7843 |
| Haloarculamarismortui ATCC 43049 | 0.8758 | 0.8119 | 0.8602 | 0.6874 | 0.8204 |
| Halobacteriumsalinarum R1, DSM 671 | 0.6808 | 0.5689 | 0.6806 | 0.5079 | 0.6862 |
| Haloferaxvolcanii DS2, ATCC 29605 | 0.8589 | 0.7502 | 0.7173 | 0.5197 | 0.7630 |
| Halogeometricumborinquense PR3, DSM 11551 | 0.8959 | 0.8246 | 0.8659 | 0.7127 | 0.8672 |
| Halomicrobiummukohataei arg-2, DSM 12286 | 0.7085 | 0.6700 | 0.6836 | 0.5534 | 0.6543 |
| Haloquadratumwalsbyi C23, DSM 16854 | 0.9183 | 0.8395 | 0.8823 | 0.8169 | 0.8779 |
| Haloquadratumwalsbyi HBSQ001, DSM 16790 | 0.9482 | 0.8859 | 0.8880 | 0.8464 | 0.8872 |
| Halorhabdusutahensis AX-2, DSM 12940 | 0.7976 | 0.7634 | 0.7775 | 0.6631 | 0.8027 |
| Halorubrumlacusprofundi ATCC 49239 | 0.8134 | 0.7131 | 0.7650 | 0.6446 | 0.7981 |
| Halovivaxruber XH-70, DSM 18193 | 0.8081 | 0.7243 | 0.7389 | 0.6384 | 0.7405 |
| Hyperthermusbutylicus DSM 5456 | 0.8663 | 0.7637 | 0.9030 | 0.7506 | 0.7952 |
| Ignicoccushospitalis KIN4/I, DSM 18386 | 0.8259 | 0.5728 | 0.7567 | 0.6533 | 0.6808 |
| Ignisphaeraaggregans AQ1.S1, DSM 17230 | 0.8219 | 0.7892 | 0.7311 | 0.6506 | 0.6547 |
| Metallosphaeracuprina Ar-4 | 0.9047 | 0.8515 | 0.9091 | 0.8019 | 0.8345 |
| Metallosphaerasedula DSM 5348 | 0.9167 | 0.8702 | 0.9432 | 0.8080 | 0.8481 |
| Methanobrevibacterruminantium M1 | 0.8037 | 0.7113 | 0.7929 | 0.6642 | 0.7542 |
| Methanocaldococcusfervens AG86 | 0.7132 | 0.6479 | 0.7665 | 0.5922 | 0.5774 |
| Methanocaldococcusinfernus ME | 0.7448 | 0.6854 | 0.7674 | 0.6497 | 0.6420 |
| Methanocaldococcusjannaschii DSM 2661 | 0.7089 | 0.6225 | 0.7355 | 0.5812 | 0.5942 |
| Methanocaldococcusvulcanius M7, DSM 12094 | 0.7725 | 0.6764 | 0.7945 | 0.6430 | 0.6829 |
| Methanococcoidesburtonii DSM 6242 | 0.9520 | 0.8680 | 0.9471 | 0.7929 | 0.8740 |
| Methanococcusaeolicus Nankai-3 | 0.7863 | 0.7033 | 0.8378 | 0.6739 | 0.6389 |
| Methanocorpusculumlabreanum Z | 0.9141 | 0.7991 | 0.9041 | 0.7942 | 0.8406 |
| Methanoculleusmarisnigri JR1, DSM 1498 | 0.7724 | 0.6937 | 0.8281 | 0.6403 | 0.7773 |
| Methanofollisliminatans GKZPZ, DSM 4140 | 0.7978 | 0.6579 | 0.8700 | 0.6450 | 0.7851 |
| Methanomassiliicoccusluminyensis B10 | 0.8230 | 0.7174 | 0.8373 | 0.6108 | 0.8007 |
| Methanoplanuspetrolearius SEBR 4847, DSM 11571 | 0.9104 | 0.8418 | 0.9147 | 0.7922 | 0.8598 |
| Methanopyruskandleri AV19 | 0.8292 | 0.6614 | 0.8588 | 0.6932 | 0.7018 |
| Methanosarcinaacetivorans C2A | 0.9367 | 0.8830 | 0.9309 | 0.8097 | 0.9284 |
| Methanosarcinamazei Go1, DSM 3647 | 0.9063 | 0.8837 | 0.9014 | 0.7394 | 0.8699 |
| Methanothermobacterthermautotrophicus Delta H | 0.8424 | 0.7164 | 0.9046 | 0.7611 | 0.7362 |
| Methanothermococcusokinawensis IH1 | 0.7180 | 0.6482 | 0.7763 | 0.6188 | 0.6382 |
| Methanothermusfervidus V24S, DSM 2088 | 0.7467 | 0.6480 | 0.7899 | 0.6294 | 0.6163 |
| Methanotorrisformicicus Mc-S-70 | 0.7620 | 0.6241 | 0.8100 | 0.5806 | 0.6914 |
| Nanoarchaeumequitans Kin4-M | 0.4715 | 0.3133 | 0.3820 | 0.3133 | 0.4715 |
| Natrinemapellirubrum 157, JCM 10476 | 0.8075 | 0.7887 | 0.8208 | 0.5687 | 0.8134 |
| Pyrobaculumaerophilum IM2 | 0.9236 | 0.8644 | 0.9505 | 0.7642 | 0.8180 |
| Pyrobaculumoguniense TE7, DSM 13380 | 0.8974 | 0.8489 | 0.9152 | 0.7759 | 0.8481 |
| Pyrococcusabyssi GE5 | 0.8644 | 0.7874 | 0.8248 | 0.7203 | 0.7559 |
| Pyrococcusfuriosus DSM 3638 | 0.8575 | 0.8421 | 0.8350 | 0.7079 | 0.7196 |
| Pyrococcushorikoshii OT3 | 0.8484 | 0.8163 | 0.8311 | 0.7177 | 0.7743 |
| Pyrolobusfumarii 1A, DSM 11204 | 0.9182 | 0.7654 | 0.8941 | 0.8033 | 0.8531 |
| Staphylothermushellenicus P8, DSM 12710 | 0.8330 | 0.8428 | 0.8549 | 0.7260 | 0.7453 |
| Staphylothermusmarinus F1, DSM 3639 | 0.8217 | 0.8258 | 0.8500 | 0.6733 | 0.7351 |
| Sulfolobusacidocaldarius 98-3, DSM 639 | 0.8530 | 0.7968 | 0.8424 | 0.7246 | 0.7713 |
| Sulfolobusislandicus M.14.25 | 0.8861 | 0.8511 | 0.9240 | 0.7758 | 0.7758 |
| Sulfolobusislandicus Y.G.57.14 | 0.9149 | 0.8497 | 0.8877 | 0.7419 | 0.7822 |
| Sulfolobussolfataricus P2 | 0.8680 | 0.8873 | 0.9504 | 0.7225 | 0.7396 |
| Thermococcusbarophilus MP, DSM 11836 | 0.8795 | 0.8661 | 0.8764 | 0.7347 | 0.8254 |
| Thermococcussibiricus MM 739 | 0.8975 | 0.8454 | 0.8993 | 0.7671 | 0.7876 |
| Thermogladiuscellulolyticus 1633 | 0.8216 | 0.7451 | 0.7990 | 0.6804 | 0.7075 |
| Thermoplasmaacidophilum DSM 1728 | 0.8823 | 0.8589 | 0.8616 | 0.8559 | 0.7577 |
| Thermoproteusneutrophilus V24Sta | 0.8001 | 0.6786 | 0.8506 | 0.6530 | 0.7583 |
| Thermosphaeraaggregans M11TL, DSM 11486 | 0.9050 | 0.8613 | 0.9119 | 0.7203 | 0.7504 |
| Vulcanisaetadistributa DSM 14429 | 0.9075 | 0.8642 | 0.9206 | 0.7668 | 0.7635 |
