# Supplementary material for: Deconstruction of Archaeal Genome Depict Strategic Consensus in Core Pathways Coding Sequence Assembly
Source: PLoS One. 2015 Feb 12;10(2):e0118245. doi: 10.1371/journal.pone.0118245 (PMC4326414; doi:10.1371/journal.pone.0118245)
Supplement: S4 Table — (DOC) [file pone.0118245.s009.doc]

**Table S4:** A comparative account of the ten most frequently used codon pairs with their normalized frequency across the whole genome and the pathways of amino acid metabolism (AAM), carbohydrate metabolism (CM), energy processing and conversion pathways (EPC), nucleotide metabolism and transport system (NM) and transcription system (Tr) in the seventy one archaea species included in this study.

| **ID** | **Organism** | **Pathway** | **CODON PAIRS** | | | | | | | | | |
| --- | --- | --- | --- | --- | --- | --- | --- | --- | --- | --- | --- | --- |
|  |  |  | **1** | **2** | **3** | **4** | **5** | **6** | **7** | **8** | **9** | **10** |
| 1 | Acidilobussaccharovorans 345-15 | AAM | GAG-GCC | GCC-AGG | GAG-GAG | GCC-CUG | CUG-GCC | GUG-GCC | GCC-AUA | GGC-CUC | AGG-GAG | CUC-AGG |
|  |  |  | 1 | 0.8314 | 0.77907 | 0.76744 | 0.74419 | 0.72093 | 0.72093 | 0.70349 | 0.68605 | 0.65116 |
|  |  | CM | GAG-GCC | AGG-GAG | GAG-GAG | CUC-AGG | GCC-AUA | GCC-AGG | GGC-CUC | GAG-GGC | GCC-CUG | CUG-GCC |
|  |  |  | 1 | 0.90909 | 0.83117 | 0.77922 | 0.74675 | 0.74026 | 0.66234 | 0.62987 | 0.62338 | 0.61688 |
|  |  | EPC | GAG-GAG | GAG-GCC | AGG-GAG | GCC-AUA | GAG-AGG | AAG-GAG | AGG-AGG | GCC-AGG | AUA-GAG | AGG-GCC |
|  |  |  | 1 | 0.925 | 0.835 | 0.735 | 0.68 | 0.68 | 0.665 | 0.625 | 0.625 | 0.61 |
|  |  | NM | GAG-GCC | GAG-GAG | AGG-GAG | GAG-GGC | GAG-AGG | AGG-GCC | AGG-AGG | GAG-GUC | AUA-AGG | AGG-GAC |
|  |  |  | 1 | 0.96154 | 0.76923 | 0.73077 | 0.73077 | 0.73077 | 0.71154 | 0.67308 | 0.67308 | 0.67308 |
|  |  | Tr | GAG-GAG | GAG-GCC | AUA-GAG | AGG-GAG | GAG-AGG | GGC-AAG | AGG-AGG | GAG-CUC | GAG-AAG | AGG-GAC |
|  |  |  | 1 | 0.74026 | 0.71429 | 0.66234 | 0.64935 | 0.61039 | 0.5974 | 0.55844 | 0.55844 | 0.55844 |
|  |  | WG | GAG-GAG | GAG-GCC | AGG-GAG | AGG-AGG | AGG-GCC | GCC-AGG | GAG-AGG | CUC-AGG | GAG-GGC | GAG-CUC |
|  |  |  | 1 | 0.99896 | 0.85893 | 0.79021 | 0.72566 | 0.7189 | 0.69599 | 0.69547 | 0.66736 | 0.64966 |
| 2 | Aciduliprofundumboonei T469 | AAM | AUA-AAA | GAU-AUA | AAA-GAG | AUA-AUA | GAU-GAG | GAA-GAG | AAA-AUA | GAU-GAA | GAG-GAU | GAG-AUA |
|  |  |  | 1 | 0.97895 | 0.96842 | 0.95789 | 0.88421 | 0.88421 | 0.86316 | 0.82105 | 0.82105 | 0.82105 |
|  |  | CM | AUA-AAA | GAA-AAA | UUU-GGA | GGA-AUA | GCA-AUA | AAA-GAG | GCA-AAG | GAU-AUA | AUA-UUU | AAA-AUA |
|  |  |  | 1 | 0.93617 | 0.89362 | 0.89362 | 0.89362 | 0.85106 | 0.82979 | 0.82979 | 0.82979 | 0.82979 |
|  |  | EPC | AAA-GAG | GAA-GAG | AUA-AUA | GAG-AAA | GAG-GAU | GAG-GAG | AAG-AAG | GAU-GAG | GAG-AUA | AUA-AAG |
|  |  |  | 1 | 0.7931 | 0.78448 | 0.76724 | 0.75 | 0.71552 | 0.71552 | 0.7069 | 0.7069 | 0.7069 |
|  |  | NM | GAA-GAG | AUA-AAA | AAA-AUA | GAA-GAU | AAG-AUA | GAG-AUA | GAG-GAA | GAU-GAG | GAA-AAA | AAA-GAG |
|  |  |  | 1 | 0.88889 | 0.84127 | 0.77778 | 0.77778 | 0.7619 | 0.74603 | 0.73016 | 0.73016 | 0.73016 |
|  |  | Tr | AAA-GAG | AUA-AAA | AAA-GAA | AUA-AAG | AAG-GAU | AUU-GAA | AAG-AAA | GAG-AUA | GAA-GAG | AAA-AUU |
|  |  |  | 1 | 0.95082 | 0.91803 | 0.90164 | 0.85246 | 0.83607 | 0.78689 | 0.7377 | 0.7377 | 0.72131 |
|  |  | WG | AAA-GAG | AUA-AAA | AAA-AUA | GAA-GAG | AUA-AUA | GAG-AAA | AUA-AAG | AAA-GAU | GAA-AAA | GAG-AUA |
|  |  |  | 1 | 0.97762 | 0.9083 | 0.90614 | 0.85054 | 0.80217 | 0.78412 | 0.77329 | 0.77256 | 0.77184 |
| 3 | Aeropyrumpernix K1 | AAM | GAG-GAG | AGG-GAG | GAG-AGG | GUG-GAG | AGG-AGG | GAG-GCU | CUG-GAG | GAG-AAG | AGG-CUG | GAG-GCG |
|  |  |  | 1 | 0.77821 | 0.73152 | 0.71595 | 0.66537 | 0.5214 | 0.51751 | 0.51362 | 0.51362 | 0.48638 |
|  |  | CM | GAG-GAG | GAG-AGG | GAG-GCU | GAG-GCC | AGG-AGG | CUG-GAG | GCC-CUC | GUG-GAG | AGG-AUA | GGC-CUC |
|  |  |  | 1 | 0.61321 | 0.60377 | 0.60377 | 0.58491 | 0.56604 | 0.54717 | 0.5283 | 0.50943 | 0.5 |
|  |  | EPC | GAG-GAG | GAG-AAG | AGG-GAG | GAG-AGG | GAG-GCU | GUG-GAG | CUG-GAG | AGG-AGG | AGG-AUA | AGG-CUG |
|  |  |  | 1 | 0.73707 | 0.71121 | 0.68966 | 0.58621 | 0.55603 | 0.50431 | 0.50431 | 0.49569 | 0.49138 |
|  |  | NM | GUG-GAG | GAG-GAG | AGG-GAG | AGG-AGG | GAG-AGG | GAG-GCU | GUU-GAG | AGG-CUG | GCC-AGG | GAG-GUU |
|  |  |  | 1 | 0.9 | 0.85 | 0.76667 | 0.75 | 0.73333 | 0.6 | 0.56667 | 0.5 | 0.48333 |
|  |  | Tr | GAG-GAG | AGG-GAG | GAG-AUA | GAG-AAG | AGG-AGG | GAG-AGG | GAG-GCU | GAC-GAG | AAG-AGG | AUA-GAG |
|  |  |  | 1 | 0.59836 | 0.54098 | 0.54098 | 0.5082 | 0.45082 | 0.38525 | 0.38525 | 0.38525 | 0.37705 |
|  |  | WG | GAG-GAG | GAG-AGG | AGG-GAG | AGG-AGG | GAG-AAG | GUG-GAG | GAG-GCU | CUG-GAG | AGG-CUG | GAG-GCC |
|  |  |  | 1 | 0.71459 | 0.70361 | 0.69263 | 0.58924 | 0.57507 | 0.54391 | 0.50035 | 0.43839 | 0.42068 |
| 4 | Archaeoglobusprofundus Av18, DSM 5631 | AAM | GUU-GAG | AAG-GAG | GAG-AAG | GAG-CUU | GUU-GAA | AAG-GCU | GAU-GUU | AUA-AAG | AAG-AAG | AUA-GUU |
|  |  |  | 1 | 1 | 0.9115 | 0.89381 | 0.88496 | 0.82301 | 0.78761 | 0.78761 | 0.77876 | 0.76991 |
|  |  | CM | AAG-GAG | GAA-GUU | GUU-GAA | GAG-AAG | GAG-CUU | AAG-AAG | GAU-GAG | GCU-UUG | GAG-AUA | GUU-GAG |
|  |  |  | 1 | 0.95122 | 0.92683 | 0.90244 | 0.87805 | 0.87805 | 0.85366 | 0.82927 | 0.82927 | 0.80488 |
|  |  | EPC | GAG-AAG | GUU-GAA | AAG-GAG | AAG-AAG | AUA-AAG | AAG-GUU | GAA-GUU | GUU-GAG | GAA-GAG | GUU-AAG |
|  |  |  | 1 | 0.95798 | 0.86555 | 0.84874 | 0.76471 | 0.7563 | 0.73109 | 0.69748 | 0.68908 | 0.66387 |
|  |  | NM | GUU-GAA | AAG-GAG | GAG-GAG | GAA-GAG | GAG-AUA | AUA-AAG | GAA-GUU | GAG-AAG | AGA-GAG | GUU-GAG |
|  |  |  | 1 | 0.85455 | 0.83636 | 0.83636 | 0.8 | 0.78182 | 0.76364 | 0.74545 | 0.74545 | 0.72727 |
|  |  | Tr | AAG-GAG | AAG-AAG | GAG-AAG | GAA-GAG | GAA-AAG | GAG-GAG | GAG-CUU | GUU-GAG | GAU-GAG | AUA-GAG |
|  |  |  | 1 | 0.96667 | 0.93333 | 0.78333 | 0.76667 | 0.73333 | 0.71667 | 0.7 | 0.65 | 0.65 |
|  |  | WG | AAG-GAG | GAG-AAG | AAG-AAG | GUU-GAA | GAG-GAG | GAA-GUU | GUU-GAG | GAA-AAG | AAG-GUU | GAA-GAG |
|  |  |  | 1 | 0.96691 | 0.95809 | 0.92132 | 0.87868 | 0.82574 | 0.81838 | 0.79191 | 0.78015 | 0.77647 |
| 5 | Archaeoglobusveneficus SNP6, DSM 11195 | AAM | GAG-AAG | AAG-AAG | GAG-GAG | AAG-GAG | GAA-GAG | GUU-GAG | GCA-AAG | CUU-GCA | GUU-GAA | CUC-GUU |
|  |  |  | 1 | 0.98276 | 0.9569 | 0.89655 | 0.82759 | 0.81897 | 0.74138 | 0.73276 | 0.71552 | 0.71552 |
|  |  | CM | GAG-AAG | GAG-GAG | AAG-GAG | GAA-AAG | GAG-GAA | GUU-GAG | GAG-AAA | GUU-GUU | GGA-AAG | GAA-GUU |
|  |  |  | 1 | 0.79245 | 0.75472 | 0.71698 | 0.64151 | 0.62264 | 0.62264 | 0.60377 | 0.60377 | 0.60377 |
|  |  | EPC | GAG-AAG | AAG-AAG | GAG-GAG | AAG-GAG | GAA-GAG | GCA-AAG | AAG-CUC | GUU-GAG | AUA-AAG | CUU-GCA |
|  |  |  | 1 | 0.87571 | 0.85876 | 0.76836 | 0.74576 | 0.74011 | 0.74011 | 0.69492 | 0.64972 | 0.61582 |
|  |  | NM | GAG-GAG | GAG-AAG | GAA-GAG | GUU-GAA | AAG-GAG | AUA-AAG | AAG-CUC | AUC-GAG | GAG-GAA | GAG-AAA |
|  |  |  | 1 | 0.86364 | 0.74242 | 0.72727 | 0.68182 | 0.65152 | 0.60606 | 0.59091 | 0.56061 | 0.56061 |
|  |  | Tr | GAG-AAG | GAG-GAG | GAG-GAA | AAG-CUC | GAA-GAG | AUA-GAG | AAG-AAG | AAG-GAG | GAA-CUC | GAG-AUA |
|  |  |  | 1 | 0.66667 | 0.64368 | 0.63218 | 0.62069 | 0.6092 | 0.6092 | 0.58621 | 0.55172 | 0.51724 |
|  |  | WG | GAG-GAG | GAG-AAG | AAG-AAG | GAA-GAG | AAG-GAG | GAG-GAA | GAA-AAG | GAA-GAA | GUU-GAG | AAG-CUC |
|  |  |  | 1 | 1 | 0.84008 | 0.83249 | 0.80468 | 0.71365 | 0.7067 | 0.69848 | 0.67257 | 0.65866 |
| 6 | Caldisphaeralagunensis IC-154, DSM 15908 | AAM | AAA-GAA | AAA-AUA | UUA-AAU | AUA-AUA | AAU-GAA | GAA-AAA | GAA-AUA | AUA-AAA | UUA-AUA | AUA-AAU |
|  |  |  | 1 | 0.97101 | 0.94686 | 0.9372 | 0.91787 | 0.89372 | 0.86957 | 0.85024 | 0.84058 | 0.81643 |
|  |  | CM | UUA-AUA | AUA-AUA | AUA-AAA | AAA-AUA | GAA-AAA | AAA-GAA | UUA-UUA | GAA-AUA | AAU-AAA | GCA-UUA |
|  |  |  | 1 | 0.9037 | 0.88148 | 0.83704 | 0.81481 | 0.80741 | 0.79259 | 0.75556 | 0.6963 | 0.68148 |
|  |  | EPC | AAA-GAA | GAA-AAA | GAA-GAA | GAA-AUA | AAA-AUA | AUA-GAA | AUA-AUA | AUA-AAA | AAA-GAU | GCA-UUA |
|  |  |  | 1 | 0.87624 | 0.84158 | 0.74752 | 0.73762 | 0.72277 | 0.70792 | 0.70792 | 0.64356 | 0.63861 |
|  |  | NM | AAA-GAA | GAA-GAA | AUA-GAU | AUA-AAA | AAA-AUA | AUA-AUA | AUA-AAU | GAA-AAA | AAU-GAA | UUA-AAU |
|  |  |  | 1 | 0.89855 | 0.84058 | 0.84058 | 0.84058 | 0.81159 | 0.81159 | 0.78261 | 0.73913 | 0.71014 |
|  |  | Tr | GAA-AAA | AAA-GAA | GAA-AUA | GAA-GAA | AUA-GAA | AAU-AAA | AAA-AUA | AUA-AUA | GAU-GAA | GAA-AAU |
|  |  |  | 1 | 0.98901 | 0.97802 | 0.85714 | 0.78022 | 0.78022 | 0.72527 | 0.7033 | 0.68132 | 0.64835 |
|  |  | WG | AAA-GAA | AAA-AUA | GAA-AAA | AUA-AUA | AUA-AAA | GAA-GAA | GAA-AUA | UUA-AUA | AAU-AAA | AUA-AAU |
|  |  |  | 1 | 0.90533 | 0.89534 | 0.86013 | 0.82065 | 0.80542 | 0.79068 | 0.74643 | 0.745 | 0.73549 |
| 7 | CandidatusCaldiarchaeumsubterraneum | AAM | GAG-GAG | GAG-AAA | GUG-GAG | GAG-AAG | GUU-GAG | GAG-GUU | GAG-CUG | CUC-GGC | CUC-GAC | CUC-GAG |
|  |  |  | 1 | 0.96992 | 0.88722 | 0.87218 | 0.84211 | 0.83459 | 0.80451 | 0.79699 | 0.72932 | 0.71429 |
|  |  | CM | GAG-AAA | AAC-AUC | GAA-AAA | GAG-GAG | GUU-GAG | UUC-UUC | GUU-UUG | GGU-GUG | GUU-GAC | GGA-AAA |
|  |  |  | 1 | 0.94231 | 0.92308 | 0.90385 | 0.88462 | 0.86538 | 0.78846 | 0.76923 | 0.75 | 0.75 |
|  |  | EPC | GAG-GAG | GAG-AAG | GAG-AAA | GUG-GAG | GAG-GUU | GUU-GAG | CUC-GAG | GAG-AUA | GAG-CUG | AAA-AUC |
|  |  |  | 1 | 0.79188 | 0.77157 | 0.64975 | 0.64467 | 0.60914 | 0.60914 | 0.5533 | 0.54315 | 0.54315 |
|  |  | NM | GAG-GAG | GUU-GAG | GAG-AAG | GAG-AAA | GUG-GAG | CUC-GAG | GUC-GAC | GUU-GUC | GCG-GAG | AAC-AUC |
|  |  |  | 1 | 0.67797 | 0.67797 | 0.64407 | 0.54237 | 0.54237 | 0.52542 | 0.50847 | 0.49153 | 0.49153 |
|  |  | Tr | GAG-GAG | GAG-AAG | GAG-AAA | GUU-GAG | GAG-CUG | AAG-GAG | AAA-AAA | GUG-GAG | GAG-GUG | CUC-GAG |
|  |  |  | 1 | 0.81034 | 0.68966 | 0.63793 | 0.60345 | 0.60345 | 0.55172 | 0.53448 | 0.53448 | 0.53448 |
|  |  | WG | GAG-GAG | GAG-AAG | GAG-AAA | GUU-GAG | GUG-GAG | GAG-GUU | GAG-CUG | CUC-GAG | GAG-CUU | GAG-AUA |
|  |  |  | 1 | 0.80539 | 0.74753 | 0.6785 | 0.63116 | 0.62327 | 0.6075 | 0.56739 | 0.56607 | 0.53386 |
| 8 | CandidatusKorarchaeumcryptofilum OPF8 | AAM | GAG-AGG | GAG-GAG | GAG-AUA | AGG-GAG | AGG-AUA | AGG-AGG | AUA-GCU | AAG-GAG | AUA-GAG | GAU-GAG |
|  |  |  | 1 | 0.99647 | 0.82332 | 0.81625 | 0.74205 | 0.67845 | 0.64664 | 0.63604 | 0.63251 | 0.61484 |
|  |  | CM | GAG-AUA | GAG-GAG | GAG-AGG | AUA-GCU | AUA-GAG | AAG-GAG | AUA-AUA | GGG-AUA | AUA-UUC | AGG-AUA |
|  |  |  | 1 | 0.98551 | 0.94203 | 0.84058 | 0.7971 | 0.73913 | 0.72464 | 0.69565 | 0.69565 | 0.69565 |
|  |  | EPC | GAG-AGG | GAG-GAG | AGG-GAG | GAG-AUA | AAG-GAG | GAG-AAG | AGG-AGG | AUA-GAG | AUA-GCU | AGG-AUA |
|  |  |  | 1 | 0.93361 | 0.82573 | 0.6639 | 0.62656 | 0.62241 | 0.62241 | 0.61826 | 0.60166 | 0.59336 |
|  |  | NM | GAG-GAG | GAG-AGG | AGG-GAG | GAG-AUA | GAU-GAG | AAG-GAG | AUA-GAG | AGG-AUA | AUA-GAU | GAG-CUC |
|  |  |  | 1 | 1 | 0.96721 | 0.83607 | 0.7377 | 0.7377 | 0.67213 | 0.67213 | 0.62295 | 0.59016 |
|  |  | Tr | GAG-GAG | GAG-AUA | GAG-AGG | AUA-GAG | AGG-AGG | AAG-GAG | AGG-GAG | AGG-AAG | AGG-AUA | GAU-GAG |
|  |  |  | 1 | 0.7874 | 0.77165 | 0.67717 | 0.64567 | 0.56693 | 0.54331 | 0.54331 | 0.52756 | 0.51181 |
|  |  | WG | GAG-GAG | GAG-AGG | AGG-GAG | GAG-AUA | AGG-AGG | AUA-GAG | AGG-AUA | GAU-GAG | AAG-GAG | AGG-AAG |
|  |  |  | 1 | 0.93185 | 0.78209 | 0.77971 | 0.68185 | 0.66363 | 0.62797 | 0.61331 | 0.60539 | 0.56458 |
| 9 | CandidatusMethanoregulaboonei 6A8 | AAM | CUU-GCC | GAG-AUC | GGG-AUC | GCA-AAG | AUC-CGG | GCC-GGG | AAG-AUC | GUG-AUC | AUC-GGG | GCC-GGC |
|  |  |  | 1 | 0.92857 | 0.78022 | 0.60989 | 0.5989 | 0.58791 | 0.58242 | 0.57692 | 0.53846 | 0.51648 |
|  |  | CM | CUU-GCC | GAG-AUC | GGG-AUC | GCC-GGG | UUU-GCC | GCC-GGC | AUC-AUC | UUU-GUC | AUU-GCC | AUC-CUU |
|  |  |  | 1 | 0.70707 | 0.69697 | 0.69697 | 0.63636 | 0.63636 | 0.58586 | 0.53535 | 0.53535 | 0.53535 |
|  |  | EPC | CUU-GCC | GAG-AUC | AAG-AUC | GCC-GGG | GCC-GGC | AUC-AUC | GGG-AUC | GUG-AUC | GCC-CGG | AUC-CGG |
|  |  |  | 1 | 0.88372 | 0.69767 | 0.67442 | 0.66977 | 0.62791 | 0.6186 | 0.6 | 0.59535 | 0.5907 |
|  |  | NM | GAG-AUC | CUU-GCC | GGG-AUC | GCC-CGG | AAG-AUC | AUC-GAU | AUC-GAG | AUU-GCC | GAU-AUC | GCC-GGC |
|  |  |  | 1 | 0.94203 | 0.7971 | 0.78261 | 0.76812 | 0.75362 | 0.68116 | 0.63768 | 0.62319 | 0.6087 |
|  |  | Tr | GAG-AUC | AUC-CGG | AAG-AUC | CUU-GCC | GUG-AUC | AUC-GAG | GAU-AUC | AUC-GAU | CUG-AUC | GAU-CUC |
|  |  |  | 1 | 0.85714 | 0.82418 | 0.78022 | 0.75824 | 0.7033 | 0.68132 | 0.68132 | 0.65934 | 0.64835 |
|  |  | WG | GAG-AUC | CUU-GCC | AUC-CGG | GGG-AUC | AAG-AUC | GAU-AUC | GCC-GGG | GCC-CGG | GUG-AUC | AUC-AUC |
|  |  |  | 1 | 0.95486 | 0.66393 | 0.6192 | 0.60484 | 0.59253 | 0.58843 | 0.58227 | 0.57776 | 0.57653 |
| 10 | CandidatusNitrososphaeragargensis Ga9-2 | AAM | GGC-AAG | AAA-AAG | GCC-AAG | GAC-AAG | GUC-AAG | AAG-AUA | GAA-AAG | GCC-GGC | CUU-GCA | AUC-AAG |
|  |  |  | 1 | 0.73481 | 0.71271 | 0.69613 | 0.68508 | 0.67403 | 0.63536 | 0.61326 | 0.59669 | 0.55801 |
|  |  | CM | GGC-AAG | AAA-AAG | GAA-AAG | GGC-GGC | GAC-GGC | GAC-AAG | CUU-GCA | GUC-AAG | GGC-AUG | GGC-UUU |
|  |  |  | 1 | 0.76623 | 0.75325 | 0.68831 | 0.67532 | 0.63636 | 0.62338 | 0.61039 | 0.61039 | 0.5974 |
|  |  | EPC | GGC-AAG | GUC-AAG | CUU-GGC | GCC-GGC | GAC-AAG | GGC-AUC | GAA-AAG | GGC-GGC | CUU-GCA | AAA-AAG |
|  |  |  | 1 | 0.75333 | 0.68 | 0.64 | 0.62 | 0.58667 | 0.58667 | 0.58 | 0.57333 | 0.56 |
|  |  | NM | GGC-AAG | AAA-AAG | GGC-AUC | GAC-AAG | AUC-AAG | GCA-AAG | AAG-AUA | GUC-AAG | GCC-AAG | AAG-GAC |
|  |  |  | 1 | 0.7284 | 0.59259 | 0.59259 | 0.58025 | 0.55556 | 0.55556 | 0.54321 | 0.51852 | 0.51852 |
|  |  | Tr | GAA-AAG | AAA-AAG | GGC-AAG | AAG-AUA | GUC-AAG | AAG-GAC | GGC-AGG | AUC-AAG | GAC-AAG | GAA-GAA |
|  |  |  | 1 | 0.82456 | 0.81579 | 0.7193 | 0.69298 | 0.65789 | 0.64912 | 0.64912 | 0.63158 | 0.63158 |
|  |  | WG | GGC-AAG | GAA-AAG | AAA-AAG | GAC-AAG | AAG-AUA | GCC-AAG | GUC-AAG | CUU-GCA | AUC-AAG | GCA-AAG |
|  |  |  | 1 | 0.84858 | 0.77219 | 0.72644 | 0.68344 | 0.66697 | 0.63998 | 0.629 | 0.62855 | 0.61436 |
| 11 | Cenarchaeumsymbiosum A | AAM | GGC-AGG | GGC-GAG | GGC-GGC | GAC-GAG | GGG-AUA | AAG-AUA | GAG-AUA | AAA-AAG | GAG-GCG | GGC-AAG |
|  |  |  | 1 | 0.86154 | 0.83077 | 0.83077 | 0.82308 | 0.77692 | 0.76923 | 0.76923 | 0.75385 | 0.73077 |
|  |  | CM | GAC-GGC | GGC-GGC | GGC-AGG | GAC-GGG | GCG-GCA | GAG-GCG | GGC-CUG | GGC-GAG | GAC-AGG | GGC-GGG |
|  |  |  | 1 | 0.87097 | 0.82258 | 0.70968 | 0.69355 | 0.66129 | 0.64516 | 0.62903 | 0.62903 | 0.59677 |
|  |  | EPC | GGC-GGC | GGG-AUA | GGC-GAG | GGC-AAG | GAC-GGC | GAC-GAG | GAG-AUA | AUA-GGC | GAG-GGC | AUA-CUG |
|  |  |  | 1 | 0.92784 | 0.86598 | 0.85567 | 0.80412 | 0.74227 | 0.73196 | 0.72165 | 0.68041 | 0.68041 |
|  |  | NM | GGC-GGC | GGC-GAG | GCG-GGC | GGC-AGG | GGC-AAG | GAG-GGC | GAG-AUA | GGC-GGG | GGC-GCC | AUA-GGC |
|  |  |  | 1 | 0.89091 | 0.85455 | 0.83636 | 0.76364 | 0.76364 | 0.74545 | 0.72727 | 0.72727 | 0.72727 |
|  |  | Tr | GGC-AAG | GAG-AUA | AAG-GAG | GAC-AAG | GAC-GAG | AAG-AUA | AAA-AAG | GGC-AGG | GGC-GAG | GAA-AAG |
|  |  |  | 1 | 0.70968 | 0.69892 | 0.68817 | 0.66667 | 0.66667 | 0.65591 | 0.63441 | 0.5914 | 0.58065 |
|  |  | WG | GAC-GGC | GGC-GGC | GGC-GAG | GGC-AGG | GAC-GAG | GAC-GGG | GAG-AUA | GAG-GAG | GAC-AGG | GAG-GGC |
|  |  |  | 1 | 0.84971 | 0.75011 | 0.7234 | 0.68311 | 0.68176 | 0.6718 | 0.56225 | 0.55591 | 0.54233 |
| 12 | Desulfurococcusfermentans Z-1312 | AAM | GAG-AAA | GAG-AUA | GAG-GAG | AUA-AUA | GAG-AAG | AGG-GAG | AAG-GAG | AUA-GCU | GAU-GAA | AUA-GAU |
|  |  |  | 1 | 0.90588 | 0.85882 | 0.81176 | 0.77647 | 0.72941 | 0.71765 | 0.70588 | 0.69412 | 0.65882 |
|  |  | CM | GAG-GAG | AUA-GCU | GAG-AUA | GAG-AAA | GAU-AUA | AUA-AUA | GAG-AAG | AUA-GAG | AGG-AUA | AUA-UAU |
|  |  |  | 1 | 0.86364 | 0.80303 | 0.78788 | 0.75758 | 0.74242 | 0.71212 | 0.71212 | 0.71212 | 0.69697 |
|  |  | EPC | GAG-AAA | GAG-GAG | AAG-AUA | GAG-AUA | AUA-AUA | AUA-GAG | GUU-GAA | AUA-GCU | AUA-GAU | AGG-GAG |
|  |  |  | 1 | 0.92593 | 0.90741 | 0.89815 | 0.80556 | 0.78704 | 0.75 | 0.73148 | 0.69444 | 0.69444 |
|  |  | NM | AGG-GAG | GAU-AUA | GAG-GAG | GAG-AUA | AUA-GAG | AAG-AUA | GAG-AAG | AUA-GAU | AGG-GAU | GUU-GAG |
|  |  |  | 1 | 0.97872 | 0.97872 | 0.91489 | 0.87234 | 0.87234 | 0.85106 | 0.80851 | 0.78723 | 0.76596 |
|  |  | Tr | GAG-AUA | GAG-AAA | GAG-GAG | GAG-GAA | AAG-GAG | AUA-GAG | AGG-GAG | AUA-AUA | AUA-GAU | GAG-AAG |
|  |  |  | 1 | 0.88889 | 0.80556 | 0.77778 | 0.70833 | 0.68056 | 0.68056 | 0.625 | 0.56944 | 0.55556 |
|  |  | WG | GAG-GAG | GAG-AAA | GAG-AUA | AUA-GAG | GAG-AAG | AUA-AUA | AAG-AUA | AGG-GAG | GAU-AUA | AUA-GAU |
|  |  |  | 1 | 0.96806 | 0.90594 | 0.84472 | 0.80745 | 0.80302 | 0.74445 | 0.74091 | 0.72316 | 0.72138 |
| 13 | Desulfurococcusmucosus 07/1, DSM 2162 | AAM | AGG-GAG | GAG-GAG | GAG-AAG | AUA-GUG | GAG-AUA | GGC-UUC | CUC-UAC | AAG-GAG | GUU-GAA | GAG-AAA |
|  |  |  | 1 | 0.98864 | 0.94318 | 0.72727 | 0.70455 | 0.69318 | 0.68182 | 0.68182 | 0.67045 | 0.67045 |
|  |  | CM | AGG-GAG | GAG-GAG | GGC-UUC | CUC-UAC | UUC-AAG | GAG-AAG | AGG-AGG | GUG-GAG | GAG-UAC | GUC-UAC |
|  |  |  | 1 | 0.8254 | 0.77778 | 0.71429 | 0.66667 | 0.66667 | 0.65079 | 0.63492 | 0.63492 | 0.61905 |
|  |  | EPC | GAG-GAG | AGG-GAG | GUG-GAG | GAG-AAG | GGC-UUC | GUU-GAA | GUG-AAG | AGG-AUA | CUC-UAC | UAC-AGG |
|  |  |  | 1 | 0.87786 | 0.70229 | 0.65649 | 0.64885 | 0.58015 | 0.58015 | 0.55725 | 0.54198 | 0.53435 |
|  |  | NM | AGG-GAG | GAG-GAG | AGG-AUA | GUG-GAG | AGG-GUU | AGG-AGG | GAG-CUC | AUA-GUG | GUC-GAC | GAG-AAG |
|  |  |  | 1 | 0.85714 | 0.79592 | 0.77551 | 0.7551 | 0.71429 | 0.65306 | 0.65306 | 0.61224 | 0.59184 |
|  |  | Tr | GAG-GAG | GAG-AAG | GAG-AUA | AAG-GAG | AGG-GAG | AUA-GAG | AGG-AAG | AAG-AUA | GAG-CUC | AUA-AGG |
|  |  |  | 1 | 0.7234 | 0.70213 | 0.59574 | 0.55319 | 0.5 | 0.46809 | 0.41489 | 0.40426 | 0.39362 |
|  |  | WG | GAG-GAG | AGG-GAG | GAG-AAG | AGG-AGG | GUG-GAG | UAC-AGG | CUC-UAC | AAG-GAG | GGC-UUC | GAG-AUA |
|  |  |  | 1 | 0.83859 | 0.73376 | 0.60643 | 0.59678 | 0.57299 | 0.56656 | 0.55305 | 0.55177 | 0.54727 |
| 14 | Ferroglobusplacidus AEDII12DO, DSM 10642 | AAM | GAA-GAG | GGA-GUU | GAG-GAG | GAA-AAG | GAA-AAA | AAA-GCU | CUC-GGA | GAA-GUU | GCU-UUG | GAA-GCU |
|  |  |  | 1 | 0.99286 | 0.97857 | 0.92857 | 0.91429 | 0.90714 | 0.89286 | 0.87857 | 0.81429 | 0.81429 |
|  |  | CM | CUC-GGA | GAA-GAA | GAA-AAA | AAG-AAG | GAG-GAG | GGA-GUU | GGA-AUA | GGA-GAG | GAA-GUU | GAA-GAG |
|  |  |  | 1 | 0.89796 | 0.81633 | 0.81633 | 0.77551 | 0.7551 | 0.7551 | 0.73469 | 0.73469 | 0.71429 |
|  |  | EPC | GAA-GAG | GAG-GAG | GAA-AAA | GAG-AAG | GAA-GAA | GAA-GUU | AAG-GAG | AAA-GCU | GAG-CUU | GAA-GCU |
|  |  |  | 1 | 0.93991 | 0.82833 | 0.81974 | 0.78112 | 0.75966 | 0.7382 | 0.72103 | 0.71674 | 0.70815 |
|  |  | NM | GAG-GAG | GAA-GAG | GAG-GAA | AAG-GAG | GAG-AAG | GAA-AAA | GAG-AUA | GUU-GAG | GAA-GUU | CUC-GAA |
|  |  |  | 1 | 0.96774 | 0.95161 | 0.8871 | 0.83871 | 0.82258 | 0.80645 | 0.75806 | 0.74194 | 0.67742 |
|  |  | Tr | GAA-AAG | GAG-GAG | GAG-AAG | GAA-GAG | CUC-GAA | AAG-GAG | GAA-AAA | AAA-GAG | AAG-AAG | GAG-GAA |
|  |  |  | 1 | 0.97403 | 0.94805 | 0.92208 | 0.8961 | 0.87013 | 0.84416 | 0.79221 | 0.74026 | 0.7013 |
|  |  | WG | GAA-GAG | GAG-GAG | GAA-AAA | GAA-AAG | GAG-AAG | GAA-GUU | GAA-GAA | AAA-GAG | AAG-GAG | GAG-GAA |
|  |  |  | 1 | 0.98965 | 0.94685 | 0.85136 | 0.83208 | 0.79257 | 0.77799 | 0.76811 | 0.746 | 0.70132 |
| 15 | Fervidicoccusfontis Kam940 | AAM | GAA-AAA | AAA-GAA | GAA-GAA | AAA-AAA | GAA-AUA | AUA-AAA | GGA-GGA | GGA-AUA | GAA-AAU | AUA-AUA |
|  |  |  | 1 | 0.80392 | 0.78431 | 0.76471 | 0.7451 | 0.66667 | 0.64706 | 0.63725 | 0.61765 | 0.61765 |
|  |  | CM | GAA-AAA | AAA-AUA | AAA-GAA | AUA-GAU | GAA-GAA | AUA-AAA | AUA-AUA | AUA-GGA | AAA-AAA | UUA-AAA |
|  |  |  | 1 | 0.68333 | 0.66667 | 0.65 | 0.63333 | 0.63333 | 0.56667 | 0.55 | 0.53333 | 0.51667 |
|  |  | EPC | GAA-AAA | GAA-GAA | AAA-GAA | AUA-AAA | AUA-GAA | GAA-AUA | GAA-AAG | GGA-AUA | AAA-AAA | AAA-AUU |
|  |  |  | 1 | 0.84559 | 0.77206 | 0.75 | 0.72794 | 0.71324 | 0.69853 | 0.69118 | 0.69118 | 0.66912 |
|  |  | NM | GAA-AAA | AAA-AUA | AAA-GAA | AUA-AAA | GAA-GAA | AUA-GAA | GGA-AAA | AAA-AAA | GAG-CUU | AAA-AUU |
|  |  |  | 1 | 0.9 | 0.88333 | 0.8 | 0.78333 | 0.71667 | 0.7 | 0.65 | 0.63333 | 0.61667 |
|  |  | Tr | GAA-GAA | GAA-AAA | AAA-GAA | AAA-AAA | GAA-AAG | AAA-AUA | GAA-AUA | GAU-GAA | AUA-AAA | GAA-GAU |
|  |  |  | 1 | 1 | 0.94805 | 0.76623 | 0.75325 | 0.71429 | 0.66234 | 0.63636 | 0.63636 | 0.62338 |
|  |  | WG | GAA-AAA | AAA-GAA | GAA-GAA | AAA-AAA | AUA-AAA | GAA-AAG | GAA-AUA | AAA-AUA | AUA-GAA | AAA-AUU |
|  |  |  | 1 | 0.82946 | 0.81202 | 0.73256 | 0.63566 | 0.62209 | 0.62016 | 0.60142 | 0.58915 | 0.56395 |
| 16 | Halalkalicoccusjeotgali B3, DSM 18796 | AAM | GGC-GAG | GUC-GAG | GAG-GAG | GAC-GAG | GCC-GAG | GCC-GAC | GAG-GAC | CGC-GAG | GAC-GUC | GUC-GAC |
|  |  |  | 1 | 0.95298 | 0.94937 | 0.86257 | 0.81193 | 0.74503 | 0.7396 | 0.73056 | 0.72875 | 0.72514 |
|  |  | CM | GUC-GAG | GUC-GUC | CUC-GCG | GGC-GAG | GCG-AUC | GCC-GAG | GCC-GAC | GAG-GAG | GAG-GAC | GCG-GUC |
|  |  |  | 1 | 0.94545 | 0.87636 | 0.83273 | 0.81818 | 0.81091 | 0.78909 | 0.78909 | 0.75636 | 0.74909 |
|  |  | EPC | GAG-GAG | GUC-GAG | GGC-GAG | GAC-GAG | GAG-GAC | GAC-GAC | GCC-GAC | GCC-GAG | AUC-GAG | CGC-GAG |
|  |  |  | 1 | 0.94145 | 0.88525 | 0.83372 | 0.79391 | 0.7541 | 0.70726 | 0.69555 | 0.68852 | 0.68618 |
|  |  | NM | GUC-GAG | GAG-GAG | GGC-GAG | GAC-GAG | GCC-GAC | CGC-GAG | GAG-GAC | GCC-GAG | CUC-GAC | GAC-GCC |
|  |  |  | 1 | 0.92258 | 0.89677 | 0.87742 | 0.7871 | 0.77419 | 0.72258 | 0.70968 | 0.70323 | 0.69677 |
|  |  | Tr | GAG-GAG | GAC-GAG | GAG-GAC | GUC-GAG | GAG-AUC | GAA-CUC | GAC-GAC | CUC-GAA | CUC-GAC | CGC-GAG |
|  |  |  | 1 | 0.85778 | 0.8 | 0.74667 | 0.71111 | 0.70222 | 0.68444 | 0.64889 | 0.62222 | 0.61333 |
|  |  | WG | GUC-GAG | GAG-GAG | GGC-GAG | GAC-GAG | GAG-GAC | GUC-GUC | GAC-GAC | GCC-GAC | CUC-GCG | CUC-GAC |
|  |  |  | 1 | 0.9706 | 0.88839 | 0.87547 | 0.82846 | 0.79251 | 0.77097 | 0.72865 | 0.72715 | 0.72172 |
| 17 | Haloarculamarismortui ATCC 43049 | AAM | GAC-GAC | GUC-GAC | GCC-GAC | GAC-GAG | GAC-GCC | GUC-GUC | GGC-GUC | GAC-GGC | GUC-GAG | GGC-GAC |
|  |  |  | 1 | 0.92576 | 0.88428 | 0.85371 | 0.80568 | 0.79476 | 0.78384 | 0.78384 | 0.74891 | 0.74672 |
|  |  | CM | GUC-GAC | GAC-GAC | GAC-GGC | GUC-GUC | GCC-GAC | GGC-GAC | GAC-GAG | GAC-GCC | GGC-GUC | GUC-GAG |
|  |  |  | 1 | 0.97992 | 0.89558 | 0.87149 | 0.85944 | 0.79518 | 0.7751 | 0.75502 | 0.70683 | 0.6506 |
|  |  | EPC | GAC-GAC | GAC-GAG | GUC-GAC | GUC-GUC | GAC-GGC | GCC-GAC | GAG-GAC | GGC-GUC | GAC-CUC | GGC-GGC |
|  |  |  | 1 | 0.82353 | 0.77828 | 0.67873 | 0.67647 | 0.66742 | 0.63575 | 0.61991 | 0.61991 | 0.61765 |
|  |  | NM | GAC-GAC | GUC-GAC | GCC-GAC | GAC-GCC | GAC-GAG | GGC-GAC | GAC-CUC | GUC-GUC | GAC-GGC | GGC-GAG |
|  |  |  | 1 | 0.72193 | 0.68449 | 0.66845 | 0.63636 | 0.62567 | 0.58824 | 0.56684 | 0.5508 | 0.54545 |
|  |  | Tr | GAC-GAG | GAC-GAC | GAG-GAC | GUC-GAC | GUC-GAG | CUC-GAC | AUC-GAC | GAA-CUC | GAC-CUC | GAG-GAG |
|  |  |  | 1 | 0.89423 | 0.73558 | 0.69231 | 0.67308 | 0.64904 | 0.64904 | 0.63942 | 0.63462 | 0.60096 |
|  |  | WG | GAC-GAC | GAC-GAG | GUC-GAC | GCC-GAC | GUC-GUC | GAC-GGC | GUC-GAG | GAC-GCC | GGC-GAC | GAC-CUC |
|  |  |  | 1 | 0.79656 | 0.78243 | 0.76172 | 0.69637 | 0.66779 | 0.62058 | 0.60678 | 0.59634 | 0.5896 |
| 18 | Halobacteriumsalinarum R1, DSM 671 | AAM | GAC-GCC | GCC-GAC | GAC-GAC | GCC-GCC | GUC-GAC | GAC-GGC | GCC-GGC | GCG-GCG | GGC-GAC | GAC-GCG |
|  |  |  | 1 | 0.88203 | 0.75318 | 0.7314 | 0.61525 | 0.60254 | 0.55354 | 0.53358 | 0.53176 | 0.52995 |
|  |  | CM | GAC-GCC | GCC-GAC | GCC-GCC | GCC-GGC | GGC-GAC | GAC-GAC | GAC-GGC | GUC-GAC | GCC-GUC | GUC-GCC |
|  |  |  | 1 | 0.84615 | 0.75 | 0.63942 | 0.61058 | 0.60096 | 0.59615 | 0.56731 | 0.53365 | 0.52885 |
|  |  | EPC | GAC-GAC | GAC-GCC | GCC-GAC | GCC-GCC | GGC-GGC | GUC-GAC | GCC-GGC | GGC-GAC | GAC-GAG | GAC-GGC |
|  |  |  | 1 | 0.90691 | 0.86436 | 0.69681 | 0.64628 | 0.63298 | 0.61968 | 0.60904 | 0.60638 | 0.60372 |
|  |  | NM | GAC-GCC | GCC-GAC | GAC-GAC | GAC-GGC | GUC-GAC | GCC-GCC | GGC-GAC | GCC-GGC | GAC-GCG | GGC-GUC |
|  |  |  | 1 | 0.85169 | 0.77966 | 0.64831 | 0.57627 | 0.55508 | 0.50847 | 0.44492 | 0.44492 | 0.4322 |
|  |  | Tr | GAC-GAC | GAC-GAG | GAC-GCC | GCC-GAC | GAG-GAC | CUC-GAC | GAC-CUC | GUC-GAC | GAC-GGC | GAG-GAG |
|  |  |  | 1 | 0.6875 | 0.675 | 0.64583 | 0.60417 | 0.60417 | 0.59167 | 0.54583 | 0.50417 | 0.48333 |
|  |  | WG | GAC-GCC | GAC-GAC | GCC-GAC | GCC-GCC | GAC-GGC | GUC-GAC | GGC-GAC | GAC-GAG | GAC-GCG | CUC-GAC |
|  |  |  | 1 | 0.97446 | 0.92045 | 0.68421 | 0.66333 | 0.62537 | 0.58257 | 0.57532 | 0.53978 | 0.52407 |
| 19 | Haloferaxvolcanii DS2, ATCC 29605 | AAM | CUC-GAC | GUC-GUC | GAC-GAC | GCC-GAC | GGC-GUC | GAC-GCC | GCG-CUC | GUC-GAC | GUC-GGC | GGC-GGC |
|  |  |  | 1 | 0.96977 | 0.94767 | 0.93256 | 0.92326 | 0.89302 | 0.8314 | 0.81628 | 0.8093 | 0.80116 |
|  |  | CM | GAC-GAC | CUC-GAC | GUC-GUC | GCC-GAC | CUC-GUC | GCG-CUC | GGC-GCG | GUC-GGC | GGC-GUC | GGC-GAC |
|  |  |  | 1 | 0.97753 | 0.97303 | 0.91685 | 0.91236 | 0.89888 | 0.85169 | 0.8427 | 0.84045 | 0.82921 |
|  |  | EPC | GAC-GAC | GUC-GUC | CUC-GAC | GAC-GAG | GGC-GUC | GCC-GAC | GGC-GGC | GGC-GAC | GUC-GAC | CUC-GUC |
|  |  |  | 1 | 0.87391 | 0.8669 | 0.79685 | 0.79335 | 0.77933 | 0.72855 | 0.72329 | 0.69877 | 0.69527 |
|  |  | NM | GCC-GAC | GAC-GAC | CUC-GAC | GAC-GCC | GUC-GUC | GUC-GAC | GAC-GAG | GGC-GUC | GAC-CUC | GAC-GGC |
|  |  |  | 1 | 0.97021 | 0.8383 | 0.82553 | 0.79149 | 0.74894 | 0.7234 | 0.70638 | 0.69787 | 0.65957 |
|  |  | Tr | GAC-GAC | CUC-GAC | GAC-GAG | GUC-GAC | GAA-CUC | CUC-GAA | GAC-CUC | GGC-GUC | GUC-GUC | GCG-CUC |
|  |  |  | 1 | 0.84985 | 0.84685 | 0.65766 | 0.61562 | 0.60961 | 0.59459 | 0.56456 | 0.54655 | 0.51351 |
|  |  | WG | GAC-GAC | GUC-GUC | CUC-GAC | GCC-GAC | CUC-GUC | GAC-GAG | GAC-GCC | GGC-GUC | GCG-CUC | GGC-GAC |
|  |  |  | 1 | 0.86293 | 0.85958 | 0.79226 | 0.73799 | 0.73788 | 0.72136 | 0.69596 | 0.69342 | 0.67506 |
| 20 | Halogeometricumborinquense PR3, DSM 11551 | AAM | GAC-GAC | GUC-GUC | GAA-CUC | GGC-GUC | CUC-GUC | GUC-GAA | CUC-GAC | GCC-GUC | GAC-GAG | GAC-GCC |
|  |  |  | 1 | 0.8738 | 0.78394 | 0.76482 | 0.73805 | 0.71128 | 0.70554 | 0.6979 | 0.6979 | 0.68451 |
|  |  | CM | GAC-GAC | GUC-GUC | GCC-GAC | GCC-GUC | CUC-GAC | GGC-GAC | GUC-GAA | CUC-GUC | GAC-GGC | GAC-GCC |
|  |  |  | 1 | 0.82955 | 0.75 | 0.71591 | 0.70833 | 0.69318 | 0.65152 | 0.64015 | 0.63258 | 0.625 |
|  |  | EPC | GAC-GAC | GUC-GUC | GAC-GAG | CUC-GAA | CUC-GAC | GGC-GUC | GCC-GUC | CUC-GUC | GGC-GAC | GCC-GAC |
|  |  |  | 1 | 0.94164 | 0.73475 | 0.69496 | 0.67109 | 0.66578 | 0.66578 | 0.66578 | 0.64721 | 0.63395 |
|  |  | NM | GAC-GAC | GAC-GCC | GAC-GAG | GUC-GUC | GAA-CUC | CUC-GAC | GCC-GAC | GAG-ACG | GAC-GGC | GUC-GAA |
|  |  |  | 1 | 0.75625 | 0.75625 | 0.74375 | 0.73125 | 0.7 | 0.675 | 0.65 | 0.6375 | 0.63125 |
|  |  | Tr | GAC-GAC | GAA-CUC | GAC-GAG | CUC-GAA | CUC-GAC | GAC-GAA | GAG-ACG | GUC-GAA | GAG-GAC | GUC-GUC |
|  |  |  | 1 | 0.8583 | 0.80162 | 0.78138 | 0.7247 | 0.67206 | 0.64777 | 0.63158 | 0.57085 | 0.5587 |
|  |  | WG | GAC-GAC | GUC-GUC | GAC-GAG | CUC-GUC | GAA-CUC | GUC-GAA | CUC-GAC | GAG-ACG | GAC-GAA | GCC-GAC |
|  |  |  | 1 | 0.79616 | 0.69416 | 0.66202 | 0.65792 | 0.64251 | 0.63447 | 0.60216 | 0.59364 | 0.59331 |
| 21 | Halomicrobiummukohataei arg-2, DSM 12286 | AAM | GCC-GAC | GAC-GAC | GUC-GAC | GAC-GAG | GAC-GCC | GAC-GUG | GUC-GUC | GUC-GAG | GGC-GUC | GCC-GUC |
|  |  |  | 1 | 0.974 | 0.92721 | 0.90988 | 0.83536 | 0.81282 | 0.80069 | 0.7747 | 0.7591 | 0.73137 |
|  |  | CM | GAC-GGC | GCC-GAC | GUC-GAC | GUC-GUC | GAC-GAC | GCC-GUC | GGC-GAC | GUC-GAG | GAC-GCC | GAC-GAG |
|  |  |  | 1 | 0.95427 | 0.87195 | 0.85976 | 0.85366 | 0.79268 | 0.77744 | 0.76829 | 0.7561 | 0.75 |
|  |  | EPC | GAC-GAC | GAC-GAG | GUC-GUC | GUC-GAC | GCC-GAC | GUC-GAG | GGC-GUC | GAC-GCC | CUC-GAC | GCC-GUC |
|  |  |  | 1 | 0.95561 | 0.91384 | 0.89295 | 0.86162 | 0.78068 | 0.74935 | 0.73368 | 0.71802 | 0.6893 |
|  |  | NM | GAC-GAC | GAC-GAG | GUC-GAC | GCC-GAC | GUC-GAG | GAC-GCC | GUC-GUC | GCC-GAG | CUC-GAC | GGC-GAG |
|  |  |  | 1 | 0.95028 | 0.83978 | 0.82873 | 0.74033 | 0.74033 | 0.71271 | 0.69613 | 0.69061 | 0.65746 |
|  |  | Tr | GAC-GAC | GAC-GAG | GCC-GAC | GUC-GAC | CUC-GAC | GAC-GUG | GUC-GAG | GUC-GUC | GAC-GCC | GAG-GAG |
|  |  |  | 1 | 0.947 | 0.74205 | 0.73145 | 0.73145 | 0.71378 | 0.70318 | 0.65018 | 0.60777 | 0.55477 |
|  |  | WG | GAC-GAC | GAC-GAG | GUC-GUC | GCC-GAC | GUC-GAC | GUC-GAG | GAC-GCC | GAC-GGC | GCC-GUC | CUC-GAC |
|  |  |  | 1 | 0.87362 | 0.84877 | 0.84482 | 0.82773 | 0.74442 | 0.69839 | 0.67594 | 0.66591 | 0.65617 |
| 22 | Haloquadratumwalsbyi C23, DSM 16854 | AAM | GUU-GAU | CUU-GAU | GAU-GAU | GCU-GAU | GAU-GAG | GAU-GCA | GAU-GAA | AUU-GAU | GAU-AUU | GAG-ACA |
|  |  |  | 1 | 0.98016 | 0.9246 | 0.87698 | 0.86905 | 0.84921 | 0.82143 | 0.79762 | 0.75 | 0.71429 |
|  |  | CM | CUU-GAU | GUU-GAU | GAU-GAU | GCU-GAU | GAU-GCA | GAU-AUU | GGU-GAU | CUU-GCA | AUU-GAU | GAU-GAG |
|  |  |  | 1 | 0.97059 | 0.93137 | 0.88235 | 0.83333 | 0.81373 | 0.7451 | 0.73529 | 0.71569 | 0.68627 |
|  |  | EPC | GAU-GAU | GAU-GAA | GAU-GCA | GUU-GAU | CUU-GAU | GAU-GAG | GCU-GAU | GAU-AUU | GGU-GAU | GCA-GCA |
|  |  |  | 1 | 0.8366 | 0.81046 | 0.69935 | 0.69935 | 0.61438 | 0.60131 | 0.59477 | 0.56209 | 0.56209 |
|  |  | NM | GAU-GAU | GUU-GAU | CUU-GAU | GAU-GAG | GCU-GAU | GAU-GCA | GAU-GAA | GAU-AUC | AUU-GAU | GAU-AUU |
|  |  |  | 1 | 0.98 | 0.87 | 0.79 | 0.78 | 0.76 | 0.72 | 0.64 | 0.63 | 0.58 |
|  |  | Tr | GAU-GAA | GAU-GAG | GAU-GAU | CUU-GAU | AUU-GAU | GUU-GAU | AUU-GAG | GAU-AUU | GAG-GAU | CUU-GAG |
|  |  |  | 1 | 0.81061 | 0.77273 | 0.76515 | 0.69697 | 0.68939 | 0.60606 | 0.59848 | 0.57576 | 0.57576 |
|  |  | WG | GAU-GAU | GAU-GAA | CUU-GAU | GUU-GAU | GAU-GAG | AUU-GAU | GAU-GCA | GCU-GAU | GAU-AUU | GUU-GAG |
|  |  |  | 1 | 0.81477 | 0.80809 | 0.79287 | 0.77877 | 0.70824 | 0.70267 | 0.69191 | 0.66667 | 0.59985 |
| 23 | Haloquadratumwalsbyi HBSQ001, DSM 16790 | AAM | GUU-GAU | GAU-GAU | CUU-GAU | GAU-GAG | GAU-GAA | GAU-GCA | GCU-GAU | AUU-GAU | GAU-AUU | CUU-GCA |
|  |  |  | 1 | 0.984 | 0.976 | 0.848 | 0.848 | 0.84 | 0.832 | 0.784 | 0.744 | 0.7 |
|  |  | CM | GUU-GAU | GAU-GAU | CUU-GAU | GCU-GAU | GAU-AUU | GAU-GCA | GGU-GAU | CUU-GCA | GAU-GAG | AUU-GAU |
|  |  |  | 1 | 1 | 0.96875 | 0.95833 | 0.85417 | 0.83333 | 0.79167 | 0.78125 | 0.73958 | 0.70833 |
|  |  | EPC | GAU-GAU | GAU-GAA | GAU-GCA | GUU-GAU | CUU-GAU | GCU-GAU | GAU-GAG | GAU-AUU | GCA-GCA | GAU-GUU |
|  |  |  | 1 | 0.91608 | 0.81818 | 0.74126 | 0.73427 | 0.64336 | 0.60839 | 0.60839 | 0.58741 | 0.58042 |
|  |  | NM | GUU-GAU | GAU-GAU | CUU-GAU | GAU-GAG | GAU-GCA | GAU-GAA | GCU-GAU | GAU-AUU | AUU-GAU | GAU-AUC |
|  |  |  | 1 | 1 | 0.91111 | 0.82222 | 0.81111 | 0.81111 | 0.77778 | 0.68889 | 0.68889 | 0.64444 |
|  |  | Tr | GAU-GAA | GAU-GAU | GAU-GAG | CUU-GAU | GUU-GAU | AUU-GAU | GAG-GAG | GAG-GAU | CUU-GAA | GCU-GAU |
|  |  |  | 1 | 0.77049 | 0.72131 | 0.68033 | 0.64754 | 0.63934 | 0.59836 | 0.52459 | 0.51639 | 0.5082 |
|  |  | WG | Pair of Codons | GAU-GAU | CUU-GAU | GUU-GAU | GAU-GAA | GAU-GAG | AUU-GAU | GAU-GCA | GCU-GAU | GAU-AUU |
|  |  |  | 1 | 0.81776 | 0.80218 | 0.79245 | 0.7648 | 0.706 | 0.70132 | 0.6912 | 0.66939 | 0.59969 |
| 24 | Halorhabdusutahensis AX-2, DSM 12940 | AAM | GUC-GAG | GAC-GCC | GCC-GAG | GCC-GAC | GUC-GAC | GAC-GAC | GAC-GAG | GAC-GUC | GGC-GAG | GUC-GUC |
|  |  |  | 1 | 0.81818 | 0.81572 | 0.81081 | 0.79853 | 0.79361 | 0.77396 | 0.7715 | 0.75184 | 0.71253 |
|  |  | CM | GAC-GAC | GUC-GAG | GCC-GAC | GAC-GGC | GAC-GUC | GGC-GAG | GUC-GAC | GGC-GAC | GAC-GAG | GCC-GAG |
|  |  |  | 1 | 0.94218 | 0.94218 | 0.93878 | 0.87755 | 0.87415 | 0.85374 | 0.84354 | 0.82993 | 0.7381 |
|  |  | EPC | GUC-GAG | GAC-GAC | GAC-GAG | GCC-GAC | GCC-GAG | GUC-GUC | GUC-GCC | GAC-GUC | GGC-GUC | GAC-GCC |
|  |  |  | 1 | 0.95238 | 0.91156 | 0.88435 | 0.86735 | 0.85714 | 0.80272 | 0.79932 | 0.79252 | 0.78912 |
|  |  | NM | GAC-GAC | GUC-GAG | GCC-GAC | GAC-GCC | GUC-GUC | GUC-GAC | GCC-GAG | CUC-GAC | GAC-GAG | GAC-GUC |
|  |  |  | 1 | 0.98485 | 0.94697 | 0.90909 | 0.87879 | 0.86364 | 0.84848 | 0.80303 | 0.76515 | 0.75758 |
|  |  | Tr | GAC-GAG | GAC-GAC | GUC-GAG | GUC-GAC | GAC-GUC | GAG-GAC | GAA-CUC | AUC-GAG | GCC-GAG | GAG-GAG |
|  |  |  | 1 | 0.94611 | 0.94012 | 0.90419 | 0.8982 | 0.86826 | 0.86826 | 0.86228 | 0.83832 | 0.79042 |
|  |  | WG | GUC-GAG | GAC-GAC | GUC-GUC | GAC-GAG | GCC-GAC | GCC-GAG | GUC-GAC | GAC-GUC | GAC-GCC | GGC-GAG |
|  |  |  | 1 | 0.9751 | 0.87246 | 0.86964 | 0.84799 | 0.80641 | 0.8023 | 0.77674 | 0.7644 | 0.71156 |
| 25 | Halorubrumlacusprofundi ATCC 49239 | AAM | GCC-GAC | GUC-GAC | GAC-GAG | GAC-GAC | CUC-GAC | GAG-GCG | GAC-GCG | GUC-GAG | GAC-GCC | GGC-GAG |
|  |  |  | 1 | 0.92079 | 0.88944 | 0.88614 | 0.81188 | 0.81023 | 0.78878 | 0.76733 | 0.75578 | 0.74257 |
|  |  | CM | GCC-GAC | GAC-GAC | GAC-GCG | GGC-GAC | GUC-GAC | GAC-GAG | CUC-GAC | GCG-CUC | GUC-GUC | GAC-GCC |
|  |  |  | 1 | 0.96508 | 0.89206 | 0.87302 | 0.86984 | 0.79048 | 0.7873 | 0.78413 | 0.74921 | 0.73016 |
|  |  | EPC | GAC-GAG | GAC-GAC | GUC-GAC | GCC-GAC | GUC-GAG | CUC-GAC | GGC-GAG | GUC-GUC | GAG-GAG | GGC-GAC |
|  |  |  | 1 | 0.98812 | 0.93112 | 0.80048 | 0.7981 | 0.7696 | 0.74347 | 0.74109 | 0.74109 | 0.71259 |
|  |  | NM | GCC-GAC | GUC-GAC | GAC-GAG | GAC-GAC | CUC-GAC | GAC-GCC | GAC-GCG | GUC-GUC | GGC-GAC | GAG-GAG |
|  |  |  | 1 | 0.93269 | 0.86538 | 0.80288 | 0.75481 | 0.74038 | 0.68269 | 0.65385 | 0.625 | 0.625 |
|  |  | Tr | GAC-GAG | GAC-GAC | GUC-GAC | GAG-GAG | CUC-GAC | GCC-GAC | GUC-GAG | GAG-GAC | GAC-GCC | GGC-GAG |
|  |  |  | 1 | 0.76491 | 0.74386 | 0.74035 | 0.68421 | 0.61404 | 0.59298 | 0.5614 | 0.5193 | 0.50526 |
|  |  | WG | GAC-GAC | GAC-GAG | GUC-GAC | GCC-GAC | CUC-GAC | GUC-GUC | GGC-GAC | GUC-GAG | GAC-GCG | GAC-GCC |
|  |  |  | 1 | 0.90287 | 0.82851 | 0.80747 | 0.74924 | 0.67675 | 0.66494 | 0.66119 | 0.65773 | 0.6462 |
| 26 | Halovivaxruber XH-70, DSM 18193 | AAM | GUC-GAC | GCC-GAC | GUC-GUC | GAC-GAC | GAC-GUC | GAC-GAG | CUC-GUC | GCC-GUC | CUC-GAC | GAC-GCC |
|  |  |  | 1 | 0.97188 | 0.90685 | 0.89982 | 0.86819 | 0.85589 | 0.77329 | 0.77153 | 0.74517 | 0.74341 |
|  |  | CM | GUC-GAC | GCC-GAC | GUC-GUC | GAC-GAC | GCC-GUC | CUC-GUC | GAC-GUC | GAC-GAG | GAC-GCC | GAC-GGC |
|  |  |  | 1 | 0.9802 | 0.9604 | 0.94554 | 0.94059 | 0.91089 | 0.88119 | 0.85644 | 0.75248 | 0.73762 |
|  |  | EPC | GAC-GAG | GAC-GAC | GUC-GAC | GUC-GUC | GAC-GUC | GCC-GAC | CUC-GAC | GGC-GUC | GCC-GUC | GUC-GAG |
|  |  |  | 1 | 0.98457 | 0.9321 | 0.90432 | 0.8858 | 0.85494 | 0.73765 | 0.7284 | 0.71914 | 0.70988 |
|  |  | NM | GUC-GAC | GAC-GAC | GAC-GAG | GUC-GUC | GUC-GAG | GCC-GAC | GAC-GCC | GAC-GUC | CUC-GAC | GCC-GUC |
|  |  |  | 1 | 0.88172 | 0.82796 | 0.80108 | 0.75269 | 0.73118 | 0.72581 | 0.71505 | 0.71505 | 0.6129 |
|  |  | Tr | GAC-GAC | GAC-GAG | GAC-GUC | GUC-GAC | GCC-GAC | AUC-GAC | CUC-GAC | GUC-GAG | GAA-CUC | GAG-GAG |
|  |  |  | 1 | 0.95968 | 0.71371 | 0.62097 | 0.61694 | 0.60081 | 0.56048 | 0.53629 | 0.52823 | 0.50403 |
|  |  | WG | GAC-GAC | GAC-GAG | GUC-GAC | GUC-GUC | GCC-GAC | GAC-GUC | CUC-GUC | GUC-GAG | CUC-GAC | GCC-GUC |
|  |  |  | 1 | 0.91139 | 0.90592 | 0.85614 | 0.83544 | 0.80568 | 0.73144 | 0.68885 | 0.68816 | 0.68269 |
| 27 | Hyperthermusbutylicus DSM 5456 | AAM | GAG-GAG | GAG-AAG | GAG-GCU | GAG-AUA | GAG-GUU | AUA-GAG | GGC-GAG | GAG-AGG | GAG-CUG | GAC-GAG |
|  |  |  | 1 | 0.75641 | 0.5 | 0.5 | 0.48077 | 0.48077 | 0.46795 | 0.46795 | 0.4359 | 0.4359 |
|  |  | CM | GAG-GAG | GAG-AAG | GAG-GCU | CUA-GCA | GGC-AUA | GAG-CUG | GCA-GCA | CUC-AUA | CUC-UAC | GUA-GAG |
|  |  |  | 1 | 0.74138 | 0.72414 | 0.56897 | 0.55172 | 0.53448 | 0.51724 | 0.51724 | 0.5 | 0.48276 |
|  |  | EPC | GAG-GAG | GAG-AAG | GAG-GCU | GGC-GAG | CUA-GCA | GAG-CUG | GUG-GAG | CUC-GGC | UAC-GAG | CUC-UAC |
|  |  |  | 1 | 0.9645 | 0.69822 | 0.66272 | 0.56805 | 0.52663 | 0.49704 | 0.47337 | 0.46154 | 0.46154 |
|  |  | NM | GAG-GAG | GAG-AAG | GAG-CUA | GAG-AGG | GAG-GCU | AUA-GAG | AUA-GAC | GCC-GAG | GUG-GAG | GAG-AUA |
|  |  |  | 1 | 0.76563 | 0.60938 | 0.59375 | 0.53125 | 0.53125 | 0.5 | 0.48438 | 0.46875 | 0.46875 |
|  |  | Tr | GAG-GAG | GAG-AAG | AUA-GAG | GAG-AUA | AAG-GAG | GAG-GCU | AAG-CUC | GAC-GAG | AAG-AAG | GUU-GAG |
|  |  |  | 1 | 0.83133 | 0.57831 | 0.55422 | 0.54217 | 0.53012 | 0.51807 | 0.49398 | 0.49398 | 0.45783 |
|  |  | WG | GAG-GAG | GAG-AAG | GAG-GCU | GAG-CUA | AUA-GAG | GUU-GAG | GAG-AGG | GGC-GAG | GAG-AUA | GAG-CUG |
|  |  |  | 1 | 0.69908 | 0.60401 | 0.44701 | 0.4444 | 0.42782 | 0.41867 | 0.41474 | 0.41125 | 0.40951 |
| 28 | Ignicoccushospitalis KIN4/I, DSM 18386 | AAM | GAG-GAG | GAG-AGG | AAG-GAG | AAG-AAG | GAG-AAG | GAG-CUC | GUG-GAG | GAG-GCC | AAG-AGG | UUG-AAG |
|  |  |  | 1 | 0.87195 | 0.85976 | 0.78659 | 0.75 | 0.7439 | 0.73171 | 0.68902 | 0.67683 | 0.66463 |
|  |  | CM | GAG-GAG | AAG-GAG | GAG-CUG | GUG-GUG | GAG-GCC | GAG-AGG | GUG-GAG | AAG-GAC | GAG-CUC | AAG-AGG |
|  |  |  | 1 | 0.89091 | 0.74545 | 0.69091 | 0.67273 | 0.65455 | 0.63636 | 0.63636 | 0.61818 | 0.6 |
|  |  | EPC | GAG-GAG | AAG-GAG | GAG-AAG | GAG-AGG | AAG-AAG | AAG-AGG | GUG-GAG | GAG-GCC | AAG-UAC | AAG-GAC |
|  |  |  | 1 | 0.89404 | 0.82119 | 0.76821 | 0.72185 | 0.65563 | 0.63576 | 0.60927 | 0.5894 | 0.57616 |
|  |  | NM | GAG-GAG | GAG-AGG | GUG-GAG | AAG-GAG | AAG-AGG | GAG-CUG | GAC-GAG | AAG-AAG | GAG-GCG | GAG-CUC |
|  |  |  | 1 | 0.61616 | 0.60606 | 0.55556 | 0.48485 | 0.47475 | 0.45455 | 0.45455 | 0.44444 | 0.44444 |
|  |  | Tr | GAG-GAG | AAG-GAG | AAG-AAG | GAG-AAG | GAG-AGG | GAA-GAG | GAG-CUG | AAG-GAC | AAG-AGG | GUG-GAG |
|  |  |  | 1 | 0.80208 | 0.79167 | 0.6875 | 0.57292 | 0.55208 | 0.54167 | 0.52083 | 0.51042 | 0.48958 |
|  |  | WG | GAG-GAG | AAG-GAG | AAG-AAG | GAG-AGG | AAG-AGG | GAG-AAG | GUG-GAG | GAG-CUC | GAG-GCC | GAG-CUG |
|  |  |  | 1 | 0.77585 | 0.71614 | 0.68888 | 0.64734 | 0.63609 | 0.56556 | 0.5556 | 0.53743 | 0.51969 |
| 29 | Ignisphaeraaggregans AQ1.S1, DSM 17230 | AAM | AUA-GAU | GCU-AUA | GAU-AUA | AUA-GAG | AUA-GCU | UAU-AUA | UAU-AGA | GAU-AGA | AUA-GUU | AUA-UAU |
|  |  |  | 1 | 0.8374 | 0.77236 | 0.7561 | 0.71545 | 0.59756 | 0.58943 | 0.57317 | 0.56504 | 0.54878 |
|  |  | CM | AUA-GAU | GAU-AUA | UAU-AUA | GCU-AUA | UAU-AGA | AUA-GCU | AGA-GAU | AUA-GAG | GAU-AGA | GUU-AUA |
|  |  |  | 1 | 0.84524 | 0.77381 | 0.75 | 0.72619 | 0.70833 | 0.67262 | 0.66667 | 0.60119 | 0.58929 |
|  |  | EPC | AUA-GAU | GAU-AUA | AUA-GCU | GCU-AUA | AUA-GGU | AUA-GAG | AGA-UAU | UAU-AUA | AUA-GUU | GGU-AUA |
|  |  |  | 1 | 0.99115 | 0.99115 | 0.97345 | 0.83186 | 0.79646 | 0.79646 | 0.78761 | 0.74336 | 0.68142 |
|  |  | NM | AUA-GAU | AUA-GCU | AUA-GAG | GCU-AUA | UAU-AGA | AGA-GAU | GAU-AUA | GUU-AUA | GGU-AUA | GAU-AGA |
|  |  |  | 1 | 0.7619 | 0.7619 | 0.67857 | 0.66667 | 0.66667 | 0.65476 | 0.63095 | 0.61905 | 0.59524 |
|  |  | Tr | AUA-GAU | GAU-AUA | AUA-GAG | UAU-AUA | AGA-GAU | UAU-AGA | AUA-GCU | GAU-AUU | GAA-GAA | AUA-AAG |
|  |  |  | 1 | 0.96296 | 0.80247 | 0.77778 | 0.7037 | 0.65432 | 0.65432 | 0.61728 | 0.61728 | 0.60494 |
|  |  | WG | AUA-GAU | GAU-AUA | AUA-GCU | AUA-GAG | GCU-AUA | UAU-AUA | UAU-AGA | AUA-UAU | AGA-UAU | AGA-GAU |
|  |  |  | 1 | 0.81398 | 0.75259 | 0.74408 | 0.73299 | 0.66531 | 0.57618 | 0.55547 | 0.52478 | 0.51664 |
| 30 | Metallosphaeracuprina Ar-4 | AAM | GAG-AUA | GAG-GAG | AUA-GCU | AUA-GAG | AAG-GAG | AAG-AUA | AUA-GUU | GGA-GAG | GUU-AAG | GAG-AAG |
|  |  |  | 1 | 0.93284 | 0.91045 | 0.83582 | 0.80597 | 0.79104 | 0.71642 | 0.70896 | 0.70149 | 0.67164 |
|  |  | CM | AUA-AUA | AUA-GGA | AUA-GCU | AAG-GAG | GGG-AUA | AAG-AUA | GAG-GAG | AUA-GUU | AUG-AUA | GAG-AGG |
|  |  |  | 1 | 0.98649 | 0.90541 | 0.87838 | 0.83784 | 0.81081 | 0.78378 | 0.75676 | 0.74324 | 0.71622 |
|  |  | EPC | AAG-GAG | GAG-AAG | GAG-GAG | GAG-AUA | AAG-AUA | AUA-AAG | AUA-GGA | AUA-GCU | GGA-GAG | GAU-AAG |
|  |  |  | 1 | 0.77848 | 0.77215 | 0.75316 | 0.68354 | 0.67722 | 0.60759 | 0.60759 | 0.60127 | 0.60127 |
|  |  | NM | GAG-GAG | GAG-AUA | AUA-GAG | GAG-AAG | AAG-GAG | GAG-UUC | AUA-AAG | AGA-GAG | AUA-AUA | AAG-GAA |
|  |  |  | 1 | 0.77049 | 0.68852 | 0.63934 | 0.62295 | 0.54098 | 0.52459 | 0.52459 | 0.5082 | 0.5082 |
|  |  | Tr | GAG-AUA | AAG-GAG | AAG-AAG | GAG-GAG | GAG-AAG | AUA-GAG | AUA-AAG | AAG-AUA | GAU-GAG | GAG-GAA |
|  |  |  | 1 | 1 | 1 | 0.98305 | 0.94915 | 0.94915 | 0.84746 | 0.84746 | 0.81356 | 0.76271 |
|  |  | WG | GAG-GAG | AAG-GAG | GAG-AUA | AAG-AUA | GAG-AAG | AUA-GAG | AUA-AUA | AUA-GCU | AUA-AAG | AAG-GAA |
|  |  |  | 1 | 0.95966 | 0.91621 | 0.80683 | 0.79674 | 0.76959 | 0.72614 | 0.71296 | 0.71296 | 0.70132 |
| 31 | Metallosphaerasedula DSM 5348 | AAM | GAG-GAG | AAG-GAG | AGG-GAG | AAG-GGA | GUG-GAG | GGA-AUA | GAG-GAA | GAG-AUA | GUG-AAG | GAG-AAG |
|  |  |  | 1 | 0.78537 | 0.73659 | 0.6439 | 0.60976 | 0.59512 | 0.59024 | 0.57073 | 0.55122 | 0.54634 |
|  |  | CM | GAG-GAG | AAG-GAG | GGG-AUA | AAG-GGA | AGG-AUA | GUG-GAG | GAG-AAG | AGG-GAG | GUG-GGA | GGA-GAG |
|  |  |  | 1 | 0.94286 | 0.87143 | 0.85714 | 0.84286 | 0.81429 | 0.81429 | 0.8 | 0.78571 | 0.75714 |
|  |  | EPC | AAG-GAG | GAG-GAG | GAG-AAG | AAG-GGA | AGG-GAG | AGG-GAA | GAG-AUA | GAG-AGG | GUG-GGA | GAG-GGA |
|  |  |  | 1 | 0.84314 | 0.71078 | 0.70588 | 0.69118 | 0.65196 | 0.64216 | 0.61765 | 0.61275 | 0.60294 |
|  |  | NM | GAG-GAG | AAG-GAG | GAG-AUA | GAG-AAG | AGG-GAG | GAG-AUG | AUA-GAG | GAG-GAA | AGG-GAA | AAG-GGA |
|  |  |  | 1 | 0.89706 | 0.75 | 0.75 | 0.66176 | 0.54412 | 0.54412 | 0.52941 | 0.52941 | 0.51471 |
|  |  | Tr | AAG-GAG | GAG-GAG | GAG-GAA | GAG-AAG | AGG-GAG | AAG-AAG | AAG-GAA | AAG-AGG | GUG-GAG | GAG-AUA |
|  |  |  | 1 | 0.92941 | 0.8 | 0.75294 | 0.75294 | 0.71765 | 0.67059 | 0.63529 | 0.58824 | 0.58824 |
|  |  | WG | GAG-GAG | AAG-GAG | AGG-GAG | GAG-AAG | GAG-AUA | GAG-GAA | AAG-GGA | AAG-GAA | GUG-GAG | AAG-AAG |
|  |  |  | 1 | 0.98671 | 0.80565 | 0.78793 | 0.67165 | 0.66888 | 0.65891 | 0.62514 | 0.62016 | 0.60465 |
| 32 | Methanobrevibacterruminantium M1 | AAM | GAA-GAA | AUU-GAA | AAG-GAA | GAU-GAA | AUU-GCA | GAA-GAG | AUU-GAU | GAU-GCA | GUU-GAA | GCU-GAA |
|  |  |  | 1 | 0.97619 | 0.97619 | 0.875 | 0.85119 | 0.80952 | 0.72024 | 0.67857 | 0.66071 | 0.6369 |
|  |  | CM | AAG-GAA | AUU-GAA | GAA-AAG | AUU-GCA | GAC-AUU | GAU-GAG | GAU-AAG | GAU-GAA | UUU-GAA | GAA-GAA |
|  |  |  | 1 | 0.83117 | 0.77922 | 0.75325 | 0.68831 | 0.67532 | 0.67532 | 0.61039 | 0.5974 | 0.57143 |
|  |  | EPC | GAA-GAA | AUU-GAA | AAG-GAA | GAU-GAA | AUU-GCA | GAA-GCU | GAA-GAG | GCU-GAA | GAA-GAU | GAA-AAC |
|  |  |  | 1 | 0.9697 | 0.83636 | 0.80606 | 0.8 | 0.73333 | 0.69697 | 0.65455 | 0.6303 | 0.62424 |
|  |  | NM | AAG-GAA | AUU-GAA | GAU-GAA | GAA-GAG | AUU-GCA | GAU-AAG | UUU-GAA | AUU-GAU | GAA-AAG | GUU-GAA |
|  |  |  | 1 | 0.91045 | 0.89552 | 0.89552 | 0.85075 | 0.71642 | 0.67164 | 0.65672 | 0.64179 | 0.62687 |
|  |  | Tr | AAG-GAA | AUU-GAA | GAU-GAA | GAA-AAG | GAA-GAG | GAU-GAU | GAA-GAA | GAA-AAU | GAA-AAA | UUU-GAA |
|  |  |  | 1 | 0.96 | 0.94 | 0.89 | 0.81 | 0.78 | 0.72 | 0.68 | 0.62 | 0.59 |
|  |  | WG | GAU-GAA | AUU-GAA | GAU-GAU | GAA-GAA | AAG-GAA | GAA-GAG | AAU-GAA | GAA-AAG | GAA-AAU | GAA-AAA |
|  |  |  | 1 | 0.96823 | 0.91144 | 0.90786 | 0.89952 | 0.7633 | 0.75774 | 0.72399 | 0.71088 | 0.69936 |
| 33 | Methanocaldococcusfervens AG86 | AAM | AAA-GAA | UUA-AAA | GAA-AAA | AAA-AAA | AUA-AAA | AAA-GAU | GUU-AAA | GUU-UUA | AAA-GAG | GAU-UUA |
|  |  |  | 1 | 0.9604 | 0.86139 | 0.74752 | 0.74257 | 0.71287 | 0.69802 | 0.68812 | 0.68317 | 0.65842 |
|  |  | CM | UUA-AAA | AUA-AAA | AAA-AAA | AAA-GAA | GAU-UUA | AAA-GAU | GAA-AAA | AAA-AAU | GAU-GUU | GUU-GUU |
|  |  |  | 1 | 0.90526 | 0.90526 | 0.8 | 0.74737 | 0.72632 | 0.70526 | 0.69474 | 0.67368 | 0.66316 |
|  |  | EPC | GAA-AAA | AAA-GAA | UUA-AAA | AAA-AAA | AUA-AAA | AAA-GAU | GUU-GAA | GAU-GAA | GAA-GUU | GUU-AAA |
|  |  |  | 1 | 1 | 0.95 | 0.895 | 0.845 | 0.745 | 0.725 | 0.725 | 0.725 | 0.705 |
|  |  | NM | UUA-AAA | AAA-GAA | AAA-AAA | AUA-AAA | AAA-GAU | GAA-AAA | GUU-UUA | GAU-GAA | GAA-GAA | AAA-GAG |
|  |  |  | 1 | 0.96296 | 0.85185 | 0.83951 | 0.83951 | 0.82716 | 0.80247 | 0.79012 | 0.77778 | 0.76543 |
|  |  | Tr | GAA-AAA | UUA-AAA | AAA-GAA | AAA-AAA | AAA-GAU | GAA-GAA | AUA-AAA | AUU-GAA | AAA-AUU | AAA-GAG |
|  |  |  | 1 | 0.97674 | 0.95349 | 0.80233 | 0.74419 | 0.7093 | 0.7093 | 0.66279 | 0.66279 | 0.61628 |
|  |  | WG | UUA-AAA | AAA-AAA | AAA-GAA | GAA-AAA | AUA-AAA | AAA-GAU | AAA-AAU | GAU-GAA | GAA-GAA | AUU-GAA |
|  |  |  | 1 | 0.99133 | 0.98542 | 0.90898 | 0.79905 | 0.70213 | 0.69622 | 0.66115 | 0.6383 | 0.62845 |
| 34 | Methanocaldococcusinfernus ME | AAM | AAA-GAG | GAA-GAG | AAG-GUU | UUA-AAA | GCU-AAG | AUA-GAG | GAG-AAG | GUU-AAA | GAG-GUU | GAG-AUA |
|  |  |  | 1 | 0.80808 | 0.79293 | 0.74747 | 0.71212 | 0.71212 | 0.67172 | 0.63131 | 0.62626 | 0.62626 |
|  |  | CM | AAA-GAG | GAA-GAG | UUA-GAG | GUU-AAA | AAG-GUU | GCU-AAG | GAG-GUU | UUA-AAA | UUA-GAU | UUA-AAG |
|  |  |  | 1 | 0.875 | 0.78409 | 0.73864 | 0.72727 | 0.71591 | 0.70455 | 0.69318 | 0.64773 | 0.64773 |
|  |  | EPC | AAA-GAG | AAG-GUU | GAA-GAG | UUA-GAG | GAG-AAG | GAG-GUU | AUA-AAG | UUA-AAA | GUU-AAA | GCU-AAG |
|  |  |  | 1 | 0.84925 | 0.76884 | 0.74372 | 0.73869 | 0.68844 | 0.66332 | 0.63317 | 0.61809 | 0.60804 |
|  |  | NM | AAA-GAG | GAA-GAG | GCU-AAG | UUA-GAG | UUA-AAA | GAG-AAG | AUA-AAG | AAG-GUU | AAG-AUA | GUU-GAU |
|  |  |  | 1 | 0.72381 | 0.64762 | 0.59048 | 0.59048 | 0.59048 | 0.58095 | 0.57143 | 0.55238 | 0.54286 |
|  |  | Tr | GAA-GAG | AAA-GAG | AAG-GUU | AAG-AAG | UUA-AAA | GAG-AAG | AAA-AAA | UUA-AAG | GUU-AAG | AAG-AUA |
|  |  |  | 1 | 0.93827 | 0.7284 | 0.7284 | 0.69136 | 0.69136 | 0.67901 | 0.66667 | 0.64198 | 0.64198 |
|  |  | WG | AAA-GAG | GAA-GAG | UUA-AAA | AAG-GUU | GAG-AAG | UUA-GAG | UUA-AAG | GCU-AAG | AAA-AAA | AUA-GAG |
|  |  |  | 1 | 0.86323 | 0.81779 | 0.73829 | 0.7132 | 0.6815 | 0.65878 | 0.63086 | 0.61903 | 0.61571 |
| 35 | Methanocaldococcusjannaschii DSM 2661 | AAM | UUA-AAA | AAA-GAA | AUA-AAA | GUU-AAA | AAA-GAG | AAA-AAA | GAU-UUA | GAA-AAA | AAA-GAU | GGA-GUU |
|  |  |  | 1 | 0.89063 | 0.81771 | 0.8125 | 0.80729 | 0.78646 | 0.78125 | 0.73438 | 0.71354 | 0.70833 |
|  |  | CM | UUA-AAA | AAA-AAA | GAA-AAA | AAA-GAA | GUU-AAA | GAU-UUA | AUA-AAA | AAA-GAU | GUU-GUU | AUU-AAA |
|  |  |  | 1 | 0.8 | 0.74 | 0.74 | 0.73 | 0.73 | 0.7 | 0.69 | 0.67 | 0.66 |
|  |  | EPC | AAA-GAA | AAA-AAA | UUA-AAA | GAA-AAA | AUA-AAA | AAA-GAG | GUU-AAA | GAU-AAA | GAA-GAA | GUU-GUU |
|  |  |  | 1 | 0.97938 | 0.95361 | 0.87629 | 0.86082 | 0.79897 | 0.76289 | 0.74742 | 0.74227 | 0.73711 |
|  |  | NM | UUA-AAA | AAA-GAA | AAA-GAU | AAA-AAA | AUA-AAA | GUU-AAA | GAA-GAA | AUU-GAA | GUU-GAU | GAU-GAA |
|  |  |  | 1 | 0.96875 | 0.78125 | 0.76042 | 0.73958 | 0.71875 | 0.70833 | 0.69792 | 0.6875 | 0.6875 |
|  |  | Tr | UUA-AAA | AAA-GAA | AUA-AAA | AAA-GAG | AAA-AAA | GAA-AAA | GAA-GAA | AAA-AUU | AAA-GAU | AUU-GAA |
|  |  |  | 1 | 1 | 0.91765 | 0.89412 | 0.84706 | 0.83529 | 0.82353 | 0.81176 | 0.77647 | 0.76471 |
|  |  | WG | AAA-GAA | UUA-AAA | AAA-AAA | AUA-AAA | GAA-AAA | AAA-AAU | AAA-GAU | AAA-GAG | GAA-GAA | AUU-GAA |
|  |  |  | 1 | 0.98859 | 0.96688 | 0.85683 | 0.85241 | 0.71402 | 0.68347 | 0.66286 | 0.65329 | 0.64299 |
| 36 | Methanocaldococcusvulcanius DSM 12094 | AAM | AUA-AAA | AAA-AAA | UUA-AAA | AAA-GAA | GAA-AAA | AAA-AUA | GAA-GAA | AAA-GAU | AAA-GAG | GUU-AAA |
|  |  |  | 1 | 1 | 0.92308 | 0.9188 | 0.86325 | 0.64957 | 0.59829 | 0.58974 | 0.58974 | 0.56838 |
|  |  | CM | AAA-AAA | UUA-AAA | AUA-AAA | GAA-AAA | AAA-AAU | AAA-AUA | AAA-GAA | AAA-GAU | GAA-AAU | GAU-AAA |
|  |  |  | 1 | 0.96078 | 0.96078 | 0.92157 | 0.7549 | 0.73529 | 0.68627 | 0.66667 | 0.64706 | 0.60784 |
|  |  | EPC | GAA-AAA | AAA-GAA | AAA-AAA | UUA-AAA | AUA-AAA | GAA-GAA | AAA-AUA | GUU-AAA | GAA-GUU | AAA-GUU |
|  |  |  | 1 | 0.90706 | 0.87732 | 0.83643 | 0.73606 | 0.62825 | 0.56506 | 0.53903 | 0.52788 | 0.52788 |
|  |  | NM | AAA-AAA | UUA-AAA | AAA-GAA | AUA-AAA | GAA-AAA | GAA-GAA | AAA-GAU | GAU-GAA | AAA-AUA | AUU-GAA |
|  |  |  | 1 | 0.92553 | 0.85106 | 0.82979 | 0.78723 | 0.67021 | 0.65957 | 0.62766 | 0.61702 | 0.59574 |
|  |  | Tr | GAA-AAA | UUA-AAA | AAA-AAA | AUA-AAA | AAA-GAA | AAA-AUA | GAA-GAA | AAA-GAU | AAA-AAU | AAA-GAG |
|  |  |  | 1 | 0.97183 | 0.90141 | 0.82394 | 0.80282 | 0.71831 | 0.6831 | 0.64085 | 0.60563 | 0.59859 |
|  |  | WG | AAA-AAA | UUA-AAA | AUA-AAA | GAA-AAA | AAA-GAA | AAA-AAU | AAA-AUA | GAA-GAA | AAA-GAU | AAA-GAG |
|  |  |  | 1 | 0.91306 | 0.86293 | 0.85736 | 0.84189 | 0.65285 | 0.64697 | 0.59932 | 0.57519 | 0.50371 |
| 37 | Methanococcoidesburtonii DSM 6242 | AAM | CUU-GAA | GAA-CUU | CUU-GCA | GAU-AUU | GAG-AUC | GAU-GCA | CUU-GAU | GAA-GAA | GGU-GCA | AAG-AUC |
|  |  |  | 1 | 0.97778 | 0.8963 | 0.86667 | 0.85185 | 0.83704 | 0.83704 | 0.78519 | 0.76296 | 0.74074 |
|  |  | CM | GAA-GAU | GAU-GCA | GAA-GAA | GAA-AAG | GAG-AUA | CUU-GCA | CUU-GAA | AUU-GAA | GAU-GUU | AUG-GCA |
|  |  |  | 1 | 0.89091 | 0.89091 | 0.83636 | 0.81818 | 0.81818 | 0.8 | 0.8 | 0.76364 | 0.76364 |
|  |  | EPC | CUU-GCA | GAU-GAA | AAG-AUC | GCA-AAG | GAG-AUC | GAU-GUU | CUU-GAA | GCA-GCA | GAU-GCA | GAA-GAA |
|  |  |  | 1 | 0.92126 | 0.87402 | 0.86614 | 0.85827 | 0.82677 | 0.8189 | 0.81102 | 0.7874 | 0.7874 |
|  |  | NM | GAG-AUC | CUU-GAG | AAG-AUC | GAU-GAG | AUC-GAU | GAA-CUU | CUU-GAU | CUU-GAA | GGU-GCA | GAG-AUA |
|  |  |  | 1 | 0.91489 | 0.89362 | 0.87234 | 0.85106 | 0.82979 | 0.82979 | 0.78723 | 0.7234 | 0.7234 |
|  |  | Tr | GAU-GAU | GAU-GAA | CUU-GAA | GAG-AUC | GAA-CUU | GAU-GAG | GAA-AAA | AAA-GAA | GAA-GAU | GAA-GAA |
|  |  |  | 1 | 1 | 0.96825 | 0.93651 | 0.92063 | 0.90476 | 0.90476 | 0.87302 | 0.85714 | 0.85714 |
|  |  | WG | GAU-GAA | GAA-GAA | GAU-GAU | GAA-GAU | CUU-GAA | GAA-AAA | GAU-AUU | GAA-CUU | AAA-GAA | CUU-GCA |
|  |  |  | 1 | 0.96382 | 0.88849 | 0.87663 | 0.86892 | 0.80842 | 0.80308 | 0.79004 | 0.77639 | 0.77165 |
| 38 | Methanococcusaeolicus Nankai-3 | AAM | AAA-GAA | AAA-AUA | UUA-AAA | AAA-AAU | GAA-AAA | AUA-AAA | AAA-AAA | AAU-AAA | GAA-AUA | AAA-UUA |
|  |  |  | 1 | 0.93333 | 0.9098 | 0.80392 | 0.79608 | 0.78039 | 0.76863 | 0.71765 | 0.71373 | 0.71373 |
|  |  | CM | UUA-AAA | AAA-AAA | AAA-AAU | AAA-GAA | AAA-AUA | GAU-AAA | GAA-AAA | AAU-AAA | UUA-AAU | AAA-UUA |
|  |  |  | 1 | 0.98438 | 0.96875 | 0.88281 | 0.8125 | 0.79688 | 0.79688 | 0.78125 | 0.75781 | 0.71875 |
|  |  | EPC | GAA-AAA | AAA-GAA | AAA-AAA | AAA-AUA | UUA-AAA | AAA-AAU | AAA-UUA | AUA-AAA | AAU-AAA | AUU-GAA |
|  |  |  | 1 | 0.90879 | 0.90228 | 0.89251 | 0.84365 | 0.77524 | 0.7329 | 0.70684 | 0.6645 | 0.61564 |
|  |  | NM | AAA-GAA | UUA-AAA | AAA-AUA | GAA-AAA | GAA-AUA | AAA-AAA | AUA-AAA | AAU-AAA | AAA-AAU | AAA-UUA |
|  |  |  | 1 | 0.78205 | 0.78205 | 0.73077 | 0.71795 | 0.67308 | 0.66026 | 0.64744 | 0.60897 | 0.53846 |
|  |  | Tr | AAA-AAA | AAA-AUA | GAA-AAA | AAA-GAA | UUA-AAA | AAU-AAA | AUA-AAA | AAA-UUA | GAA-GAA | AAA-AAU |
|  |  |  | 1 | 0.9901 | 0.9604 | 0.9604 | 0.89109 | 0.74257 | 0.73267 | 0.73267 | 0.71287 | 0.71287 |
|  |  | WG | AAA-AUA | AAA-AAA | UUA-AAA | AAA-GAA | AAA-AAU | GAA-AAA | AAU-AAA | AUA-AAA | AAA-UUA | GAA-AAU |
|  |  |  | 1 | 0.99275 | 0.91035 | 0.90771 | 0.90639 | 0.87508 | 0.83784 | 0.75643 | 0.68919 | 0.64272 |
| 39 | Methanocorpusculumlabreanum Z | AAM | GAG-AUC | AUC-AUC | GCA-AAA | GCG-AUC | AUC-GGC | ACG-AUC | AUC-GUC | AUC-GCC | GAA-AAA | AAG-AUC |
|  |  |  | 1 | 0.74699 | 0.64458 | 0.61446 | 0.54819 | 0.54819 | 0.54217 | 0.53614 | 0.53012 | 0.53012 |
|  |  | CM | CUG-AUC | GCG-AUC | AUG-AUC | AUC-GGC | GAG-AUC | CUU-GCC | AUC-GUC | GCA-AAA | AUC-AUC | GGG-AUC |
|  |  |  | 1 | 0.89831 | 0.88136 | 0.88136 | 0.76271 | 0.74576 | 0.69492 | 0.67797 | 0.66102 | 0.64407 |
|  |  | EPC | GAG-AUC | AUC-AUC | GCA-AAA | AAG-AUC | AUC-GUC | AUC-GAA | GGA-AAA | AUC-GCC | GAA-GCA | GAA-AAA |
|  |  |  | 1 | 0.85385 | 0.76923 | 0.76154 | 0.73077 | 0.73077 | 0.68462 | 0.67692 | 0.66923 | 0.65385 |
|  |  | NM | GAG-AUC | AUC-AUC | AAG-AUC | AUC-GGC | GCG-AUC | GCA-AAA | AUC-GAA | AUC-GAC | AUC-AAA | ACG-AUC |
|  |  |  | 1 | 0.81667 | 0.81667 | 0.78333 | 0.75 | 0.71667 | 0.71667 | 0.65 | 0.61667 | 0.61667 |
|  |  | Tr | GAG-AUC | AAG-AUC | AUC-AUC | GAA-AAA | AAA-GAG | GAA-GAG | AUC-AAA | GAG-GAG | GAU-AUC | AAA-AAA |
|  |  |  | 1 | 0.76056 | 0.74648 | 0.70423 | 0.70423 | 0.69014 | 0.67606 | 0.64789 | 0.61972 | 0.59155 |
|  |  | WG | GAG-AUC | AUC-AUC | GAA-AAA | CUG-AUC | GCA-AAA | AAG-AUC | AAA-AAA | GAA-GAG | AUC-GAA | GGA-AAA |
|  |  |  | 1 | 0.87557 | 0.73947 | 0.6954 | 0.68503 | 0.65457 | 0.62152 | 0.61309 | 0.60078 | 0.59883 |
| 40 | Methanoculleusmarisnigri JR1, DSM 1498 | AAM | GAG-AUC | GCG-CUC | AUC-GAG | GCC-GUC | GUC-GUC | GCG-GCG | GAG-GCG | CUC-GCC | GAG-GAG | AUC-GUC |
|  |  |  | 1 | 0.95089 | 0.90625 | 0.86161 | 0.83929 | 0.83482 | 0.83036 | 0.83036 | 0.81696 | 0.79911 |
|  |  | CM | CUC-CUC | GAG-AUC | GCC-GUC | GCG-CUC | CUC-GCC | GCG-GCG | GUC-GCC | CUC-GUC | CUC-GAC | GCC-GGG |
|  |  |  | 1 | 0.88 | 0.864 | 0.816 | 0.816 | 0.792 | 0.776 | 0.776 | 0.776 | 0.744 |
|  |  | EPC | GAG-AUC | GCG-CUC | GUC-GUC | AUC-GAG | GAG-GAG | GUC-GAG | CUC-GCC | AUC-GGC | AUC-GUC | CUC-GGC |
|  |  |  | 1 | 0.88306 | 0.81855 | 0.77419 | 0.7621 | 0.74597 | 0.74597 | 0.74194 | 0.7379 | 0.71774 |
|  |  | NM | GAG-AUC | AUC-GUC | AUC-GAG | CUC-GCG | GCG-CUC | CUC-GCC | GUC-GUC | CUC-CUC | GCC-GUC | AUC-GAC |
|  |  |  | 1 | 0.95789 | 0.91579 | 0.81053 | 0.8 | 0.8 | 0.78947 | 0.77895 | 0.75789 | 0.74737 |
|  |  | Tr | AUC-GAG | GAG-AUC | GAG-GAG | GAG-CGG | CUC-GAG | GAA-CUC | GUC-GUC | AUC-AUC | GCG-CUC | GUC-AUC |
|  |  |  | 1 | 0.90441 | 0.64706 | 0.63235 | 0.625 | 0.60294 | 0.59559 | 0.58824 | 0.55882 | 0.54412 |
|  |  | WG | GAG-AUC | CUC-CUC | AUC-GAG | GAG-GAG | GUC-GUC | GCG-CUC | AUC-GUC | CUC-GCC | GCC-GUC | CUC-GUC |
|  |  |  | 1 | 0.84521 | 0.80788 | 0.80308 | 0.77911 | 0.77911 | 0.77534 | 0.77021 | 0.72363 | 0.69863 |
| 41 | Methanofollisliminatans GKZPZ, DSM 4140 | AAM | AUC-GCC | GCC-GCC | GAG-AUC | GCG-AUC | GCG-GCG | GCC-CUC | GGG-AUC | GUC-GCC | CUC-GCC | GAC-GCC |
|  |  |  | 1 | 0.95941 | 0.95203 | 0.88561 | 0.73801 | 0.73063 | 0.72325 | 0.65683 | 0.65314 | 0.64945 |
|  |  | CM | AUC-GCC | GCC-GCC | CUC-GCC | GCC-CUC | CUC-CUC | GUC-GCC | GCG-GCG | GCG-AUC | GCC-GGG | AUC-GUC |
|  |  |  | 1 | 0.96429 | 0.83929 | 0.80357 | 0.76786 | 0.75893 | 0.75 | 0.74107 | 0.71429 | 0.70536 |
|  |  | EPC | GAG-AUC | AUC-GCC | GCG-AUC | GGG-AUC | CUC-GCC | GCC-GCC | GAC-GCC | GCG-GCG | AUC-GUC | GCC-CUC |
|  |  |  | 1 | 1 | 0.87143 | 0.85714 | 0.79643 | 0.76786 | 0.76786 | 0.75 | 0.75 | 0.74643 |
|  |  | NM | GAG-AUC | AUC-GCC | CUC-GCC | GUC-GCC | GCG-AUC | GCC-GCC | GCC-CUC | GAC-GCC | GAG-GCG | GAG-GAG |
|  |  |  | 1 | 0.89189 | 0.88288 | 0.85586 | 0.79279 | 0.79279 | 0.78378 | 0.7027 | 0.69369 | 0.68468 |
|  |  | Tr | GAG-AUC | GAG-GAG | AUC-GCC | AUC-GUC | CUG-AUC | AUC-GAG | GAG-GCG | GCC-CUC | GCG-AUC | GGG-AUC |
|  |  |  | 1 | 0.66667 | 0.64744 | 0.61538 | 0.60256 | 0.59615 | 0.57051 | 0.54487 | 0.53205 | 0.52564 |
|  |  | WG | GAG-AUC | AUC-GCC | GCC-GCC | GCC-CUC | GCG-AUC | CUC-GCC | GAG-GAG | CUC-CUC | AUC-GUC | AUC-GAG |
|  |  |  | 1 | 0.98474 | 0.88187 | 0.81593 | 0.79365 | 0.78083 | 0.75977 | 0.72589 | 0.7149 | 0.70757 |
| 42 | Methanomassiliicoccusluminyensis B10 | AAM | AUC-GCC | GCC-AAG | CUG-GAG | GAG-CUG | GAC-GAG | GGC-AUC | GAG-GAG | CUG-GAC | AAG-GCC | GUG-GAG |
|  |  |  | 1 | 0.95455 | 0.93182 | 0.86364 | 0.78788 | 0.7803 | 0.7803 | 0.77652 | 0.77652 | 0.76515 |
|  |  | CM | CUG-GCC | CUG-GUG | UUC-CUG | GAG-CUG | CUG-GGC | AUC-GCC | GCG-GUG | GCC-CUG | CUG-GAC | CUG-UUC |
|  |  |  | 1 | 0.98319 | 0.89916 | 0.89916 | 0.88235 | 0.88235 | 0.87395 | 0.86555 | 0.86555 | 0.84034 |
|  |  | EPC | CUG-GAG | AUC-GCC | GAG-GAG | GAG-AAG | CUG-CUG | CUG-GCC | GCC-AUC | GCC-AAG | GAG-CUG | GAG-GUC |
|  |  |  | 1 | 0.99519 | 0.99038 | 0.97115 | 0.97115 | 0.96635 | 0.95192 | 0.92788 | 0.92308 | 0.88942 |
|  |  | NM | CUG-GAC | GAG-CUG | GUC-AUC | AUC-GCC | GAG-GAG | GGC-AUC | GCC-AAG | CUG-GAG | AUC-GAG | GCC-GAG |
|  |  |  | 1 | 0.96341 | 0.92683 | 0.90244 | 0.89024 | 0.84146 | 0.84146 | 0.84146 | 0.82927 | 0.78049 |
|  |  | Tr | GAG-GAG | GAG-CUG | GAG-AAG | CUG-GAG | AAG-GAG | GAC-GAG | CUG-GAC | AUC-GAG | GCC-AAG | AAG-AAG |
|  |  |  | 1 | 1 | 0.98261 | 0.94783 | 0.92174 | 0.89565 | 0.86087 | 0.84348 | 0.83478 | 0.83478 |
|  |  | WG | CUG-GAG | GAG-GAG | GAG-CUG | GAG-AAG | CUG-GAC | AUC-GCC | GCC-AAG | CUG-GCC | CUG-GUG | GCC-AUC |
|  |  |  | 1 | 0.9707 | 0.97032 | 0.87519 | 0.87215 | 0.84932 | 0.84779 | 0.82915 | 0.81963 | 0.80518 |
| 43 | Methanoplanuspetrolearius SEBR 4847, DSM 11571 | AAM | GAG-AUC | AAG-AUC | GAA-GAG | GAC-GAG | GAU-CUC | GGA-AAG | GAU-AUC | AAG-GAG | GAG-AUG | CUU-GCA |
|  |  |  | 1 | 0.67742 | 0.66129 | 0.61828 | 0.59677 | 0.57527 | 0.57527 | 0.56989 | 0.53763 | 0.53763 |
|  |  | CM | CUU-GCA | GAG-AUC | AUC-GGC | UUC-CUG | AUG-AUC | GCC-GGA | GAA-AAA | AUA-AUC | AUU-GCA | UUU-GCA |
|  |  |  | 1 | 0.96491 | 0.85965 | 0.84211 | 0.80702 | 0.77193 | 0.77193 | 0.75439 | 0.73684 | 0.70175 |
|  |  | EPC | GAG-AUC | GAG-AAG | AAG-AUC | AUC-GAG | AAG-AAG | GAA-GAG | CUU-GCA | AAG-GAG | GAU-AUC | AUC-GAC |
|  |  |  | 1 | 0.61538 | 0.59615 | 0.56923 | 0.53462 | 0.52308 | 0.50769 | 0.47692 | 0.46154 | 0.46154 |
|  |  | NM | GAG-AUC | GAA-GAG | AUC-GAU | GAG-GAG | AAG-AUC | GAU-AUC | GAA-AAG | AUC-GAG | CUU-GAA | AUC-GGC |
|  |  |  | 1 | 0.65909 | 0.625 | 0.57955 | 0.55682 | 0.53409 | 0.52273 | 0.5 | 0.48864 | 0.48864 |
|  |  | Tr | GAG-AUC | GAA-GAG | AAG-AUC | AAA-AAA | GAA-AAA | GAA-AAG | AUA-AAA | GAU-AUC | AUC-GAG | CUU-GAA |
|  |  |  | 1 | 0.91489 | 0.76596 | 0.7234 | 0.71277 | 0.67021 | 0.6383 | 0.60638 | 0.60638 | 0.58511 |
|  |  | WG | GAG-AUC | GAA-GAG | GAA-AAA | AAG-AUC | GAU-AUC | GAA-GAA | GAA-AAG | CUU-GCA | AAA-AAA | GAG-GAG |
|  |  |  | 1 | 0.67503 | 0.64413 | 0.60414 | 0.54053 | 0.52744 | 0.51763 | 0.49437 | 0.49291 | 0.49182 |
| 44 | Methanopyruskandleri AV19 | AAM | GAG-GAG | GUC-GAG | GAC-GAG | GUG-GAG | GAG-CUG | GAG-CUC | GAG-GUG | GAG-GAC | AUC-GAG | GAG-GUC |
|  |  |  | 1 | 0.58431 | 0.58431 | 0.57647 | 0.54118 | 0.54118 | 0.53725 | 0.53333 | 0.53333 | 0.51765 |
|  |  | CM | GAG-GAG | GAC-GAG | GAG-GUC | GAG-CUC | GUG-GAG | GUG-AUC | GAG-GAC | GAG-GCC | GCC-GAG | GAC-GUC |
|  |  |  | 1 | 0.58333 | 0.4375 | 0.41667 | 0.39583 | 0.39583 | 0.38542 | 0.375 | 0.36458 | 0.35417 |
|  |  | EPC | GAG-GAG | GUC-GAG | GAG-AUC | AUC-GAG | GAG-GAC | GAC-GAG | GCC-GAG | GAG-GUC | GAG-CUC | GAC-GUG |
|  |  |  | 1 | 0.66207 | 0.58621 | 0.56897 | 0.54138 | 0.49655 | 0.44828 | 0.44483 | 0.43448 | 0.43103 |
|  |  | NM | GAG-GAG | GAG-AUC | GAC-GAG | CUC-GAG | GCC-GAG | AUC-GAG | GUC-GAG | GAG-UUC | GAG-GAA | GAG-CUG |
|  |  |  | 1 | 0.57759 | 0.57759 | 0.49138 | 0.47414 | 0.47414 | 0.46552 | 0.4569 | 0.42241 | 0.41379 |
|  |  | Tr | GAG-GAG | GAG-AUC | GAC-GAG | GAG-AAG | GAG-CUC | AAG-GAG | GUG-GAG | GAG-GUC | GAG-CUG | CUC-GAG |
|  |  |  | 1 | 0.51095 | 0.47445 | 0.44526 | 0.41606 | 0.41606 | 0.39416 | 0.37956 | 0.37956 | 0.37226 |
|  |  | WG | GAG-GAG | GUC-GAG | GAC-GAG | GAG-CUC | GAG-AUC | GAG-GAC | GUG-GAG | CUC-GAG | GAG-CUG | AUC-GAG |
|  |  |  | 1 | 0.57652 | 0.53613 | 0.52635 | 0.51751 | 0.51089 | 0.47807 | 0.47775 | 0.47586 | 0.4705 |
| 45 | Methanosarcinaacetivorans C2A | AAM | GAA-AAA | GAA-GAA | CUU-GAA | CUU-GCA | AAA-GAA | GGA-GAA | GGA-AAA | GAA-GCC | GGG-CUU | CUU-GCC |
|  |  |  | 1 | 0.97049 | 0.87869 | 0.72787 | 0.70164 | 0.6623 | 0.63934 | 0.61639 | 0.60984 | 0.60656 |
|  |  | CM | GAA-AAA | GAA-GAA | GGA-AAA | CUU-GCA | GGA-GAA | GGG-CUU | AAA-AAA | GAA-GCC | AAA-GAA | CUU-GAA |
|  |  |  | 1 | 0.83766 | 0.6039 | 0.57143 | 0.55844 | 0.55195 | 0.53896 | 0.53247 | 0.52597 | 0.51948 |
|  |  | EPC | GAA-GAA | GAA-AAA | CUU-GAA | CUU-GCA | GCA-AAA | GAA-GCC | GAA-AAG | AAA-GAA | GGA-AAA | GAA-GAG |
|  |  |  | 1 | 0.91429 | 0.7619 | 0.63175 | 0.62857 | 0.62857 | 0.61905 | 0.59365 | 0.57778 | 0.54286 |
|  |  | NM | GAA-GAA | GAA-AAA | AAA-GAA | CUU-GAA | AAA-AAA | GAA-GCC | GGA-AAA | GCA-AAA | GAA-GAC | GAA-CUU |
|  |  |  | 1 | 0.91209 | 0.71429 | 0.68132 | 0.62637 | 0.59341 | 0.53846 | 0.53846 | 0.52747 | 0.51648 |
|  |  | Tr | GAA-AAA | GAA-GAA | AAA-GAA | AAA-AAA | CUU-GAA | GAA-GAG | GAA-CUU | GAA-AAG | AAG-GAA | GGA-AAA |
|  |  |  | 1 | 0.97758 | 0.65022 | 0.57848 | 0.50224 | 0.4843 | 0.47085 | 0.46637 | 0.4574 | 0.44843 |
|  |  | WG | GAA-GAA | GAA-AAA | AAA-GAA | CUU-GAA | AAA-AAA | GGA-AAA | GAA-AAG | GAA-AAC | GAA-AAU | GAA-GAG |
|  |  |  | 1 | 0.95323 | 0.69414 | 0.63945 | 0.6255 | 0.55063 | 0.53102 | 0.51103 | 0.49067 | 0.47992 |
| 46 | Methanosarcinamazei Go1, DSM 3647 | AAM | GAA-GAA | GAA-AAA | CUU-GAA | AAA-GAA | CUU-GCA | GGG-CUU | GGA-AAA | GGA-GAA | AAA-AAA | GAA-GCC |
|  |  |  | 1 | 0.98785 | 0.91093 | 0.8502 | 0.77733 | 0.7247 | 0.7166 | 0.63563 | 0.62348 | 0.61538 |
|  |  | CM | GAA-AAA | GAA-GAA | CUU-GCA | CUU-GAA | GGA-GAA | AAA-GAA | GGG-CUU | GAA-AAG | AAA-AAA | GGA-AAA |
|  |  |  | 1 | 0.84466 | 0.81553 | 0.80583 | 0.67961 | 0.6699 | 0.65049 | 0.65049 | 0.65049 | 0.64078 |
|  |  | EPC | GAA-GAA | GAA-AAA | CUU-GAA | CUU-GCA | AAA-GAA | GAA-AAG | GCA-AAA | GGA-AAA | GGA-GAA | AUU-GCA |
|  |  |  | 1 | 0.99216 | 0.82745 | 0.73725 | 0.73725 | 0.71765 | 0.60784 | 0.59216 | 0.58431 | 0.56863 |
|  |  | NM | GAA-GAA | GAA-AAA | CUU-GAA | GGA-AAA | AAA-GAA | CUU-GCA | AAA-AAA | GAA-AAG | GGG-CUU | GCA-GAA |
|  |  |  | 1 | 0.77647 | 0.67059 | 0.6 | 0.6 | 0.55294 | 0.50588 | 0.49412 | 0.48235 | 0.47059 |
|  |  | Tr | GAA-GAA | GAA-AAA | AAA-GAA | AAA-AAA | CUU-GAA | AAA-AAG | GAA-GAG | GAA-AUU | GAA-AAG | GAA-CUU |
|  |  |  | 1 | 0.91411 | 0.80982 | 0.65644 | 0.63804 | 0.54601 | 0.53988 | 0.53374 | 0.52147 | 0.4908 |
|  |  | WG | GAA-GAA | GAA-AAA | AAA-GAA | CUU-GAA | AAA-AAA | GGA-AAA | GAA-GAG | GAA-AAG | CCU-GAA | GAA-AAU |
|  |  |  | 1 | 0.98059 | 0.77622 | 0.69221 | 0.63522 | 0.57357 | 0.49546 | 0.48661 | 0.47802 | 0.47679 |
| 47 | Methanothermobacterthermautotrophicus Delta H | AAM | GAG-AUA | CUU-GCA | GAG-GAG | GAU-GAG | AGG-GAG | GAG-GCC | CUU-GAA | GCA-GAG | GAG-GAC | GAA-CUC |
|  |  |  | 1 | 0.96552 | 0.93966 | 0.93103 | 0.92241 | 0.90517 | 0.85345 | 0.7931 | 0.76724 | 0.76724 |
|  |  | CM | GAG-GAG | AGG-GAG | CUU-GAG | GAG-GAC | CUC-AGG | GAU-GAG | GAC-CUC | AGG-AUA | CUU-GCA | CUC-AUA |
|  |  |  | 1 | 0.79688 | 0.78125 | 0.73438 | 0.6875 | 0.65625 | 0.65625 | 0.65625 | 0.625 | 0.625 |
|  |  | EPC | GAG-GAG | AGG-GAG | CUU-GAG | GAU-GAG | CUU-GCA | GAG-AGG | AUA-AGG | CUU-GAA | CUC-AUA | GUU-GAG |
|  |  |  | 1 | 0.68664 | 0.64977 | 0.6129 | 0.59908 | 0.58525 | 0.58065 | 0.56221 | 0.56221 | 0.5576 |
|  |  | NM | GAG-GAG | CUU-GAG | GUU-GAG | AGG-AUA | AGG-GAG | AUA-AGG | GAG-GCC | GAG-AUA | CUC-AGG | GAG-GUC |
|  |  |  | 1 | 0.74684 | 0.73418 | 0.70886 | 0.6962 | 0.67089 | 0.64557 | 0.64557 | 0.63291 | 0.56962 |
|  |  | Tr | GAG-GAG | GAG-AUA | GAG-GCC | GAG-GAC | AGG-GAG | GCA-GAG | GAG-AAG | AUA-AGG | CUU-GAA | AAG-GAG |
|  |  |  | 1 | 0.8169 | 0.69014 | 0.69014 | 0.64789 | 0.6338 | 0.6338 | 0.6338 | 0.60563 | 0.60563 |
|  |  | WG | GAG-GAG | CUU-GAG | AGG-GAG | GAU-GAG | GAG-AUA | CUU-GAA | CUU-GCA | CUC-AUA | GAG-AGG | AGG-AUA |
|  |  |  | 1 | 0.78834 | 0.76155 | 0.75683 | 0.71954 | 0.70011 | 0.67752 | 0.67279 | 0.66754 | 0.64968 |
| 48 | Methanothermococcusokinawensis IH1 | AAM | AAA-GAA | AAA-AAA | UUA-AAA | AUA-AAA | AAA-AAU | GAA-AAA | AAA-AUA | AAU-AAA | GAU-AAA | GAU-UUA |
|  |  |  | 1 | 0.995 | 0.95 | 0.905 | 0.9 | 0.88 | 0.815 | 0.72 | 0.65 | 0.615 |
|  |  | CM | UUA-AAA | AAA-AAU | GAA-AAA | AAA-AAA | AUA-AAA | AAU-AAA | AAA-AUA | GAU-AAA | AAA-UAU | AAA-GAA |
|  |  |  | 1 | 0.99167 | 0.91667 | 0.88333 | 0.78333 | 0.75833 | 0.75 | 0.71667 | 0.7 | 0.68333 |
|  |  | EPC | GAA-AAA | AAA-AAA | UUA-AAA | AAA-GAA | AUA-AAA | AAA-AAU | AAA-AUA | AUU-GAA | AAU-AAA | GAA-GAA |
|  |  |  | 1 | 0.9402 | 0.83389 | 0.81395 | 0.76412 | 0.701 | 0.65781 | 0.6113 | 0.60465 | 0.60133 |
|  |  | NM | AAA-AAA | AUA-AAA | UUA-AAA | AAA-AAU | GAA-AAA | AAA-AUA | AAA-GAA | GAU-AAA | AUA-AAU | AAA-GAU |
|  |  |  | 1 | 0.9902 | 0.96078 | 0.95098 | 0.93137 | 0.83333 | 0.78431 | 0.73529 | 0.62745 | 0.62745 |
|  |  | Tr | AAA-AAA | AAA-GAA | GAA-AAA | UUA-AAA | AAU-AAA | AUA-AAA | GAU-AAA | AAA-UUA | AAA-AUA | GAU-GAA |
|  |  |  | 1 | 0.81301 | 0.80488 | 0.71545 | 0.69919 | 0.69106 | 0.63415 | 0.63415 | 0.62602 | 0.55285 |
|  |  | WG | AAA-AAA | AAA-AAU | UUA-AAA | GAA-AAA | AUA-AAA | AAA-GAA | AAA-AUA | AAU-AAA | GAU-AAA | GAA-AAU |
|  |  |  | 1 | 0.87274 | 0.85867 | 0.8085 | 0.78617 | 0.75038 | 0.73937 | 0.73295 | 0.56225 | 0.54206 |
| 49 | Methanothermusfervidus V24S, DSM 2088 | AAM | AAA-AAA | AAA-GAA | GAA-AAA | AAA-AUA | AAA-AUU | UUA-AAA | AAA-AAU | AUA-AAA | GAA-GAA | GAA-AUA |
|  |  |  | 1 | 0.69128 | 0.66443 | 0.61074 | 0.5906 | 0.52685 | 0.49664 | 0.47987 | 0.44295 | 0.40604 |
|  |  | CM | AAA-AAA | AAA-AUA | AAA-GAA | GAA-GAA | GAA-AAA | AUA-AAA | UUA-AAA | AAA-AUU | AAA-AAU | GAA-AAU |
|  |  |  | 1 | 0.70161 | 0.68548 | 0.58871 | 0.58871 | 0.57258 | 0.55645 | 0.55645 | 0.53226 | 0.48387 |
|  |  | EPC | GAA-AAA | AAA-AAA | AAA-GAA | GAA-GAA | AUA-AAA | AAA-AUA | AAA-AAU | AAA-AUU | AAA-GAU | GAA-AUA |
|  |  |  | 1 | 0.95113 | 0.86466 | 0.71429 | 0.70677 | 0.67669 | 0.66917 | 0.59774 | 0.55639 | 0.55263 |
|  |  | NM | AAA-AAA | AAA-GAA | AAA-AUA | GAA-AAA | AAA-AUU | AAA-AAU | AUA-AAA | GAA-AUA | AAA-UUA | GAA-GAA |
|  |  |  | 1 | 0.75352 | 0.73944 | 0.6831 | 0.61972 | 0.52817 | 0.5 | 0.48592 | 0.48592 | 0.46479 |
|  |  | Tr | AAA-AAA | GAA-AAA | GAA-GAA | AAA-AUA | AAA-GAA | AUA-AAA | GAA-AUA | AAA-AAU | UUA-AAA | AAA-UUA |
|  |  |  | 1 | 0.82407 | 0.73148 | 0.69444 | 0.67593 | 0.55556 | 0.53704 | 0.52778 | 0.51852 | 0.51852 |
|  |  | WG | AAA-AAA | GAA-AAA | AAA-GAA | AAA-AUA | AAA-AAU | AUA-AAA | AAA-AUU | GAA-GAA | UUA-AAA | AAA-UUA |
|  |  |  | 1 | 0.77285 | 0.74473 | 0.65974 | 0.58759 | 0.56496 | 0.54418 | 0.52736 | 0.51666 | 0.46683 |
| 50 | Methanotorrisformicicus Mc-S-70 | AAM | AAA-GAA | AAA-AAA | AUA-AAA | GAA-AAA | UUA-AAA | GUU-GUU | GAU-GUU | AAA-AUA | GUU-GAU | GAA-GAA |
|  |  |  | 1 | 0.85098 | 0.81569 | 0.74902 | 0.73725 | 0.62745 | 0.60392 | 0.55686 | 0.55294 | 0.55294 |
|  |  | CM | UUA-AAA | AAA-GAA | AAA-AAA | GAA-AAA | AUA-AAA | GUU-GAU | GAU-GAA | AUU-GAA | AAA-AAU | AAA-GAU |
|  |  |  | 1 | 0.93 | 0.89 | 0.78 | 0.77 | 0.7 | 0.66 | 0.66 | 0.64 | 0.62 |
|  |  | EPC | AAA-GAA | GAA-AAA | AUA-AAA | AAA-AAA | UUA-AAA | AUU-GAA | GUU-GUU | GAA-GUU | GAA-GAA | GUU-GAA |
|  |  |  | 1 | 0.93818 | 0.74182 | 0.73091 | 0.70545 | 0.65091 | 0.62182 | 0.62182 | 0.62182 | 0.60727 |
|  |  | NM | AAA-GAA | AAA-AAA | GAA-AAA | AUA-AAA | UUA-AAA | GUU-GUU | GAU-GAA | GUU-GAU | AUU-GAU | GAU-GUU |
|  |  |  | 1 | 0.7767 | 0.73786 | 0.71845 | 0.70874 | 0.62136 | 0.58252 | 0.56311 | 0.5534 | 0.54369 |
|  |  | Tr | AAA-GAA | AAA-AAA | UUA-AAA | AUA-AAA | GAA-AAA | AAA-AUA | AAA-AUU | AUU-GAA | GAU-GAA | AAA-AAU |
|  |  |  | 1 | 0.93277 | 0.87395 | 0.85714 | 0.79832 | 0.68067 | 0.63866 | 0.58824 | 0.55462 | 0.53782 |
|  |  | WG | AAA-GAA | AAA-AAA | AUA-AAA | GAA-AAA | UUA-AAA | AAA-AAU | AAA-AUA | GAA-GAA | AAA-GAU | GAU-GAA |
|  |  |  | 1 | 0.94995 | 0.87798 | 0.84822 | 0.83088 | 0.66111 | 0.63919 | 0.54498 | 0.544 | 0.54138 |
| 51 | Nanoarchaeumequitans Kin4-M | AAM | AUA-AAA | AAA-GAA | AAA-AAA | UUA-GAA | GAA-AAA | AUA-GAA | AAA-AUA | AAA-GAU | AUA-AUA | UUA-GAU |
|  |  |  | 1 | 1 | 0.86275 | 0.84314 | 0.78431 | 0.76471 | 0.70588 | 0.62745 | 0.54902 | 0.54902 |
|  |  | CM | GAA-AAA | AAA-AUA | AUA-AUA | AAA-GAG | AAA-GAA | UUA-GAU | GAA-GAA | AAA-UAU | UUA-GUU | UUA-GAC |
|  |  |  | 1 | 0.95238 | 0.85714 | 0.71429 | 0.66667 | 0.61905 | 0.57143 | 0.57143 | 0.57143 | 0.52381 |
|  |  | EPC | AUA-GAA | AUA-AAA | GAA-AAA | AAA-GAA | UUA-AAA | GAA-GAA | UUA-GAA | GAA-AUA | AAA-AAA | AAA-AUA |
|  |  |  | 1 | 0.95833 | 0.875 | 0.875 | 0.83333 | 0.79167 | 0.70833 | 0.625 | 0.625 | 0.58333 |
|  |  | NM | AUA-GAA | AAA-AUA | GAA-AAA | UUA-GAA | AUA-AUA | AAA-GAA | GAA-GAA | AUA-AAA | AAA-AAA | GCA-UUA |
|  |  |  | 1 | 0.95238 | 0.85714 | 0.71429 | 0.66667 | 0.61905 | 0.57143 | 0.57143 | 0.57143 | 0.52381 |
|  |  | Tr | GAA-GAA | AAA-GAA | AAA-AAA | GAA-AAA | AUA-AAA | AUA-GAA | GAA-AUA | AAA-AUA | UUA-GAA | AAA-AUU |
|  |  |  | 1 | 1 | 0.86275 | 0.84314 | 0.78431 | 0.76471 | 0.70588 | 0.62745 | 0.54902 | 0.54902 |
|  |  | WG | AUA-AAA | AAA-GAA | AAA-AUA | GAA-AAA | AAA-AAA | AUA-GAA | GAA-AUA | AUA-AUA | GAA-GAA | UUA-GAA |
|  |  |  | 1 | 0.96851 | 0.91031 | 0.88931 | 0.83206 | 0.79389 | 0.69275 | 0.63836 | 0.61832 | 0.6145 |
| 52 | Natrinemapellirubrum 157, JCM 10476 | AAM | CUC-GAG | GCC-GAC | GUC-GAC | GAC-GAC | GAC-GAG | GUC-GUC | GAC-GUC | GAC-GCC | GGC-GAG | GUC-GAG |
|  |  |  | 1 | 0.73589 | 0.66366 | 0.65801 | 0.63995 | 0.60948 | 0.55756 | 0.5395 | 0.51129 | 0.5 |
|  |  | CM | CUC-GAG | GUC-GUC | GCC-GAC | GUC-GAC | GCC-GUC | GUC-GCC | GAC-GGC | GAC-GAC | GCC-GGC | GUC-GGC |
|  |  |  | 1 | 0.87171 | 0.80921 | 0.73684 | 0.71053 | 0.70724 | 0.65789 | 0.65461 | 0.65132 | 0.61842 |
|  |  | EPC | CUC-GAG | GAC-GAC | GAC-GAG | GUC-GUC | GCC-GAC | GUC-GAC | GAG-GAC | GGC-GUC | GAC-GUC | GAC-GGC |
|  |  |  | 1 | 0.76479 | 0.70266 | 0.67899 | 0.67604 | 0.63462 | 0.6213 | 0.59467 | 0.57101 | 0.57101 |
|  |  | NM | CUC-GAG | GAC-GAC | GUC-GAC | GUC-GUC | GCC-GAC | GUC-GAG | GAG-GCC | GGC-GAG | GCC-GAG | GAG-GAG |
|  |  |  | 1 | 0.81513 | 0.71008 | 0.68908 | 0.68908 | 0.55882 | 0.54622 | 0.52941 | 0.52941 | 0.52101 |
|  |  | Tr | CUC-GAG | GAC-GAG | GAC-GAC | GAG-GAC | GAA-CUC | GCC-GAC | GUC-GAC | GAG-GAG | GAG-AUC | GUC-GAG |
|  |  |  | 1 | 0.76546 | 0.71649 | 0.62371 | 0.62371 | 0.55155 | 0.5232 | 0.51289 | 0.49742 | 0.43299 |
|  |  | WG | CUC-GAG | GAC-GAC | GCC-GAC | GAC-GAG | GUC-GUC | GUC-GAC | GAC-GGC | GAG-GAC | GUC-GAG | GAC-GCC |
|  |  |  | 1 | 0.7871 | 0.68671 | 0.68631 | 0.6434 | 0.61535 | 0.51887 | 0.50733 | 0.4955 | 0.49404 |
| 53 | Pyrobaculumaerophilum IM2 | AAM | GAG-GAG | GAG-GCG | GUG-GAG | GAG-GUG | GAG-GCC | GUG-GCG | GUG-GCC | GCC-GUG | GGC-GGC | GCC-GAG |
|  |  |  | 1 | 0.70192 | 0.69712 | 0.66346 | 0.65865 | 0.55769 | 0.53846 | 0.53365 | 0.50962 | 0.50481 |
|  |  | CM | GAG-GCG | GAG-GAG | GAG-GCC | GUG-GAG | UUA-AAA | GCG-GCG | GAC-GAG | GGC-GUG | GGC-GCC | GAC-GGC |
|  |  |  | 1 | 0.95775 | 0.90141 | 0.84507 | 0.73239 | 0.73239 | 0.71831 | 0.70423 | 0.70423 | 0.70423 |
|  |  | EPC | GAG-GAG | GUG-GAG | GAG-GCG | GAG-GUG | GAG-AGG | GCC-GAG | GAG-GCC | GGC-GGC | GCG-GCG | UAC-GGC |
|  |  |  | 1 | 0.60944 | 0.57511 | 0.56652 | 0.53648 | 0.5279 | 0.52361 | 0.50644 | 0.48498 | 0.45923 |
|  |  | NM | GAG-GAG | GUG-GAG | GAG-GCG | GAG-AGG | GUG-GCG | GAG-GUG | GCC-GAG | GAG-GCC | GUG-GUG | GAG-GGG |
|  |  |  | 1 | 0.85542 | 0.61446 | 0.59036 | 0.54217 | 0.54217 | 0.53012 | 0.51807 | 0.49398 | 0.49398 |
|  |  | Tr | GAG-GAG | GUG-GAG | GAG-GCG | GCC-GAG | GAG-AGG | GAG-CUG | AAA-GAG | AGG-GAG | GAG-GUG | AAG-GCG |
|  |  |  | 1 | 0.91803 | 0.80328 | 0.77049 | 0.7541 | 0.68852 | 0.68852 | 0.67213 | 0.63934 | 0.63934 |
|  |  | WG | GAG-GAG | GUG-GAG | GAG-GCG | GAG-GUG | GAG-AGG | GAG-GCC | GCC-GAG | GCG-GCG | GUG-GCG | GCC-GCC |
|  |  |  | 1 | 0.76299 | 0.66024 | 0.5811 | 0.57874 | 0.56063 | 0.50236 | 0.4878 | 0.48465 | 0.47835 |
| 54 | Pyrobaculumoguniense TE7, DSM 13380 | AAM | GAG-GAG | GUG-GAG | GUG-GUG | GAG-GUG | GAG-GCG | GGC-GCC | GCC-GCC | GCC-AAG | GAG-AGG | GUG-GCG |
|  |  |  | 1 | 0.872 | 0.784 | 0.764 | 0.72 | 0.54 | 0.536 | 0.536 | 0.528 | 0.524 |
|  |  | CM | GAG-GAG | GUG-GAG | GAG-GUG | GUG-GUG | GUG-GCG | GAG-GCG | GCG-GCG | GCC-GUG | GAG-AGG | GAG-CUG |
|  |  |  | 1 | 0.93443 | 0.80328 | 0.7459 | 0.69672 | 0.66393 | 0.65574 | 0.62295 | 0.59836 | 0.59016 |
|  |  | EPC | GAG-GAG | GUG-GAG | GAG-GUG | GUG-GUG | GAG-AAG | GAG-GCG | GCG-GCG | GAG-AGG | GCC-GAG | UAC-GGC |
|  |  |  | 1 | 0.85874 | 0.77323 | 0.7026 | 0.62825 | 0.60967 | 0.59851 | 0.59851 | 0.5948 | 0.55762 |
|  |  | NM | GAG-GAG | GUG-GAG | GAG-GUG | GUG-GUG | GAG-CUG | GAG-GCG | GUG-AGG | GAG-AGG | GCC-GAG | GUG-GCG |
|  |  |  | 1 | 0.95652 | 0.8587 | 0.68478 | 0.61957 | 0.53261 | 0.51087 | 0.51087 | 0.46739 | 0.44565 |
|  |  | Tr | GAG-GAG | GUG-GAG | GAG-GUG | GAG-AAG | AAG-GAG | GAG-GCG | GAG-CUG | GAG-AGG | GUG-GUG | GCC-GAG |
|  |  |  | 1 | 0.75735 | 0.63235 | 0.61029 | 0.59559 | 0.55882 | 0.52941 | 0.52941 | 0.44853 | 0.44118 |
|  |  | WG | GAG-GAG | GUG-GAG | GAG-GUG | GUG-GUG | GAG-GCG | GAG-AGG | GCC-GAG | GCG-GCG | GAG-AAG | GCC-GCC |
|  |  |  | 1 | 0.87789 | 0.7393 | 0.68506 | 0.65937 | 0.56105 | 0.55027 | 0.54868 | 0.54773 | 0.54107 |
| 55 | Pyrococcusabyssi GE5 | AAM | GAG-AAG | GAG-GAG | AUA-GUU | GAG-AUA | AUA-GAG | AAG-GAG | GGA-AUA | AUA-AUA | GUU-GAG | AAG-AUA |
|  |  |  | 1 | 0.89474 | 0.88889 | 0.84211 | 0.83626 | 0.78947 | 0.77778 | 0.77778 | 0.76608 | 0.74269 |
|  |  | CM | GAG-AAG | AAG-GAG | AAG-AAG | AUA-AUA | AUA-AAG | AAG-AUA | GAG-AUA | GAG-GAG | GUU-AAG | GGA-AUA |
|  |  |  | 1 | 0.97727 | 0.95455 | 0.93182 | 0.93182 | 0.93182 | 0.88636 | 0.875 | 0.82955 | 0.82955 |
|  |  | EPC | GAG-AAG | GAG-AUA | AAG-AAG | GAG-GAG | AUA-GAG | AUA-AAG | AAG-GAG | GUU-GAG | GGA-AUA | GAG-AGG |
|  |  |  | 1 | 0.99351 | 0.84416 | 0.83117 | 0.77273 | 0.76623 | 0.75974 | 0.74675 | 0.74026 | 0.72727 |
|  |  | NM | GAG-AUA | GAG-AAG | AAG-GAG | GAG-GAG | AAG-AUA | AUA-GAG | GUU-GAG | GAG-AGG | GAA-GAG | AUA-AUA |
|  |  |  | 1 | 0.9 | 0.82857 | 0.8 | 0.8 | 0.74286 | 0.71429 | 0.71429 | 0.7 | 0.7 |
|  |  | Tr | GAG-AAG | AAG-AAG | GAG-GAG | GAG-AUA | AUA-GAG | GAG-GAA | GAG-AGG | AAG-GAG | GAA-AAG | AAG-AUA |
|  |  |  | 1 | 0.875 | 0.77885 | 0.75962 | 0.71154 | 0.68269 | 0.68269 | 0.68269 | 0.66346 | 0.65385 |
|  |  | WG | GAG-AAG | GAG-AUA | GAG-GAG | AAG-AAG | AAG-GAG | AUA-GAG | AAG-AUA | AUA-AAG | AUA-AUA | GAA-GAG |
|  |  |  | 1 | 0.90873 | 0.86911 | 0.8335 | 0.82548 | 0.78335 | 0.75928 | 0.7347 | 0.72668 | 0.69659 |
| 56 | Pyrococcusfuriosus DSM 3638 | AAM | GAA-GAA | AAA-GAA | GAA-GUU | GGA-AUA | GAA-AAG | GAA-AAA | GGA-AAA | GAA-GAG | GGA-AUU | GUU-GAA |
|  |  |  | 1 | 0.8956 | 0.86264 | 0.80769 | 0.74725 | 0.71978 | 0.69231 | 0.69231 | 0.64286 | 0.63187 |
|  |  | CM | GGA-AUA | UUU-GGA | GAA-GAG | AUA-AAG | GAA-GAA | GAA-GUU | GGA-GGA | GGA-GUU | GAA-AAG | AAA-GAA |
|  |  |  | 1 | 0.93478 | 0.93478 | 0.93478 | 0.91304 | 0.90217 | 0.88043 | 0.86957 | 0.83696 | 0.81522 |
|  |  | EPC | GAA-GAA | GAA-AAG | GAA-GAG | GAA-GUU | GGA-AUA | AAG-AAG | GCA-AUA | GGA-AUU | AAG-GAA | GAA-AUU |
|  |  |  | 1 | 0.95139 | 0.86111 | 0.79861 | 0.78472 | 0.75694 | 0.75 | 0.70833 | 0.66667 | 0.64583 |
|  |  | NM | GAA-GAA | GAA-GUU | GAA-AAG | GAG-GAA | GGA-GUU | GUU-GAG | GUU-GAA | AAG-AUA | GGA-AUA | GAA-GAG |
|  |  |  | 1 | 0.875 | 0.8125 | 0.79688 | 0.78125 | 0.70313 | 0.70313 | 0.70313 | 0.6875 | 0.6875 |
|  |  | Tr | GAA-GAA | GAA-AAG | GAA-GAG | GAA-AAA | GAG-AAG | AAG-AAG | GAG-GAA | GAA-GUU | AAA-GAA | GAA-AUA |
|  |  |  | 1 | 0.85556 | 0.78889 | 0.76667 | 0.62222 | 0.62222 | 0.61111 | 0.61111 | 0.61111 | 0.6 |
|  |  | WG | GAA-GAA | GAA-AAG | GAA-GAG | GAA-GUU | AAA-GAA | GAA-AAA | GAG-GAA | GGA-AUA | AAG-AAG | AUA-AAG |
|  |  |  | 1 | 0.86591 | 0.83899 | 0.83845 | 0.79645 | 0.78406 | 0.69036 | 0.68928 | 0.68767 | 0.67528 |
| 57 | Pyrococcushorikoshii OT3 | AAM | AAG-GAG | GGA-AUA | GAA-GUU | AAG-GAA | GAA-AAG | AAG-GUU | AAG-AAG | GAG-AAG | GAA-GAG | AUA-GUU |
|  |  |  | 1 | 0.94488 | 0.93701 | 0.82677 | 0.7874 | 0.7874 | 0.7874 | 0.77953 | 0.75591 | 0.75591 |
|  |  | CM | AUA-AUA | GAG-GAA | AAG-GAA | GAA-GUU | AUA-GAG | GGA-AAG | AAG-GAU | GAG-AAG | AUA-AAG | AAG-AAG |
|  |  |  | 1 | 0.91765 | 0.90588 | 0.87059 | 0.84706 | 0.82353 | 0.82353 | 0.8 | 0.78824 | 0.78824 |
|  |  | EPC | AAG-AAG | GAG-AAG | AAG-AUA | GAG-GAA | AUA-GAG | GAA-GUU | GAG-GAG | AUA-AUA | AUA-AAG | GGA-AUA |
|  |  |  | 1 | 0.90517 | 0.87931 | 0.81034 | 0.81034 | 0.80172 | 0.7931 | 0.76724 | 0.76724 | 0.74138 |
|  |  | NM | GUU-GAG | GAA-GUU | GAG-AAG | AAG-GAA | GAG-AUA | GAA-AAG | GAA-AAA | AUA-GUU | AUA-AUA | GAG-GAG |
|  |  |  | 1 | 0.97872 | 0.91489 | 0.91489 | 0.85106 | 0.85106 | 0.85106 | 0.85106 | 0.82979 | 0.80851 |
|  |  | Tr | GAA-AAG | GAG-AAG | GAG-GAG | GAG-GAA | AAG-AAG | GAA-GUU | GAG-AUA | AGG-GAA | GAA-GAG | AAG-GAG |
|  |  |  | 1 | 0.82474 | 0.7732 | 0.74227 | 0.73196 | 0.68041 | 0.65979 | 0.65979 | 0.61856 | 0.59794 |
|  |  | WG | GAG-AAG | GAG-GAG | AAG-AAG | GAG-GAA | AAG-GAG | AUA-AUA | GAG-AUA | GAA-AAG | GAA-GUU | GAA-GAG |
|  |  |  | 1 | 0.99857 | 0.995 | 0.99357 | 0.99143 | 0.97929 | 0.95929 | 0.95857 | 0.94643 | 0.915 |
| 58 | Pyrolobusfumarii 1A, DSM 11204 | AAM | GAG-GAG | GAG-AAG | GAG-GCU | GGC-GAG | GUC-GAG | GUA-GAG | GCC-GAG | GAG-AUA | GAG-AGG | CUA-GAG |
|  |  |  | 1 | 0.80909 | 0.75 | 0.57727 | 0.52727 | 0.52273 | 0.51818 | 0.50455 | 0.48182 | 0.47273 |
|  |  | CM | GAG-GAG | GAG-GCU | GAG-AAG | CUA-GAG | GAC-GAG | GCC-GAG | CUC-GAG | GAG-GUU | GGC-GAG | GAG-AUA |
|  |  |  | 1 | 0.87931 | 0.82759 | 0.62069 | 0.58621 | 0.55172 | 0.55172 | 0.53448 | 0.51724 | 0.51724 |
|  |  | EPC | GAG-GAG | GAG-AAG | GAG-GCU | GGC-GAG | GAG-AGG | GCC-GAG | GAG-AUA | AAG-AAG | CUC-GAG | GUC-GAG |
|  |  |  | 1 | 0.89157 | 0.8012 | 0.69277 | 0.56627 | 0.53012 | 0.5241 | 0.5 | 0.48795 | 0.48193 |
|  |  | NM | GAG-GAG | GAG-AAG | GAG-GCU | CUC-GAG | GCC-GAG | GAG-AGG | GAG-GGU | GAG-CUU | GUC-GAG | GGC-GAG |
|  |  |  | 1 | 0.83146 | 0.67416 | 0.64045 | 0.57303 | 0.55056 | 0.50562 | 0.50562 | 0.46067 | 0.46067 |
|  |  | Tr | GAG-GAG | GAG-AAG | GAG-AGG | CUC-GAG | GAG-GCU | GAG-AUA | GAC-GAG | AAG-AAG | GAG-CUU | AUA-GAG |
|  |  |  | 1 | 0.69286 | 0.52857 | 0.46429 | 0.42857 | 0.42857 | 0.40714 | 0.35714 | 0.35 | 0.34286 |
|  |  | WG | GAG-GAG | GAG-AAG | GAG-GCU | GAG-AGG | CUC-GAG | GGC-GAG | GAC-GAG | GUC-GAG | GAG-AUA | CUA-GAG |
|  |  |  | 1 | 0.74187 | 0.63274 | 0.53795 | 0.49493 | 0.4673 | 0.43652 | 0.43582 | 0.42462 | 0.41728 |
| 59 | Staphylothermushellenicus P8, DSM 12710 | AAM | GAA-AUA | GAG-AAA | AAA-AUA | GAA-GCA | AGA-AUA | GGA-UUA | AAG-AAA | AUA-AUA | GAU-GAA | GAU-AAA |
|  |  |  | 1 | 0.86139 | 0.83168 | 0.68317 | 0.67327 | 0.66337 | 0.65347 | 0.64356 | 0.63366 | 0.61386 |
|  |  | CM | AAA-AUA | GAA-AUA | AUA-GAU | AUA-UAU | AGA-AUA | AUA-AUA | GGA-UUA | GAG-AAA | AUA-GUU | GAA-AAA |
|  |  |  | 1 | 0.9 | 0.88 | 0.85 | 0.82 | 0.78 | 0.75 | 0.74 | 0.71 | 0.7 |
|  |  | EPC | GAG-AAA | AAA-AUA | GAA-GAA | AUA-GAU | GAU-AAA | AUA-GUU | GAA-AAA | GGA-AUA | GAA-AUA | AGA-AUA |
|  |  |  | 1 | 0.86139 | 0.75248 | 0.74257 | 0.72277 | 0.71287 | 0.70297 | 0.67327 | 0.67327 | 0.67327 |
|  |  | NM | AAA-AUA | AUA-GAU | GAG-AAA | GAA-AUA | GAA-GAA | AUA-GUU | AAA-GCA | AUU-GAU | AAG-AAA | GGA-AUA |
|  |  |  | 1 | 0.98039 | 0.88235 | 0.84314 | 0.80392 | 0.80392 | 0.78431 | 0.76471 | 0.66667 | 0.64706 |
|  |  | Tr | AAA-AUA | GAA-GAA | GAA-AUA | GAG-AAA | GAA-AAA | AAA-CUA | AGA-AUA | AUA-AUA | AUA-GAG | AUA-GAA |
|  |  |  | 1 | 0.97183 | 0.90141 | 0.78873 | 0.71831 | 0.69014 | 0.66197 | 0.59155 | 0.57746 | 0.57746 |
|  |  | WG | AAA-AUA | GAA-AUA | GAG-AAA | GAA-GAA | GAA-AAA | AUA-AUA | AGA-AUA | AUA-GAU | AUA-GUU | AAG-AAA |
|  |  |  | 1 | 0.8756 | 0.79287 | 0.79227 | 0.76449 | 0.75906 | 0.73611 | 0.72947 | 0.66546 | 0.66184 |
| 60 | Staphylothermusmarinus F1, DSM 3639 | AAM | GAA-AUA | GAG-AAA | AAA-AUA | GAA-GAA | AGA-AUA | GGA-UUA | GAA-AAA | GAU-GAA | GAA-GCA | AAG-AAA |
|  |  |  | 1 | 0.95652 | 0.93478 | 0.8913 | 0.8913 | 0.8587 | 0.84783 | 0.75 | 0.73913 | 0.72826 |
|  |  | CM | AAA-AUA | GAA-AUA | AUA-GAU | GAA-GAA | GAG-AAA | AGA-AUA | GAU-AAA | AUA-AUA | GAA-AAA | AAA-CUA |
|  |  |  | 1 | 0.9899 | 0.92929 | 0.91919 | 0.85859 | 0.85859 | 0.83838 | 0.80808 | 0.79798 | 0.78788 |
|  |  | EPC | GAG-AAA | AAA-AUA | GAA-GAA | AUA-GAU | GAA-GCA | GAA-AUA | AUA-GUU | GGA-UUA | AUA-AUA | GAA-AAA |
|  |  |  | 1 | 0.9386 | 0.85088 | 0.81579 | 0.71053 | 0.71053 | 0.71053 | 0.69298 | 0.68421 | 0.67544 |
|  |  | NM | AAA-AUA | GAA-AUA | GAA-GAA | AUA-GAU | AUU-GAU | GAG-AAA | AUA-GUU | GAA-AAA | GGA-AUA | AUA-AUU |
|  |  |  | 1 | 0.9375 | 0.91667 | 0.85417 | 0.83333 | 0.72917 | 0.70833 | 0.6875 | 0.64583 | 0.64583 |
|  |  | Tr | GAA-GAA | GAA-AUA | AAA-AUA | GAG-AAA | AAG-AAA | AUA-GAU | AGA-AUA | AAA-GAA | GAU-GAA | GAA-AAA |
|  |  |  | 1 | 0.79221 | 0.74026 | 0.62338 | 0.62338 | 0.58442 | 0.58442 | 0.58442 | 0.55844 | 0.54545 |
|  |  | WG | AAA-AUA | GAA-GAA | GAA-AUA | GAG-AAA | GAA-AAA | AUA-AUA | AGA-AUA | AUA-GAU | GAU-AAA | AUA-UAU |
|  |  |  | 1 | 0.86599 | 0.85884 | 0.81537 | 0.7838 | 0.78201 | 0.74151 | 0.71173 | 0.66766 | 0.66647 |
| 61 | Sulfolobusacidocaldarius 98-3, DSM 639 | AAM | AAA-AUA | AUA-AUA | GAA-AUA | AAG-AAA | AAG-AUA | AUA-AAG | AUA-AAA | GUA-AUA | AAG-GAA | GAA-AAA |
|  |  |  | 1 | 0.89683 | 0.88095 | 0.88095 | 0.85714 | 0.84921 | 0.83333 | 0.8254 | 0.8254 | 0.80952 |
|  |  | CM | AUA-AUA | AUA-GGU | AAA-AUA | GGA-AUA | AUA-GGA | GAA-AUA | AUA-AUU | UUA-GGU | GGU-AUA | AUA-UUU |
|  |  |  | 1 | 0.79 | 0.71 | 0.69 | 0.68 | 0.67 | 0.66 | 0.64 | 0.64 | 0.63 |
|  |  | EPC | AUA-AAG | GAA-AUA | GGA-AUA | GCA-AUA | AAG-AAA | AUA-GGU | AUA-AUA | AAA-AUA | AAG-AUA | UUA-GGU |
|  |  |  | 1 | 0.94118 | 0.91597 | 0.90756 | 0.89076 | 0.88235 | 0.87395 | 0.84874 | 0.84034 | 0.78992 |
|  |  | NM | AAA-AUA | GAA-AUA | AUA-AUA | GGU-AUA | GAA-AAA | AUA-AAG | GAG-GAA | AAG-GAG | GAA-GAA | GGA-AUA |
|  |  |  | 1 | 0.9434 | 0.9434 | 0.90566 | 0.88679 | 0.86792 | 0.84906 | 0.84906 | 0.83019 | 0.79245 |
|  |  | Tr | GAA-AUA | AUA-GAA | AAG-AAA | AAG-AUA | AUA-GAU | AAA-AUA | GAU-AUA | AUA-AUA | AAG-GAA | GAA-UUA |
|  |  |  | 1 | 0.95652 | 0.91304 | 0.88406 | 0.84058 | 0.84058 | 0.81159 | 0.81159 | 0.7971 | 0.78261 |
|  |  | WG | AUA-AUA | AAA-AUA | GAA-AUA | AAG-AAA | AUA-AAG | GAA-AAA | GAG-GAA | AAG-AUA | AAG-GAA | AUA-GAU |
|  |  |  | 1 | 0.96639 | 0.93342 | 0.79056 | 0.77052 | 0.74467 | 0.71299 | 0.70782 | 0.70394 | 0.70136 |
| 62 | Sulfolobusislandicus M.14.25 | AAM | AUA-AUA | GCA-AUA | GAA-AUA | GGA-AUA | AAA-AUA | AUA-GUA | AUA-AUU | AUA-GCU | GUA-AUA | UUA-AUA |
|  |  |  | 1 | 0.98361 | 0.96721 | 0.89617 | 0.87432 | 0.80874 | 0.79781 | 0.78689 | 0.77596 | 0.75956 |
|  |  | CM | AUA-AUA | GCA-AUA | UUA-AUA | GGA-AUA | AUA-AUU | GUA-AUA | AUA-GGA | AUA-GCU | AAA-AUA | UUA-GGA |
|  |  |  | 1 | 0.72571 | 0.64 | 0.61143 | 0.60571 | 0.58857 | 0.58857 | 0.56 | 0.56 | 0.54857 |
|  |  | EPC | AUA-AUA | GGA-AUA | GAA-AUA | AAG-GAA | AAA-AUA | AUA-GAU | GCA-AUA | AUA-AAG | GAG-GAA | GAA-AAA |
|  |  |  | 1 | 0.85526 | 0.82456 | 0.79386 | 0.7807 | 0.76754 | 0.75 | 0.73246 | 0.7193 | 0.71053 |
|  |  | NM | GAA-AAA | AUA-AUA | AUA-GAA | AAA-AUA | GAA-AUA | GAG-AAA | AAA-GAA | GAA-UUA | UUA-GAA | AAG-GAA |
|  |  |  | 1 | 0.94737 | 0.92105 | 0.92105 | 0.89474 | 0.88158 | 0.88158 | 0.86842 | 0.82895 | 0.82895 |
|  |  | Tr | GAA-AAA | AUA-AUA | AUA-GAA | AAA-AUA | GAA-AUA | GAG-AAA | AAA-GAA | GAA-UUA | UUA-GAA | AAG-GAA |
|  |  |  | 1 | 0.94737 | 0.92105 | 0.92105 | 0.89474 | 0.88158 | 0.88158 | 0.86842 | 0.82895 | 0.82895 |
|  |  | WG | AUA-AUA | GAA-AUA | AAA-AUA | AUA-AUU | AAG-GAA | UUA-AUA | AUA-GAU | GAA-AAA | AUA-AAA | GGA-AUA |
|  |  |  | 1 | 0.85228 | 0.85135 | 0.72554 | 0.7055 | 0.69245 | 0.68593 | 0.67335 | 0.6589 | 0.65005 |
| 63 | Sulfolobusislandicus Y.G.57.14 | AAM | GAA-AUA | AUA-AUA | GCA-AUA | GGA-AUA | AAA-AUA | GUA-AUA | AUA-AUU | AUA-GCU | AUA-GAU | AAG-GAA |
|  |  |  | 1 | 0.95628 | 0.92896 | 0.86885 | 0.86339 | 0.79235 | 0.78689 | 0.76503 | 0.7541 | 0.74317 |
|  |  | CM | AUA-AUA | UUA-AUA | GCA-AUA | GGA-AUA | AUA-GGA | UUA-GGA | AUA-AUU | GUA-AUA | AUA-GAU | AUA-GGU |
|  |  |  | 1 | 0.69136 | 0.69136 | 0.67284 | 0.65432 | 0.61728 | 0.60494 | 0.58025 | 0.58025 | 0.56173 |
|  |  | EPC | AUA-AUA | GGA-AUA | GAA-AUA | AUA-GAU | AAA-AUA | AAG-GAA | AUA-AUU | GCA-AUA | GUA-AUA | AUA-AAG |
|  |  |  | 1 | 0.82081 | 0.82081 | 0.80925 | 0.80925 | 0.78613 | 0.78035 | 0.76879 | 0.75145 | 0.73988 |
|  |  | NM | GAA-AUA | AUA-AUA | GGA-AUA | GAA-GAA | AAA-UUA | AAA-GAA | AAA-AUA | AUA-GAA | GAG-AAA | GAA-GUU |
|  |  |  | 1 | 1 | 0.96154 | 0.90385 | 0.86538 | 0.84615 | 0.82692 | 0.80769 | 0.78846 | 0.78846 |
|  |  | Tr | AAA-AUA | GAA-AAA | AUA-GAA | GAA-UUA | GAG-AAA | AAA-GAA | AUA-AUA | UUA-GAA | AAG-GAA | GAA-AUA |
|  |  |  | 1 | 0.97468 | 0.91139 | 0.89873 | 0.88608 | 0.88608 | 0.87342 | 0.8481 | 0.8481 | 0.83544 |
|  |  | WG | AUA-AUA | GAA-AUA | AAA-AUA | AAG-GAA | AUA-AUU | AUA-GAU | UUA-AUA | GAA-AAA | AAG-AAA | GUA-AUA |
|  |  |  | 1 | 0.89229 | 0.86689 | 0.77234 | 0.7413 | 0.73754 | 0.71308 | 0.69567 | 0.68956 | 0.67404 |
| 64 | Sulfolobussolfataricus P2 | AAM | AUA-AUA | GCA-AUA | GGA-AUA | GAA-AUA | AAA-AUA | AUA-GUA | AUA-GAA | GUA-AUA | AUA-AUU | AUA-GCU |
|  |  |  | 1 | 0.92166 | 0.88018 | 0.84793 | 0.84332 | 0.80645 | 0.76959 | 0.76037 | 0.75576 | 0.74194 |
|  |  | CM | AUA-AUA | GCA-AUA | UUA-AUA | AUA-AUU | AAA-AUA | GUA-AUA | GGA-AUA | AUA-GGU | AUA-GGA | AUA-GCU |
|  |  |  | 1 | 0.85802 | 0.80864 | 0.71605 | 0.67284 | 0.66667 | 0.66049 | 0.66049 | 0.65432 | 0.6358 |
|  |  | EPC | AUA-AUA | GAA-AUA | AUA-AUU | GCA-AUA | GUA-AUA | GGA-AUA | AAG-GAA | AUA-AAG | AAA-AUA | UUA-AUA |
|  |  |  | 1 | 0.87363 | 0.86813 | 0.86264 | 0.81319 | 0.79121 | 0.78022 | 0.74725 | 0.74176 | 0.73626 |
|  |  | NM | AUA-AUA | GGA-AUA | GAA-AUA | AAA-AUA | AUA-GAA | AUA-AAA | UUA-GAA | GAA-AUU | AAA-UUA | AUA-AAU |
|  |  |  | 1 | 0.98305 | 0.91525 | 0.84746 | 0.83051 | 0.79661 | 0.76271 | 0.72881 | 0.72881 | 0.71186 |
|  |  | Tr | AAA-AUA | GAA-AUA | AUA-AUA | AAA-GAA | GAA-AAA | GAU-AAA | GAU-AUA | GAG-AAA | AAG-AAA | AUA-AAA |
|  |  |  | 1 | 0.90909 | 0.81818 | 0.79545 | 0.77273 | 0.76136 | 0.75 | 0.75 | 0.75 | 0.71591 |
|  |  | WG | AUA-AUA | GAA-AUA | AAA-AUA | AAG-GAA | AUA-AUU | UUA-AUA | AUA-AAA | AUA-GAU | GAA-AAA | AUA-GAA |
|  |  |  | 1 | 0.89048 | 0.88016 | 0.8061 | 0.75718 | 0.74013 | 0.71724 | 0.70512 | 0.70242 | 0.69165 |
| 65 | Thermococcusbarophilus MP, DSM 11836 | AAM | GAG-CUU | GAA-AAG | GAA-GUU | AAA-GAG | AAG-CUU | AAA-GAA | AUU-GAA | GUU-GAA | UUU-GAA | GAA-GAA |
|  |  |  | 1 | 0.96377 | 0.86232 | 0.86232 | 0.84783 | 0.84783 | 0.78986 | 0.78261 | 0.74638 | 0.74638 |
|  |  | CM | AAA-GAA | GAG-CUU | GAA-AAA | CUU-GGA | AUU-GAA | GGA-AAA | GAA-AAG | AAG-CUU | UUU-GAG | GCA-AAG |
|  |  |  | 1 | 0.9537 | 0.87037 | 0.86111 | 0.7963 | 0.78704 | 0.78704 | 0.78704 | 0.75926 | 0.75926 |
|  |  | EPC | GAA-GAG | GAG-CUU | AAA-GAA | GUU-GAA | GAA-AAA | AUU-GAA | GAA-AAG | GCA-AAG | GAA-GUU | GAG-GAA |
|  |  |  | 1 | 0.92857 | 0.92063 | 0.88889 | 0.88889 | 0.84921 | 0.84127 | 0.83333 | 0.83333 | 0.8254 |
|  |  | NM | AAA-GAA | GAA-AAG | GAA-AAA | AUU-GAA | AAA-GUU | GUU-GAA | GAA-GUU | GAA-GAA | AAA-GAG | GUU-GAG |
|  |  |  | 1 | 0.89831 | 0.89831 | 0.86441 | 0.83051 | 0.81356 | 0.76271 | 0.69492 | 0.69492 | 0.67797 |
|  |  | Tr | GAA-AAG | GAG-CUU | CUU-GAA | GAA-GAG | AAG-CUU | GAA-GAA | GAA-AAA | AUU-GAA | GAG-GAG | GAG-AAG |
|  |  |  | 1 | 0.82569 | 0.81651 | 0.76147 | 0.74312 | 0.72477 | 0.72477 | 0.69725 | 0.68807 | 0.65138 |
|  |  | WG | GAG-CUU | GAA-AAA | AAA-GAA | GAA-AAG | GAA-GAA | AUU-GAA | AAA-GAG | GAA-GAG | AAG-CUU | GAA-GUU |
|  |  |  | 1 | 0.97562 | 0.95748 | 0.94048 | 0.89116 | 0.88492 | 0.86905 | 0.86168 | 0.78628 | 0.78401 |
| 66 | Thermococcussibiricus MM 739 | AAM | GAA-GAA | AAA-GAA | GAA-AAA | GAA-GAG | CUU-GAA | AUA-AAA | GAA-GUU | GAA-AAG | GGA-AAA | AUU-GAA |
|  |  |  | 1 | 0.89759 | 0.81325 | 0.73494 | 0.66265 | 0.64458 | 0.61446 | 0.61446 | 0.59036 | 0.59036 |
|  |  | CM | AAA-GAA | GAA-GAA | GAA-AAA | GAA-GAG | AUU-GAA | GAA-AAG | AAA-GAG | UUU-GAA | AUA-AAA | GAG-CUU |
|  |  |  | 1 | 0.92857 | 0.87755 | 0.76531 | 0.69388 | 0.66327 | 0.65306 | 0.63265 | 0.61224 | 0.59184 |
|  |  | EPC | AAA-GAA | GAA-GAA | GAA-GAG | GAA-AAG | GAA-AAA | AAA-GAG | GCA-AUA | GAG-AAA | GGA-GAA | CUU-GAA |
|  |  |  | 1 | 0.95238 | 0.89796 | 0.86395 | 0.83673 | 0.7551 | 0.69388 | 0.69388 | 0.68027 | 0.65306 |
|  |  | NM | AAA-GAG | GAA-AAA | AAA-GAA | GAA-GAA | AAA-AAA | AUU-GAA | GCA-AAA | GGA-AAA | GAU-GAA | AUA-GCA |
|  |  |  | 1 | 0.98276 | 0.93103 | 0.82759 | 0.82759 | 0.81034 | 0.72414 | 0.7069 | 0.7069 | 0.7069 |
|  |  | Tr | AAA-GAA | GAA-AAA | GAA-GAG | GAA-GAA | AAA-GAG | GAG-CUU | GAA-AAG | AUU-GAA | AAA-AAA | GAA-CUU |
|  |  |  | 1 | 0.93069 | 0.75248 | 0.70297 | 0.64356 | 0.60396 | 0.59406 | 0.58416 | 0.58416 | 0.55446 |
|  |  | WG | GAA-GAA | AAA-GAA | GAA-AAA | GAA-GAG | AAA-GAG | AUA-AAA | GAA-AAG | AUU-GAA | CUU-GAA | AAA-AAA |
|  |  |  | 1 | 0.99949 | 0.9702 | 0.74049 | 0.69322 | 0.65827 | 0.64902 | 0.61871 | 0.60534 | 0.59455 |
| 67 | Thermogladiuscellulolyticus 1633 | AAM | GAG-GAG | GUC-GAG | GAC-GAG | GAG-AAG | GAG-GUC | GUC-UAC | GAG-UUC | GUC-UUC | CUC-GAG | AAG-UAC |
|  |  |  | 1 | 0.95968 | 0.89516 | 0.84677 | 0.76613 | 0.75806 | 0.69355 | 0.67742 | 0.67742 | 0.66935 |
|  |  | CM | GAC-GAG | GAG-GAG | CUC-GAG | GAG-GUC | GUC-UAC | GAG-UAC | GUC-GAG | GGC-GAG | GAG-AAG | GUC-AAG |
|  |  |  | 1 | 0.95833 | 0.83333 | 0.80556 | 0.72222 | 0.72222 | 0.70833 | 0.69444 | 0.69444 | 0.68056 |
|  |  | EPC | GAG-GAG | GUC-GAG | GAG-AGG | GAG-AAG | GAG-GUC | GUC-UAC | GAG-GCC | CUC-GAG | GUG-GAG | GUC-AAG |
|  |  |  | 1 | 0.84472 | 0.81988 | 0.75155 | 0.73292 | 0.68323 | 0.61491 | 0.60248 | 0.59627 | 0.56522 |
|  |  | NM | GAG-GAG | GAG-AAG | GUC-GAG | GAG-GUC | CUC-GAG | GAG-UAC | GUC-GAC | GCC-GAG | GAG-CUG | GAG-GCC |
|  |  |  | 1 | 0.8806 | 0.8209 | 0.68657 | 0.62687 | 0.61194 | 0.59701 | 0.59701 | 0.59701 | 0.58209 |
|  |  | Tr | GAG-AAG | GAG-AGG | GAC-GAG | GUC-GAG | AGG-GAG | AAG-GAG | GAG-GAC | GAG-AUA | CUC-GAG | AAG-AGG |
|  |  |  | 1 | 0.64815 | 0.51852 | 0.51852 | 0.48148 | 0.44444 | 0.44444 | 0.43519 | 0.43519 | 0.42593 |
|  |  | WG | GAG-GAG | GUC-GAG | GAG-AAG | GAC-GAG | GAG-AGG | GUC-UAC | GAG-GUC | CUC-GAG | GAG-UAC | AGG-AGG |
|  |  |  | 1 | 0.78268 | 0.75621 | 0.66299 | 0.6481 | 0.615 | 0.60838 | 0.5786 | 0.56371 | 0.54937 |
| 68 | Thermoplasmaacidophilum DSM 1728 | AAM | GGC-AUA | AUA-AUA | GCC-AUA | UUC-AUA | GAG-AUA | AUA-AAG | AUA-UUC | AUG-AAG | AUA-GGC | AUA-CUG |
|  |  |  | 1 | 0.96429 | 0.78571 | 0.7 | 0.67143 | 0.67143 | 0.65714 | 0.65 | 0.64286 | 0.62857 |
|  |  | CM | UUC-AUA | AUA-AUA | GGC-AUA | AUA-UUC | UUC-UUC | GCC-AUA | AUA-AUG | AUA-GGC | AUA-UAC | CUG-AUA |
|  |  |  | 1 | 0.88182 | 0.85455 | 0.80909 | 0.77273 | 0.75455 | 0.7 | 0.67273 | 0.63636 | 0.62727 |
|  |  | EPC | GGC-AUA | AAG-AAG | AUA-AAG | AAG-AUA | AUA-AUA | AUA-GGC | GAG-AUA | CUG-AAG | AUA-UUC | GAU-CUG |
|  |  |  | 1 | 0.98261 | 0.94783 | 0.93913 | 0.84348 | 0.82609 | 0.81739 | 0.78261 | 0.76522 | 0.74783 |
|  |  | NM | AUA-AAG | AUA-AUA | GAG-AUA | AAG-AUA | AUA-GAU | AGG-AUA | AAG-AAG | GGC-AUA | GAG-AAG | AUA-GAG |
|  |  |  | 1 | 0.88281 | 0.84375 | 0.73438 | 0.70313 | 0.69531 | 0.69531 | 0.66406 | 0.65625 | 0.64844 |
|  |  | Tr | AUA-AAG | AUA-AUA | GAG-AUA | GAG-AAG | AGG-AUA | AAG-AAG | AAG-AUA | GAG-GAG | AUA-GAG | GAG-GAU |
|  |  |  | 1 | 0.87879 | 0.81818 | 0.81818 | 0.80303 | 0.80303 | 0.77273 | 0.75758 | 0.75758 | 0.71212 |
|  |  | WG | AUA-AUA | AUA-AAG | GAG-AUA | AAG-AUA | AAG-AAG | GGC-AUA | UUC-AUA | GAU-CUG | AUA-GAG | AGG-AUA |
|  |  |  | 1 | 0.84904 | 0.83602 | 0.83448 | 0.80843 | 0.78467 | 0.75249 | 0.70421 | 0.69502 | 0.68966 |
| 69 | Thermoproteusneutrophilus V24Sta | AAM | GAG-GAG | GUG-GAG | GAG-GUG | GUG-GUG | GCC-GCC | GUG-GCG | GCG-GCG | CUG-GAG | AGG-GAG | GAG-GCG |
|  |  |  | 1 | 0.95736 | 0.91085 | 0.84496 | 0.75581 | 0.64729 | 0.64341 | 0.63953 | 0.6124 | 0.58915 |
|  |  | CM | GUG-GAG | GAG-GAG | GAG-GUG | GCC-GCC | GCG-GCG | GAG-AGG | GCC-GAG | GUG-GUG | GAG-GCC | CUG-GAG |
|  |  |  | 1 | 0.93182 | 0.88636 | 0.86364 | 0.69318 | 0.69318 | 0.625 | 0.61364 | 0.59091 | 0.57955 |
|  |  | EPC | GAG-GAG | GUG-GAG | GUG-GUG | GAG-GUG | GCC-GCC | GAG-GCC | GCG-GCG | GCC-GAG | GAG-GCG | AGG-GAG |
|  |  |  | 1 | 0.89958 | 0.72385 | 0.70293 | 0.67782 | 0.58996 | 0.57322 | 0.57322 | 0.57322 | 0.56485 |
|  |  | NM | GAG-GAG | GAG-GUG | GUG-GAG | AGG-GAG | GUG-GUG | CUG-GAG | GAG-CUG | GAG-AGG | GAG-GCG | GCG-GAG |
|  |  |  | 1 | 0.92366 | 0.87786 | 0.68702 | 0.60305 | 0.46565 | 0.45802 | 0.45038 | 0.44275 | 0.43511 |
|  |  | Tr | GAG-GAG | GUG-GAG | GAG-GUG | AGG-GAG | GAG-AGG | GUG-GUG | AGG-AGG | CUG-GAG | GAG-CUG | GAG-AAG |
|  |  |  | 1 | 0.84733 | 0.63359 | 0.51908 | 0.51145 | 0.48092 | 0.46565 | 0.43511 | 0.42748 | 0.41221 |
|  |  | WG | GAG-GAG | GUG-GAG | GAG-GUG | GUG-GUG | GCC-GCC | AGG-GAG | GCG-GCG | GAG-AGG | GAG-GCG | AGG-AGG |
|  |  |  | 1 | 0.9396 | 0.79163 | 0.7063 | 0.69319 | 0.61361 | 0.58325 | 0.56312 | 0.5596 | 0.53787 |
| 70 | Thermosphaeraaggregans M11TL, DSM 11486 | AAM | GUU-GAA | GAG-GAG | GAG-AAG | UUG-AAG | GUU-GAG | AAG-AAG | UUG-AAA | GAG-AAA | AAG-GAG | GAG-GUU |
|  |  |  | 1 | 0.89831 | 0.86441 | 0.84746 | 0.84746 | 0.84746 | 0.83051 | 0.83051 | 0.77966 | 0.71186 |
|  |  | CM | GUU-GAA | GAG-AAG | UUG-AAA | UUG-AAG | GUU-AAA | GAG-AAA | AAA-AUA | GAG-GAA | GAG-GAG | AAG-GAG |
|  |  |  | 1 | 0.88235 | 0.86275 | 0.84314 | 0.84314 | 0.82353 | 0.80392 | 0.76471 | 0.72549 | 0.70588 |
|  |  | EPC | GAG-AAG | GAG-GAG | GAG-AAA | GUU-AAA | GUU-GAA | AAG-GAG | GUU-GAG | AGG-GUU | GAG-GAA | UUG-AAA |
|  |  |  | 1 | 0.88421 | 0.77895 | 0.76842 | 0.75789 | 0.68421 | 0.67368 | 0.6 | 0.58947 | 0.57895 |
|  |  | NM | GAG-GAG | GUU-AAA | GAG-AAG | GUU-GAA | GGG-GUU | AGG-GAG | GAG-GUU | UUG-AAA | GAG-GAA | AAG-GUU |
|  |  |  | 1 | 0.82353 | 0.82353 | 0.73529 | 0.70588 | 0.70588 | 0.67647 | 0.64706 | 0.64706 | 0.61765 |
|  |  | Tr | GAG-GAG | AAG-GAG | GAG-AAG | AGG-GAG | GAG-GUU | GAG-AAA | UUG-AAA | GAG-AUA | GUU-AAA | AUA-GAG |
|  |  |  | 1 | 0.75758 | 0.72727 | 0.62121 | 0.51515 | 0.51515 | 0.43939 | 0.42424 | 0.40909 | 0.39394 |
|  |  | WG | GAG-GAG | GAG-AAG | GUU-GAA | GAG-AAA | UUG-AAA | AAG-GAG | GUU-AAA | GUU-GAG | GAG-GUU | AAG-AAG |
|  |  |  | 1 | 0.90376 | 0.78653 | 0.76815 | 0.68679 | 0.68679 | 0.66754 | 0.65179 | 0.64042 | 0.6238 |
| 71 | Vulcanisaetadistributa DSM 14429 | AAM | GUU-AGG | AGG-GUU | GUU-AAG | AGG-GAG | GAG-GAG | GAG-GUU | AUU-AGG | GGU-GUU | AUU-AAG | GUU-GAU |
|  |  |  | 1 | 0.96602 | 0.93204 | 0.91262 | 0.90291 | 0.87864 | 0.81553 | 0.78155 | 0.76699 | 0.74757 |
|  |  | CM | GUU-AGG | GAG-GAG | GGU-GGU | AGG-AGG | AUA-AUA | GAG-GUU | AGG-GUU | GUU-GGU | AGG-GGU | GUU-AAG |
|  |  |  | 1 | 0.94872 | 0.90598 | 0.87179 | 0.86325 | 0.84615 | 0.84615 | 0.81197 | 0.81197 | 0.79487 |
|  |  | EPC | GUU-AAG | GAG-GAG | GUU-AGG | AUU-AAG | AGG-GAG | GGU-AAG | GUU-GAG | CUU-GAG | GAG-GUU | AUU-AGG |
|  |  |  | 1 | 1 | 0.83665 | 0.8008 | 0.749 | 0.73705 | 0.72112 | 0.70916 | 0.7012 | 0.69721 |
|  |  | NM | AGG-GAG | GAG-GAG | GUU-AGG | AGG-GUU | GAG-GUU | GUU-GAG | AUU-AAG | AGG-AGG | GUU-AAG | AGG-GAU |
|  |  |  | 1 | 0.83333 | 0.78205 | 0.71795 | 0.70513 | 0.64103 | 0.64103 | 0.64103 | 0.61538 | 0.60256 |
|  |  | Tr | GAG-GAG | AUU-AGG | GUU-AAG | GUU-AGG | GUU-GAG | AUU-GAG | AGG-GAG | GGU-AAG | AUU-AAG | AGG-GUU |
|  |  |  | 1 | 0.81522 | 0.80435 | 0.79348 | 0.71739 | 0.70652 | 0.69565 | 0.66304 | 0.6413 | 0.63043 |
|  |  | WG | GAG-GAG | GUU-AAG | GUU-AGG | AGG-GAG | GAG-GUU | AGG-GUU | AUU-AAG | AUU-AGG | AGG-AGG | AUU-GAG |
|  |  |  | 1 | 0.93657 | 0.91833 | 0.88806 | 0.83416 | 0.81965 | 0.81758 | 0.78109 | 0.76824 | 0.74461 |
